# Association Study of Coronary Artery Disease-Associated Genome-Wide Significant SNPs with Coronary Stenosis in Pakistani Population

**DOI:** 10.1155/2020/9738567

**Published:** 2020-06-27

**Authors:** Asma Naseer Cheema, Dilek Pirim, Xingbin Wang, Jabar Ali, Attya Bhatti, Peter John, Eleanor Feingold, F. Yesim Demirci, M. Ilyas Kamboh

**Affiliations:** ^1^Atta-Ur-Rahman School of Applied Biosciences, National University of Sciences & Technology, H/12, Islamabad, Pakistan; ^2^Department of Pathology, Children's Hospital & The Institute of Child Health Multan, Pakistan; ^3^Bursa Uludag University, Faculty of Arts and Sciences, Department of Molecular Biology and Genetics, Bursa, Turkey; ^4^Department of Human Genetics, Graduate School of Public Health, University of Pittsburgh, PA 15261, USA; ^5^Department of Cardiology, Lady Reading Hospital, Peshawar, Pakistan

## Abstract

Genome-wide association studies (GWAS) of coronary artery disease (CAD) have revealed multiple genetic risk loci. We assessed the association of 47 genome-wide significant single-nucleotide polymorphisms (SNPs) at 43 CAD loci with coronary stenosis in a Pakistani sample comprising 663 clinically ascertained and angiographically confirmed cases. Genotypes were determined using the iPLEX Gold technology. All statistical analyses were performed using R software. Linkage disequilibrium (LD) between significant SNPs was determined using SNAP web portal, and functional annotation of SNPs was performed using the RegulomeDB and Genotype-Tissue Expression (GTEx) databases. Genotyping comparison was made between cases with severe stenosis (≥70%) and mild/minimal stenosis (<30%). Five SNPs demonstrated significant associations: three with additive genetic models *PLG*/rs4252120 (*p* = 0.0078), *KIAA1462*/rs2505083 (*p* = 0.005), and *SLC22A3*/rs2048327 (*p* = 0.045) and two with recessive models *SORT1*/rs602633 (*p* = 0.005) and *UBE2Z*/rs46522 (*p* = 0.03). *PLG*/rs4252120 was in LD with two functional *PLG* variants (rs4252126 and rs4252135), each with a RegulomeDB score of 1f. Likewise, *KIAA1462*/rs2505083 was in LD with a functional SNP, *KIAA1462*/rs3739998, having a RegulomeDB score of 2b. In the GTEx database, *KIAA1462*/rs2505083, *SLC22A3*/rs2048327, *SORT1*/rs602633, and *UBE2Z*/rs46522 SNPs were found to be expression quantitative trait loci (eQTLs) in CAD-associated tissues. In conclusion, five genome-wide significant SNPs previously reported in European GWAS were replicated in the Pakistani sample. Further association studies on larger non-European populations are needed to understand the worldwide genetic architecture of CAD.

## 1. Introduction

Coronary artery disease (CAD) is more prevalent among South Asians than any other ethnic groups [1, 2]. Strong familial aggregation, twin studies, and established genetic associations have confirmed the important role of genetics in CAD [3–7]. Dyslipidemia has been identified as a major risk factor for CAD [8], and growing evidence shows that endothelial dysfunction, persistent inflammatory response, and impaired coagulation cascade also play central roles in the initiation and progression of the disease [9–11]. Genome-wide association studies (GWAS) for CAD carried out in populations of European descent have identified multiple loci [12, 13] that highlight the potential involvement of many pathways in CAD pathogenesis. It is of great importance to replicate reported GWAS associations in independent samples and different populations to assess the generalization of such associations.

The aim of this study was to perform a replication study of CAD-associated genome-wide significant single-nucleotide polymorphisms (SNPs) reported among Europeans in a Pakistani sample comprising clinically ascertained and angiographically confirmed cases. We evaluated 47 genome-wide significant SNPs at 43 CAD loci that are involved in lipid metabolism, inflammation, coagulation, and endothelial function. Since most of the genome-wide significant SNPs are located in noncoding regions which are important for gene regulation [14–16], we also performed functional annotations of significant SNPs using the RegulomeDB and Genotype-Tissue Expression (GTEx) databases.

## 2. Materials and Methods

The study cohort consisted of 663 ethnically Pathan subjects (22% female; mean age ± SD: 54 ± 11 years), who presented with chest pain to the Cardiology Unit of the Lady Reading Hospital, Peshawar, Pakistan and were enrolled consecutively for one year after obtaining written informed consent. All subjects were assessed by coronary angiography either to confirm or rule out the CAD. We stratified the study participants based on the coronary angiographic findings: those with ≥70% stenosis were classified as having severe coronary stenosis (*n* = 506), and those with <30% stenosis were considered as having mild/minimal coronary stenosis (*n* = 157). Only those CAD patients who had a first episode of disease and had neither started lipid lowering nor antihypertensive, anti-inflammatory, or anticoagulant drugs were included in the study. 98% of the subjects in both groups had a sedentary life style, and only 2% were taking exercise regularly. In both groups of subjects, carbohydrates were the major energy source (60%) along with saturated fats (30%) and proteins (10%). The Institutional Review Board approved the study. Average blood pressure was obtained after two readings. Height and weight were measured, and electrocardiograph (ECG) was performed. All enrolled subjects were also asked about family history of CAD and smoking.

### 2.1. Biochemical Profile

Five ml of blood was drawn in a plain tube, and serum was analyzed for high-density lipoprotein cholesterol (HDL-C), triglycerides (TG), total cholesterol (TC), blood sugar fasting (BSF), and creatinine. LDL-C was calculated using the Friedewald equation in samples with TG < 400 mg/dl.

### 2.2. Genetic Variant Selection

Information on 47 genome-wide significant SNPs (*p* < 5 × 10^−8^) that were selected for replication in our Pakistani sample is provided in Table S1. The selected loci/genes had putative roles in lipid metabolism, coagulation, inflammation, and endothelial dysfunction [17].

### 2.3. Genotyping

A sample of blood (5 ml) was taken in an EDTA tube from each subject to be processed for DNA extraction. Genomic DNA was isolated from leukocytes using the Qiagen DNeasy Kit. Five to 10 ng of DNAs dried on 384-well plates was processed for genotyping using the iPLEX Gold technology at the University of Pittsburgh Genomics and Proteomics Core Laboratories. The quality of the genotype data was checked for reproducibility by repeating 10% of the samples.

### 2.4. Statistical Analysis

The basic quantitative traits of cases and controls were compared by an independent sample *t*-test and qualitatively by the Chi-square test. The Hardy-Weinberg Equilibrium (HWE) test was performed on all genotype data, and variants with a HWE *p* value < 0.001 were removed from the analysis. The association of SNPs with CAD was determined by logistic regression analysis under additive and recessive genetic models using sex, age, and family history of CAD as covariates (Tables S2 and S3). Family history of CAD was used as a covariate because this is an independent risk factor for CAD, especially in South Asians [18]. Statistical analyses for genetic association were performed using R software (https://www.r-project.org/). The Benjamini-Hochberg's false discovery rate (FDR) method [19] was employed for multiple testing corrections, and an FDR value (*q* value) of <0.20 was considered statistically significant along with nominal *p* < 0.05.

### 2.5. Linkage Disequilibrium (LD) and Functional Annotation of Significant SNPs

Significant SNPs and their proxies were assessed for their putative functional effects on gene expression using the SNAP web portal (https://data.broadinstitute.org/mpg/snpsnap/app/bootface2.py) to determine the closely linked SNPs (*r*^2^ ≥ 0.80) followed by their regulatory effect assessment in RegulomeDB (online database: http://regulome.stanford.edu/). The eQTLs of significant SNPs were assessed by the Genotype-Tissue Expression (GTEx) database (https://gtexportal.org/home/). The RegulomeDB scoring scheme consists of 6 categories: category 1 indicates the strongest evidence for variants to be regulatory by affecting transcription factor (TF) binding and linking to the expression of a gene target; category 2 indicates variants likely to affect TF binding; category 3 indicates variants less likely to affect TF binding; and categories 4-6 indicate variants with minimal TF-binding evidence [20, 21].

## 3. Results

### 3.1. Demographic Data and Biochemical Profile

The basic characteristics and biochemical profiles of 506 subjects with severe coronary stenosis and 157 subjects with mild/minimal coronary stenosis are provided in Table 1. We observed statistically significant differences between the two groups for age, body mass index (BMI), blood pressure (BP), LDL-cholesterol (LDL-C), and family history of CAD (Table 1).

### 3.2. Association Analysis

After performing all QC-filtering measures for genotype data, the average call rate was 98% for genotyped SNPs and none of them deviated significantly from HWE. Logistic regression analysis under the additive genetic model (Table S2) revealed 3 nominally significant SNPs with *p* < 0.05 (*PLG*/rs4252120, *KIAA1462*/rs2505083, and *SLC22A3-LPAL2-LPA*/rs2048327) and 2 SNPs showing a trend for association with *p* < 0.10 (*SORT1*/rs602633 and *SMG6*/rs2281727) (Table 2). Due to the high rate of consanguinity in Pakistan, we also analyzed the data using a recessive model (Table S3). Interestingly, two SNPs (*SORT1*/rs602633, *UBE2Z*/rs46522) including the one that showed a trend of association in the additive model became significant (*p* < 0.05) and two (*COL4A1-COL4A2*/rs4773144, *CYP17A1-CNNM2-NT5C2*/rs12413409) showed a trend of association (*p* < 0.10) (Table 3). Out of these five SNPs, two remained significant after controlling for FDR: *PLG*/rs4252120 (OR = 1.71, *p* = 0.007, *q* = 0.174) and *KIAA1462*/rs2505083 (OR = 1.32, *p* = 0.005, *q* = 0.174). We compared the frequency and association direction of the effect alleles of the five significant variants between Europeans and Pakistanis and found that only *PLG*/rs4252120 differed in the risk allele (T versus C) between the two population groups.

### 3.3. Functional Analysis

We used the RegulomeDB and GTEx databases to determine the functional nature of the five significant SNPs and those in LD with these SNPs. Proxy SNPs and eQTLs for the five significant SNPs (rs2505083, rs4252120, rs2048327, rs602633, and rs46522) are listed in Table S4. *PLG*/rs4252120, with a RegulomeDB score of 6, was not functional itself. However, it was in complete LD (*r*^2^ = 1) with two *PLG* variants located in intron 11 (rs4252126) and intron 12 (rs4252135), each with a RegulomeDB score of 1f, indicating that these SNPs likely affect TF binding and are also potentially linked to the expression of gene targets (eQTL). Of these two *PLG* SNPs, rs4252126 affects the binding of CTCF, RUNX3, TEAD4, and RAN21, while rs4252135 affects the binding of CTCF, FOXA1, NFKB1, RAD21, ZNF263, SMC3, ZNF143, and FOXA2. *KIAA1462*/rs2505083, with a RegulomeDB score of 5, was also not functional. Rather, it was in LD with rs3739998 (*r*^2^ = 0.87) having a RegulomeDB score of 2b, indicating that this likely affects TF binding. rs373998 is located in exon 2 of *KIAA1462* and affects the binding of CTCF, MYC, PAX5, and ZNF143. *SLC22A3*/rs2048327 was neither functional itself nor in LD with any functional SNP. *SORT1*/rs602633 is not functional but in LD with three functional SNPs (rs646776, *r*^2^ = 0.86; rs12740374, *r*^2^ = 0.86; and rs629302, *r*^2^ = 0.85). UBE2Z/rs46522 was functional itself and also in LD with 12 functional SNPs (Table S4).

The GTEx database provides CAD-related tissue expression data for 4 of the 5 significant SNPs: *KIAA1462*/rs2505083, *SLC22A3*/rs2048327, *SORT1*/rs602633, and *UBE2Z*/rs46522 (Table S4). *KIAA1462*/rs2505083 was found to be an eQTL for the *KIAA1462* gene (*p* = 7.20*E*‐18) in the aorta artery that surpassed the genome-wide significance threshold of *p* = <5*E*‐08. *SLC22A3*/rs2048327 was an eQTL for the *SLC22A3* gene in the left ventricle (*p* = 2.40*E*‐08). *SORT1*/rs602633 was significantly linked to an eQTL of one gene only (*PSRC1* in whole blood; *p* = 5.9*E*‐15). *UBE2Z*/rs46522 was observed as an eQTL for three genes (*UBE2Z* in whole blood, *p* = 1.50*E*‐44; *SNF8* in the aorta, *p* = 9.4*E*‐08; and *ATP5G1* in the left ventricle, *p* = 2.30*E*‐15) (Table 4, Table S4).

## 4. Discussion

In this study, we sought the replication of 47 previously GWAS-implicated CAD risk variants among Europeans in the Pakistani population and found a significant association of five of the variants with coronary stenosis (*PLG*/rs4252120, *KIAA1462*/rs2505083, *SLC22A3-LPAL2-LPA*/rs2048327, *SORT1*/rs602633, and *UBE2Z*/rs46522).

The comparison of frequency, type, and direction of effect alleles of five significant SNPs with Europeans showed a difference only for *PLG*/rs4252120, where C was the risk allele in Pakistanis but T was the risk allele in European populations [17]. The reason of this discrepancy is not clear. A possible explanation may be due to different patterns of LD between Pakistanis and Europeans. Based on the RegulomeDB score, this variant was not functional but rather in LD with two functional variants. Plasminogen encoded by the *PLG* gene on chromosome 6 breaks down the fibrin clot to ward off the coagulation process. Although the precise mechanism by which *PLG* variants may affect atherosclerosis needs to be elucidated, genetic variation in this gene may delay and disrupt the process of fibrin resolution leading to clot buildup. Hence, the event of myocardial infarction (MI) becomes almost inevitable in the presence of overwhelmed clot formation. *PLG* is located in close proximity to the *LPA* gene on chromosome 6 [22, 23]. *PLG*/rs4252120 has been shown to be associated with plasma levels of Lp(a), and higher Lp(a) level is a risk factor for MI [24, 25]. Additionally, *PLG* has been shown to be part of one of four CAD risk loci (*APOA1*, *MRAS*, *IL6R*, and *PLG*) that are involved in the acute inflammatory response signaling pathway [26].

The second significant variant, *KIAA1462*/rs2505083, is also intronic that has shown association with CAD, independent of lipid levels, smoking, hypertension, and diabetes mellitus [27, 28]. Consistent with prior observations, the association of this variant in our study was also independent of the examined and established nongenetic risk factors, signifying the involvement of a novel pathway in CAD pathogenesis besides hyperlipidemias. According to RegulomeDB, *KIAA1462*/rs2505083 is not a functional SNP itself but it is in LD with *KIAA1462*/rs373998 which is a functional SNP and located in exon 2 of *KIAA1462.* It harbours a missense mutation that results in a serine-to-threonine change at position 1002 for the JCAD protein [27]. In the GTEx eQTL analysis, *KIAA1462*/rs2505083 was associated with the RNA expression difference of the *KIAA1462* gene in the aorta artery. JCAD is considered as a novel component of VE-cadherin (a cell-to-cell junction protein [28]. The interendothelial cell junctions play an important role in vessel wall integrity. Tight junctions (TJs) regulate the paracellular transport of the cell, while adherent junctions (AJs) are responsible for cell adhesion. VE-cadherin is a major adhesion molecule of AJs, responsible for cell adhesion, and JCAD has been identified as an integral part of VE-cadherin [28]. Although it may be premature to predict a causative role of the JCAD protein in CAD pathogenesis, it is noteworthy that the loss of junctional integrity of endothelial cells is the first step for the initiation of atherosclerotic plaque formation. The JCAD-attributed endothelial cell-cell junctional integrity may be critical in maintaining the mechanical strength of the vessel wall, and any dysfunction can lead to the loss of this support and may promote the atherosclerotic process. Endothelial dysfunction due to the loss of adhesion is a relatively new concept in disease pathogenesis, and its potential role in CAD could provide new research avenues.

*SLC22A3*/rs2048327 is located in intron 8 of *SLC22A3*. SLC22A3 is a solute carrier family membrane protein and has a critical role in the elimination of endogenous and organic cations. It may affect the body blood pressure, which is a significant risk factor for CAD. This SNP is most likely functional, as this is an eQTL in the left ventricle of the heart. Sallinen et al. [29] have also provided evidence of the association of *SLC22A3*/rs2048327 in diabetic nephropathy and hypertension.

Because of the relatively high consanguinity rate in the population, the use of the recessive model enabled us to detect two additional associations in the *SORT1* and *UBE2Z* regions. *SORT1*/rs602633 is located downstream of the gene cluster *CELSR2-PSRC1-MYBPHL-SORT1.* It encodes *sortilin*. Kjolby et al. [30] provided the first link between atherosclerosis and sortilin. He demonstrated the deletion of the sortilin protein in the LDL receptor in mice and correlated it with reduced atherosclerotic plaque size [31]. His observation was reinforced by Patel et al. [32]. Sortilin also plays an important role in an inflammatory reaction and foam cell occurrence during atherosclerotic plaque formation as revealed by some of the animal studies [33]. Since our study as well as GWAS showed an association of coronary artery disease with this gene, it would be interesting to provide mechanistic insights of sortilin in humans.

*UBE2Z*/rs46522 is located in intron 2 of the ubiquitin-conjugating enzyme E2 Z gene. Lu et al. showed an association of *UBE2Z*/rs46522 with CAD in the Chinese Han population [34]. Although there is not a single study that has described the direct role of this protein in CAD pathogenesis, the ubiquitin protein is vital in intracellular cell signaling and regulates the important pathways implicated in cell growth and viability [35]. Aberrations in ubiquitin signaling can lead to the pathogenesis of many human diseases, including CAD. The use of a comparable number of CAD samples with that used by us but with a much smaller number of SNPs has been reported in two Pakistani samples. While one study examined 13 CAD risk SNPs in relation to premature CAD (mean age = ~40) assessed by angiography in a total sample of 650 [36], the other study screened 6 SNPs in 624 subjects where CAD cases were assessed only clinically (no angiography) and controls were apparently healthy subjects [37]. While no significant association was observed in the latter case-control study, 5 nominal significant associations were seen in the angiography-assessed premature CAD, including *APOE*, which we have also previously reported in our angiography-assessed sample [38]. Of the other 4 significant SNPs reported by Ansari et al. [36], only one SNP (*MIA3*/rs17465367) overlapped with our SNPs and those of Shahid et al. [37], which was not significant in both studies. Ansari et al. [36] also reported a nominal significant association with *SORT1*/rs646776 (*p* = 0.02), where we also found significant association in *SORT1*, although with a different SNP, rs602633 (*p* = 0.005). Thus, 1 of 5 associations reported by Ansari et al. [36] in an angiography-assessed sample are confirmed in our comparable sample. On the other hand, all of our 4 significant SNPs were not examined by either of the reported studies.

The following limitations should be considered before generalizing our results in the Pakistani population. The sample size was small, and subjects were collected from a cardiology hospital where patients present to the hospital in the advanced stage of their disease. Only a small number of cases with mild/minimal coronary stenosis were available for comparison with severe coronary stenosis. Despite this limitation, we have previously reported a significant association of *APOE* with coronary stenosis in our sample [38] which has been confirmed by another study that also used an angiography-assessed small sample [36]. Recently, more than 160 loci have been implicated with CAD [4, 39]. Due to funding constraints, we used mostly the top GWAS hit in each of the 43 gene regions in our replication study. Often, GWAS help to identify a gene region rather than a specific gene and the identified SNPs are rarely causal. For this reason, more SNPs need to be genotyped in a region to replicate a locus in a different population. Genotyping of additional SNPs in each gene region may have replicated more loci in our population. In summary, we have replicated 5 of the 47 previously reported genome-wide significant SNPs in our sample. The five genes have a role in endothelial dysfunction, coagulation disorder, and hypertension which are critical processes that initiate and sustain CAD. The screening of SNPs in all known CAD gene regions in a much larger sample may help to better understand the genetic risk profile in the Pakistani population that will help improve risk prediction, prevention, and treatment approaches.

## 5. Conclusions

Five genome-wide significant SNPs previously reported in European GWAS were also replicated in the Pakistani sample. Further association studies on larger non-European populations are needed to understand the worldwide genetic architecture of CAD.

## Figures and Tables

**Table 1:** Characteristics of subjects with >70% stenosis vs. ≤30% stenosis.

Parameters	Coronary stenosis (*n* = 506)	Mild/minimal coronary stenosis (*n* = 157)	*p* value
Age (years)	49.12 ± 13.09	55.46 ± 10.81	0.011^∗^
BMI (kg/m^2^)	29.15 ± 4.85	28.24 ± 3.54	0.032^∗^
BSF (mg/dl)	125.79 ± 49.78	113.77 ± 35.37	0.057
Creatinine (mmol/l)	0.98 ± 0.25	0.98 ± 0.19	0.852
BP systolic (mmHg)	132.18 ± 18.33	121.79 ± 19.15	<0.001^∗^
BP diastolic (mmHg)	84.48 ± 10.59	80.19 ± 10.56	<0.001^∗^
TC (mg/dl)	199.60 ± 54.23	187.13 ± 46.08	0.163
HDL-C (mg/dl)	39.03 ± 8.93	41.25 ± 10.63	0.252
LDL-C (mg/dl)	127.17 ± 45.24	105.47 ± 36.61	0.032^∗^
TG (mg/dl)	187.72 ± 57.48	176.61 ± 45.50	0.280
Gender (M/F)%	79/21	75/25	0.321
Smoking (Y/N)%	28/72	24/76	0.364
FH (Y/N)%	38/62	27/73	0.010^∗^

BMI=body mass index; BSF=blood sugar fasting; BP=blood pressure; TC=total cholesterol; HDL-C=high-density lipoprotein cholesterol; LDL-C=low-density lipoprotein cholesterol; TG=triglyceride; FH=family history of CAD. ^∗^Significant difference between the two groups.

**Table 2:** Characteristics of SNPs that showed nominally significant association (*p* < 0.05) or a trend for association (*p* < 0.10) by the additive model.

Chr #	Gene	SNP ID	RA	RAF	OR (95% CI)	*p* value	FDR (*q* value)
10	*PLG*	rs4252120	C	0.168	1.714 (1.161-2.53)	0.00534	0.1744
6	*KIAA1462*	rs2505083	C	0.364	1.322 (1.007-1.736)	0.007267	0.1744
6	*SLC22A3-LPAL2-LPA*	rs2048327	G	0.301	0.7662 (0.577-1.017)	0.045751	0.532787
2	*ABCG5-ABCG8*	rs6544713	T	0.335	1.176 (0.892-1.55)	0.064877	0.632787
10	*CXCL12*	rs2047009	C	0.344	1.203 (0.915-1.329)	0.076773	0.632787
1	*PCSK9*	rs11206510	C	0.084	1.482 (0.889-2.47)	0.079098	0.632787
6	*ANKS1A*	rs17609940	C	0.132	1.389 (0.906-2.128)	0.099173	0.67526

Chr=chromosome; SNP=single-nucleotide polymorphism; RA=risk allele; RAF=risk allele frequency; FDR=false discovery rate (*q* value).

**Table 3:** Characteristics of SNPs that showed nominally significant association (*p* < 0.05) or a trend for association (*p* < 0.10) by recessive model.

Chr #	Gene	SNP ID	RA	RAF	OR (95% CI)	*p* value	FDR (*q* value)
1	*SORT1*	rs602633	A	0.209	0.359 (0.173-0.744)	0.005	0.270
17	*UBE2Z*	rs46522	C	0.499	0.637 (0.423-0.958)	0.003	0.697
13	*COL4A1-COL4A2*	rs4773144	C	0.470	0.656 (428-1.005)	0.052	0.762
10	*CYP17A1-CNNM2-NT5C2*	rs12413409	A	0.169	0.351 (0.115-1.073)	0.066	0.762

Chr=chromosome; SNP=single-nucleotide polymorphism; RA=risk allele; RAF=risk allele frequency; FDR=false discovery rate (*q* value).

**Table 4:** Functional annotation of significant SNPs.

Chr #	Gene	SNP ID	RegulomeDB	eQTL	*p* value	CAD-related tissue expression	Whole blood expression
10	*KIAA1462*	rs2505083	5	KIAA1462	7.20*E*‐18	Aorta and tibial artery	—
		rs3739998	2b		1.90*E*‐16	Aorta	—
6	*PLG*	rs4252120	6	—	—	No tissue expression related to coronary artery disease	
		rs4252126	1f				
		rs4252135	1f				
6	*SLC22A3-LPAL2-LPA*	rs2048327	No data	SLC22A3	2.40*E*‐08	Left ventricle	—
1	*SORT1*	rs602633	7	PSRC1	5.9*E*‐15	—	Whole blood
				SYPL2	0.000005	Aorta	—
				PSRC1	0.000017	Left ventricle	—
		rs646776	1f	PSRC1	2.30*E*‐18	—	Whole blood
				SYPL2	1.00*E*‐07	Left ventricle	—
		rs12740374	2b	PSRC1	4.00*E*‐16	—	Whole blood
		rs629301	1f	PSRC1	2.30*E*‐18	—	Whole blood
17	*UBE2Z*	rs46522	1f	UBE2Z	1.50*E*‐44	—	Whole blood
				SNF8	9.40*E*‐08	Aorta	
				SUMO2P17	5.20*E*‐09	Tibial artery	
				ATP5G1	2.30*E*‐15	Left ventricle	

## Data Availability

The data supporting the results of our study are included in this research paper.

## Supplementary Materials

Supplementary materials Table S1: list of selected variants (identified in Europeans) replicated in the Pakistani population. Table S2: logistic regression analysis of all the SNPs (additive genetic model). Table S3: logistic regression analysis of all the SNPs (recessive genetic model). Table S4: functional annotation of significant SNPs.
