# Supplementary material for: Association Study of Coronary Artery Disease-Associated Genome-Wide Significant SNPs with Coronary Stenosis in Pakistani Population
Source: Dis Markers. 2020 Jun 27;2020:9738567. doi: 10.1155/2020/9738567 (PMC7336215; doi:10.1155/2020/9738567)
Supplement: Supplementary materials — Table S1: list of selected variants (identified in Europeans) replicated in the Pakistani population. Table S2: logistic regression analysis of all the SNPs (additive genetic model). Table S3: logistic regression analysis of all the SNPs (recessive genetic model). Table S4: functional annotation of significant SNPs. [file 9738567.f1.docx]

**Table S1: List of CAD-associated Variants (identified in Europeans) that were selected for replication in Pakistani population [17]**

| **Chr** | **Gene** | **Effect/Non effect (Freq)** | **Position** | **SNP** | **GWAS p-value** |  | **Ethnicity** |
| --- | --- | --- | --- | --- | --- | --- | --- |
| 1p13.3 | SORT1 | C/A(0.77) | 1:109278889 | rs602633 | $1.47\times{10}^{-25}$ |  | Europeans |
| 1p32.3 | PCSK9 | T/C(0.84) | 1:55030366 | rs11206510 | $4.00\times{10}^{-11}$ |  | Europeans |
| 1p32.2 | PPAP2B | A/G (0.91) | 1:56497149 | rs17114036 | $5.80\times{10}^{-12}$ |  | Europeans |
| 1q14 | MIA3 | T/G (0.87) | 1:222650187 | rs17465637 | $1.36\times{10}^{-08}$ |  | Europeans |
| 1q21.3 | IL6R | T/C (0.47) | 1:154449591 | rs4845625 | $3.64\times{10}^{-10}$ |  | Europeans |
| 2q32.3 | WDR12 | C/T (0.11) | 2:202881162 | rs6725887 | $1.16\times{10}^{-15}$ |  | Europeans |
| 2p24.1 | APOB | G/A(0.83) | 2:21063185 | rs515135 | $2.56\times{10}^{-10}$ |  | Europeans |
| 2q22.3 | ZEB2-AC074093.1 | G/A(0.46) | 2:145043894 | rs2252641 | $5.30\times{10}^{-08}$ |  | Europeans |
| 2p11.2 | VAMP5-VAMP8-GGCX | A/G (0.45) | 2:85582866 | rs1561198 | $1.22\times{10}^{-10}$ |  | Europeans |
| 2p21 | ABCG5-ABCG8 | T/C(0.30) | 2:43846742 | rs6544713 | $2.12\times{10}^{-09}$ |  | Europeans |
| 3q22.3 | MRAS | T/C(0.14) | 3:138403280 | rs9818870 | $2.64\times{10}^{-09}$ |  | Europeans |
| 4q32.1 | GUCY1A3 | G/A(0.81) | 4:155714157 | rs7692387 | $2.65\times{10}^{-11}$ |  | Europeans |
| 5q31.1 | SLC22A4-SLC22A5 | C/T(0.14) | 5:132331660 | rs273909 | $9.62\times{10}^{-10}$ |  | Europeans |
| 6q23.2 | TCF21 | C/G (0.59) | 6:133893387 | rs12190287 | $4.94\times{10}^{-13}$ |  | Europeans |
| 6q25.3 | SLC22A3-LPAL2-LPA | G/A (0.35) | 6:160442500 | rs2048327 | $6.86\times{10}^{-11}$ |  | Europeans |
| 6p21.2 | KCNK5 | T/C (0.76) | 6:39207146 | rs10947789 | $9.81\times{10}^{-09}$ |  | Europeans |
| 6q26 | PLG | T/C (0.73) | 6:160722576 | rs4252120 | $4.88\times{10}^{-10}$ |  | Europeans |
| 6q21.31 | ANKS1A | C/G (0.07) | 6:35067023 | rs17609940 | $1.36\times{10}^{-08}$ |  | Europeans |
| 6q24.1 | PHACTR1 | A/C (0.65) | 6:12901209 | rs9369640 | $7.53\times{10}^{-22}$ |  | Europeans |
| 7q32.2 | ZC3HC1 | C/T (0.65) | 7:130023656 | rs11556924 | $6.74\times{10}^{-17}$ |  | Europeans |
| 7p21.2 | HDAC9 | G/A (0.10) | 7:18997152 | rs2023938 | $4.94\times{10}^{-08}$ |  | Europeans |
| 8p21.3 | LPL | G/A (0.86) | 8:19955669 | rs264 | $2.88\times{10}^{-09}$ |  | Europeans |
| 8q24.3 | TRIB1 | A/T (0.55) | 8:125478730 | rs2954029 | $4.75\times{10}^{-09}$ |  | Europeans |
| 9p21.3 | CDKN2BAS1 | A/G (0.50) | 9:22003224 | rs3217992 | $7.75\times{10}^{-57}$ |  | Europeans |
| 9q34.2 | ABO | C/T (0.21) | 9:133278724 | rs579459 | $2.66\times{10}^{-08}$ |  | Europeans |
| 10q24.32 | CYP17A1-CNNM2-NT5C2 | G/A (0.89) | 10:102959339 | rs12413409 | $6.26\times{10}^{-08}$ |  | Europeans |
| 10p11.23 | KIAA1462 | C/T (0.42) | 10:30046193 | rs2505083 | $1.35\times{10}^{-11}$ |  | Europeans, South Asians |
| 10q11.21 | CXCL12 | A/G (0.83) | 10:44258419 | rs501120 | $1.79\times{10}^{-09}$ |  | Europeans |
| 10q11.21 | CXCL12 | C/A (0.48) | 10:44044465 | rs2047009 | $1.59\times{10}^{-09}$ |  | Europeans |
| 10q23.31 | LIPA | T/C (0.38) | 10:89246097 | rs2246833 | $4.35\times{10}^{-08}$ |  | Europeans, South Asians |
| 11q22.3 | PDGFD | A/G (0.29) | 11:103789839 | rs974819 | $3.55\times{10}^{-11}$ |  | Europeans, South Asians |
| 11q23.2 | ZNF259-APOA5-APOA1 | C/G (0.22) | 11:116778201 | rs964184 | $1.02\times{10}^{-17}$ |  | Europeans |
| 12q24.12 | SH2B3 | T/C (0.40) | 12:111446804 | rs3184504 | $5.44\times{10}^{-11}$ |  | Europeans |
| 13q34 | COL4A1-COL4A2 | T/C (0.74) | 13:110397276 | rs9515203 | $1.43\times{10}^{-11}$ |  | Europeans |
| 13q34 | COL4A1-COL4A2 | C/A (0.42) | 13:110308365 | rs4773144 | $5.85\times{10}^{-12}$ |  | Europeans |
| 13q12.3 | FLT1 | A/G (0.32) | 13:28399484 | rs9319428 | $7.32\times{10}^{-11}$ |  | Europeans |
| 14q32.2 | HHIPL1 | C/T (0.43) | 14:99667605 | rs2895811 | $4.08\times{10}^{-10}$ |  | Europeans |
| 15q25.1 | ADAMTS7 | C/T (0.38) | 15:78849442 | Rs7173743 | $6.74\times{10}^{-13}$ |  | Europeans, South Asians |
| 15q26.1 | FURIN-FES | A/C (0.44) | 15:90873320 | rs17514846 | $9.33\times{10}^{-11}$ |  | Europeans |
| 17p11.2 | RAI1-PEMT-RASD1 | G/A (0.59) | 17:17640408 | rs12936587 | $1.24\times{10}^{-09}$ |  | Europeans |
| 17q21.32 | UBE2Z | C/T (0.42) | 17:48911235 | rs46522 | $1.81\times{10}^{-09}$ |  | Europeans |
| 17p13.3 | SMG6 | C/T (0.36) | 17:2214651 | rs2281727 | $7.83\times{10}^{-09}$ |  | Europeans |
| 19q13.32 | ApoE-ApoC1 | G/A (0.14) | 19:44892362 | Rs2075650 | $5.86\times{10}^{-10}$ |  | Europeans |
| 19q13.32 | ApoE-ApoC1 | C/T (0.90) | 19:44912383 | Rs445925 | $8.76\times{10}^{-09}$ |  | Europeans |
| 19p13.2 | LDLR | G/T (0.76) | 19:11052925 | rs1122608 | $6.33\times{10}^{-14}$ |  | Europeans |
| 21q22.11 | KCNE2 | T/C (0.13) | 21:34226827 | rs9982601 | $7.67\times{10}^{-11}$ |  | Europeans |
| - 7q22.3 | BCAP29 | T/C(0.11) | 7:107604100 | rs10953541 | 5.56$\times{10}^{-09}$ |  | Europeans, South Asians |

| **CHR**  **Table S2: Logistic regression analysis of 47 genetic variants by additive model** | **SNP** | **BP** | **A1** | **TEST** | **OR** | **SE** | **L95** | **U95** | **STAT** | **_P-Value** | **FDR** |
| --- | --- | --- | --- | --- | --- | --- | --- | --- | --- | --- | --- |
| 6 | rs4252120 | 160722575 | C | ADD | 1.714 | 0.199 | 1.161 | 2.532 | 2.709 | 0.00534 | 0.1744 |
| 10 | rs2505083 | 30046192 | C | ADD | 1.322 | 0.1388 | 1.007 | 1.736 | 2.014 | 0.007267 | 0.1744 |
| 6 | rs2048327 | 160442499 | G | ADD | 0.7662 | 0.1444 | 0.5774 | 1.017 | -1.845 | 0.045751 | 0.532787 |
| 2 | rs6544713 | 43846741 | T | ADD | 1.176 | 0.1409 | 0.8923 | 1.55 | 1.151 | 0.064877 | 0.632787 |
| 10 | rs2047009 | 44044464 | C | ADD | 1.203 | 0.1392 | 0.9159 | 1.58 | 1.329 | 0.076773 | 0.632787 |
| 1 | rs11206510 | 55030365 | C | ADD | 1.482 | 0.2604 | 0.8898 | 2.47 | 1.512 | 0.079098 | 0.632787 |
| 6 | rs17609940 | 35067022 | C | ADD | 1.389 | 0.2176 | 0.9066 | 2.128 | 1.51 | 0.099173 | 0.67526 |
| 10 | rs2246833 | 89246096 | T | ADD | 1.205 | 0.1344 | 0.9263 | 1.569 | 1.391 | 0.144801 | 0.772272 |
| 17 | rs2281727 | 2214650 | C | ADD | 1.278 | 0.1448 | 0.9624 | 1.698 | 1.695 | 0.174271 | 0.826006 |
| 17 | rs12936587 | 17640407 | A | ADD | 0.9058 | 0.1555 | 0.6679 | 1.229 | -0.6361 | 0.266463 | 0.826006 |
| 7 | rs11556924 | 130023655 | T | ADD | 0.8235 | 0.1615 | 0.6 | 1.13 | -1.202 | 0.272174 | 0.826006 |
| 11 | rs9515203 | 116778200 | G | ADD | 1.231 | 0.1684 | 0.8849 | 1.713 | 1.234 | 0.274455 | 0.826006 |
| 21 | rs9818870 | 34226826 | T | ADD | 1.147 | 0.2704 | 0.6752 | 1.949 | 0.5074 | 0.298691 | 0.826006 |
| 9 | rs3217992 | 22003223 | A | ADD | 0.9533 | 0.1328 | 0.7349 | 1.237 | -0.36 | 0.302628 | 0.826006 |
| 2 | rs6725887 | 202881161 | C | ADD | 1.386 | 0.2721 | 0.8132 | 2.363 | 1.2 | 0.322986 | 0.826006 |
| 1 | rs602633 | 109278888 | A | ADD | 0.7619 | 0.1554 | 0.5619 | 1.033 | -1.75 | 0.340755 | 0.826006 |
| 1 | rs17465637 | 222650186 | A | ADD | 0.8826 | 0.1382 | 0.6732 | 1.157 | -0.9034 | 0.350884 | 0.826006 |
| 6 | rs9319428 | 12901208 | C | ADD | 1.218 | 0.1546 | 0.8997 | 1.649 | 1.276 | 0.352333 | 0.826006 |
| 19 | rs445925 | 44912382 | T | ADD | 0.6611 | 0.2786 | 0.3829 | 1.141 | -1.486 | 0.373296 | 0.826006 |
| 5 | rs273909 | 132331659 | C | ADD | 1.138 | 0.1857 | 0.7907 | 1.638 | 0.6957 | 0.407061 | 0.826006 |
| 10 | rs501120 | 44258418 | G | ADD | 0.8581 | 0.139 | 0.6535 | 1.127 | -1.102 | 0.423824 | 0.826006 |
| 10 | rs12413409 | 102959338 | A | ADD | 0.756 | 0.1773 | 0.534 | 1.07 | -1.578 | 0.425777 | 0.826006 |
| 9 | rs579459 | 133278723 | C | ADD | 1.021 | 0.1704 | 0.7309 | 1.425 | 0.1203 | 0.457981 | 0.826006 |
| 2 | rs1561198 | 85582865 | A | ADD | 1.142 | 0.1332 | 0.8797 | 1.483 | 0.9977 | 0.463752 | 0.826006 |
| 8 | rs2954029 | 125478729 | T | ADD | 0.9498 | 0.1385 | 0.7241 | 1.246 | -0.3716 | 0.473196 | 0.826006 |
| 19 | rs1122608 | 11052924 | T | ADD | 0.9171 | 0.1536 | 0.6786 | 1.239 | -0.5634 | 0.479201 | 0.826006 |
| 1 | rs17114036 | 56497148 | G | ADD | 0.8123 | 0.205 | 0.5436 | 1.214 | -1.014 | 0.481837 | 0.826006 |
| 13 | rs9369640 | 110397275 | C | ADD | 0.9465 | 0.1882 | 0.6545 | 1.369 | -0.292 | 0.533045 | 0.83234 |
| 6 | rs10947789 | 39207145 | C | ADD | 1.13 | 0.1712 | 0.8076 | 1.58 | 0.7122 | 0.533405 | 0.83234 |
| 15 | rs6725887.1 | 78849441 | C | ADD | 1.008 | 0.1384 | 0.7688 | 1.323 | 0.06005 | 0.572733 | 0.83234 |
| 14 | rs2895811 | 99667604 | C | ADD | 0.9096 | 0.1375 | 0.6947 | 1.191 | -0.689 | 0.574438 | 0.83234 |
| 19 | rs2075650 | 44892361 | G | ADD | 1.269 | 0.2039 | 0.8508 | 1.892 | 1.167 | 0.595507 | 0.83234 |
| 12 | rs3184504 | 111446803 | T | ADD | 1.014 | 0.1809 | 0.7115 | 1.446 | 0.07812 | 0.60097 | 0.83234 |
| 17 | rs46522 | 48911234 | C | ADD | 0.8772 | 0.1305 | 0.6792 | 1.133 | -1.004 | 0.606915 | 0.83234 |
| 11 | rs964184 | 103789838 | A | ADD | 0.9403 | 0.138 | 0.7174 | 1.232 | -0.4463 | 0.662709 | 0.847784 |
| 15 | rs17514846 | 90873319 | A | ADD | 1.136 | 0.1361 | 0.8698 | 1.483 | 0.9348 | 0.708792 | 0.847784 |
| 8 | rs264 | 19955668 | A | ADD | 0.8683 | 0.1641 | 0.6295 | 1.198 | -0.8603 | 0.709651 | 0.847784 |
| 4 | rs7173743 | 155714156 | A | ADD | 0.9494 | 0.1756 | 0.6729 | 1.34 | -0.2957 | 0.714376 | 0.847784 |
| 1 | rs4845625 | 154449590 | C | ADD | 0.9889 | 0.1369 | 0.7562 | 1.293 | -0.08148 | 0.723294 | 0.847784 |
| 13 | rs4773144 | 110308364 | C | ADD | 0.8677 | 0.1343 | 0.667 | 1.129 | -1.057 | 0.724148 | 0.847784 |
| 6 | rs12190287 | 133893386 | G | ADD | 1.064 | 0.1339 | 0.8184 | 1.383 | 0.4634 | 0.744647 | 0.851025 |
| 7 | rs2023938 | 18997151 | G | ADD | 1.007 | 0.201 | 0.6791 | 1.493 | 0.03502 | 0.792394 | 0.884533 |
| 3 | rs974819 | 138403279 | T | ADD | 1.084 | 0.2222 | 0.701 | 1.675 | 0.3613 | 0.85405 | 0.916324 |
| 7 | rs10953541 | 107604099 | T | ADD | 0.9972 | 0.1821 | 0.6978 | 1.425 | -0.01559 | 0.859054 | 0.916324 |
| 13 | rs7692387 | 28399483 | A | ADD | 0.9528 | 0.1502 | 0.7098 | 1.279 | -0.3219 | 0.893235 | 0.932071 |
| 2 | rs515135 | 21063184 | A | ADD | 1.023 | 0.1805 | 0.7186 | 1.458 | 0.1286 | 0.943166 | 0.963234 |
| 2 | rs2252641 | 145043893 | A | ADD | 1.068 | 0.1316 | 0.825 | 1.382 | 0.4979 | 0.986634 | 0.986634 |

| **CHR**  **Table S3: Logistic regression analysis of 47 genetic variants by recessive model** | **SNP** | **BP** | **A1** | **TEST** | **OR** | **SE** | **L95** | **U95** | **STAT** | **P-Value** | **FDR** |
| --- | --- | --- | --- | --- | --- | --- | --- | --- | --- | --- | --- |
| 1 | rs602633 | 1.09E+08 | A | REC | 0.3595 | 0.3714 | 0.1736 | 0.7445 | -2.755 | 0.005878 | 0.270388 |
| 17 | rs46522 | 48911234 | C | REC | 0.6372 | 0.2081 | 0.4238 | 0.958 | -2.166 | 0.03031 | 0.69713 |
| 13 | rs4773144 | 1.1E+08 | C | REC | 0.6565 | 0.2172 | 0.4289 | 1.005 | -1.937 | 0.05275 | 0.76291 |
| 10 | rs12413409 | 1.03E+08 | A | REC | 0.3515 | 0.5694 | 0.1152 | 1.073 | -1.836 | 0.06634 | 0.76291 |
| 17 | rs2281727 | 2214650 | C | REC | 1.581 | 0.3173 | 0.8487 | 2.944 | 1.443 | 0.149 | 0.783045 |
| 2 | rs1561198 | 85582865 | A | REC | 1.411 | 0.2607 | 0.8464 | 2.352 | 1.32 | 0.1868 | 0.783045 |
| 6 | rs12190287 | 1.34E+08 | G | REC | 1.352 | 0.2373 | 0.8494 | 2.154 | 1.272 | 0.2033 | 0.783045 |
| 6 | rs9369640 | 12901208 | C | REC | 1.658 | 0.4094 | 0.7429 | 3.698 | 1.234 | 0.2171 | 0.783045 |
| 10 | rs501120 | 44258418 | G | REC | 0.7154 | 0.2853 | 0.409 | 1.251 | -1.174 | 0.2404 | 0.783045 |
| 19 | rs2075650 | 44892361 | G | REC | 3.372 | 1.062 | 0.421 | 27 | 1.145 | 0.2522 | 0.783045 |
| 11 | rs974819 | 1.04E+08 | A | REC | 0.7335 | 0.2756 | 0.4274 | 1.259 | -1.125 | 0.2607 | 0.783045 |
| 9 | rs3217992 | 22003223 | A | REC | 0.7859 | 0.2206 | 0.51 | 1.211 | -1.092 | 0.2746 | 0.783045 |
| 15 | rs17514846 | 90873319 | A | REC | 1.347 | 0.2801 | 0.778 | 2.333 | 1.064 | 0.2874 | 0.783045 |
| 8 | rs264 | 19955668 | A | REC | 0.6434 | 0.4274 | 0.2784 | 1.487 | -1.032 | 0.3022 | 0.783045 |
| 6 | rs4252120 | 1.61E+08 | C | REC | 1.926 | 0.6378 | 0.5517 | 6.722 | 1.027 | 0.3042 | 0.783045 |
| 19 | rs445925 | 44912382 | T | REC | 0.2313 | 1.463 | 0.01315 | 4.07 | -1.001 | 0.317 | 0.783045 |
| 1 | rs4845625 | 1.54E+08 | C | REC | 0.7939 | 0.2374 | 0.4985 | 1.264 | -0.9722 | 0.331 | 0.783045 |
| 6 | rs2048327 | 1.6E+08 | G | REC | 0.7415 | 0.3138 | 0.4008 | 1.372 | -0.9531 | 0.3406 | 0.783045 |
| 2 | rs515135 | 21063184 | A | REC | 1.728 | 0.5817 | 0.5526 | 5.404 | 0.9403 | 0.3471 | 0.783045 |
| 7 | rs10953541 | 1.08E+08 | T | REC | 1.817 | 0.6496 | 0.5086 | 6.49 | 0.9191 | 0.358 | 0.783045 |
| 3 | rs9818870 | 1.38E+08 | T | REC | 2.701 | 1.091 | 0.3181 | 22.94 | 0.9105 | 0.3626 | 0.783045 |
| 17 | rs12936587 | 17640407 | A | REC | 1.48 | 0.4416 | 0.623 | 3.517 | 0.8882 | 0.3745 | 0.783045 |
| 8 | rs2954029 | 1.25E+08 | T | REC | 1.251 | 0.2938 | 0.7032 | 2.224 | 0.7615 | 0.4463 | 0.809321 |
| 21 | rs9982601 | 34226826 | T | REC | 0.3893 | 1.244 | 0.03403 | 4.454 | -0.7587 | 0.4481 | 0.809321 |
| 11 | rs964184 | 1.17E+08 | G | REC | 1.41 | 0.4717 | 0.5595 | 3.555 | 0.7288 | 0.4661 | 0.809321 |
| 15 | rs7173743 | 78849441 | C | REC | 0.8275 | 0.2598 | 0.4973 | 1.377 | -0.7288 | 0.4661 | 0.809321 |
| 13 | rs9515203 | 1.1E+08 | C | REC | 1.672 | 0.8063 | 0.3443 | 8.122 | 0.6377 | 0.5237 | 0.809321 |
| 6 | rs17609940 | 35067022 | C | REC | 1.937 | 1.065 | 0.2403 | 15.61 | 0.6208 | 0.5347 | 0.809321 |
| 7 | rs11556924 | 1.3E+08 | T | REC | 0.7678 | 0.4316 | 0.3295 | 1.789 | -0.6123 | 0.5403 | 0.809321 |
| 19 | rs1122608 | 11052924 | T | REC | 1.277 | 0.401 | 0.5819 | 2.803 | 0.6099 | 0.542 | 0.809321 |
| 1 | rs17465637 | 2.23E+08 | A | REC | 0.8499 | 0.2834 | 0.4877 | 1.481 | -0.5738 | 0.5661 | 0.809321 |
| 1 | rs11206510 | 55030365 | C | REC | 0.6225 | 0.8525 | 0.1171 | 3.31 | -0.556 | 0.5782 | 0.809321 |
| 10 | rs2246833 | 89246096 | T | REC | 1.131 | 0.2224 | 0.7312 | 1.749 | 0.5525 | 0.5806 | 0.809321 |
| 13 | rs9319428 | 28399483 | A | REC | 0.8472 | 0.3496 | 0.427 | 1.681 | -0.4742 | 0.6354 | 0.812957 |
| 6 | rs10947789 | 39207145 | C | REC | 1.252 | 0.4746 | 0.4939 | 3.174 | 0.4736 | 0.6358 | 0.812957 |
| 12 | rs3184504 | 1.11E+08 | T | REC | 0.7687 | 0.5594 | 0.2568 | 2.301 | -0.4703 | 0.6381 | 0.812957 |
| 14 | rs2895811 | 99667604 | C | REC | 0.8944 | 0.249 | 0.549 | 1.457 | -0.4483 | 0.6539 | 0.812957 |
| 2 | rs2252641 | 1.45E+08 | A | REC | 1.094 | 0.243 | 0.6795 | 1.761 | 0.3694 | 0.7118 | 0.861653 |
| 7 | rs2023938 | 18997151 | G | REC | 1.308 | 0.8268 | 0.2587 | 6.612 | 0.3246 | 0.7455 | 0.879308 |
| 9 | rs579459 | 1.33E+08 | C | REC | 1.156 | 0.5117 | 0.4242 | 3.153 | 0.2841 | 0.7763 | 0.888457 |
| 4 | rs7692387 | 1.56E+08 | A | REC | 0.8701 | 0.5308 | 0.3074 | 2.463 | -0.262 | 0.7933 | 0.888457 |
| 2 | rs6544713 | 43846741 | T | REC | 1.072 | 0.2921 | 0.6049 | 1.901 | 0.2389 | 0.8112 | 0.888457 |
| 10 | rs2047009 | 44044464 | C | REC | 0.9582 | 0.2559 | 0.5803 | 1.582 | -0.167 | 0.8674 | 0.927916 |
| 5 | rs273909 | 1.32E+08 | C | REC | 0.923 | 0.5957 | 0.2872 | 2.967 | -0.1345 | 0.893 | 0.933591 |
| 1 | rs17114036 | 56497148 | G | REC | 1.093 | 0.8247 | 0.217 | 5.502 | 0.1075 | 0.9144 | 0.93472 |
| 10 | rs2505083 | 30046192 | C | REC | 1.009 | 0.2673 | 0.5973 | 1.703 | 0.03185 | 0.9746 | 0.9746 |
| 2 | rs6725887 | 2.03E+08 | C | REC | NA | NA | NA | NA | NA | NA | NA |

| **Gene/SNP** | **Proxy SNP** | ***r^2^*** | **RegulomeDB** | ***eQTL Gene***  **Table S4: Functional annotation of significant SNPs** | | **P-value** | | **Tissue** | | | | | | | | | |  |  |  |  |  |  |
| --- | --- | --- | --- | --- | --- | --- | --- | --- | --- | --- | --- | --- | --- | --- | --- | --- | --- | --- | --- | --- | --- | --- | --- |
|  |  |  |  |  |  |  |  |  |  |  |  |  |  |  |  |  |  |  |  |  |  |  |  |
| ***KIAA1462*/** |  |  |  |  | |  | |  | | | | | | | | | |  |  |  |  |  |  |
| **rs2505083** |  |  |  |  | |  | |  | | | | | | | | | |  |  |  |  |  |  |
|  | rs2505083 | 1 | 5 | [*KIAA1462*](javascript:EqtlsPage.methods.gotoGeneExpression('ENSG00000165757.8')) | | 7.20E-18 | | Artery - Aorta | | | | | | | | | |  |  |  |  |  |  |
|  |  |  |  | [*KIAA1462*](javascript:EqtlsPage.methods.gotoGeneExpression('ENSG00000165757.8')) | | 9.90E-07 | | Artery - Tibial | | | | | | | | | |  |  |  |  |  |  |
|  | rs765906 | 0.935 | 6 | *No data* | | No data available | | | | | | | | | | | |  |  |  |  |  |  |
|  | rs2487928 | 0.904 | 4 | [*KIAA1462*](javascript:EqtlsPage.methods.gotoGeneExpression('ENSG00000165757.8')) | | 7.10E-17 | | Artery - Aorta | | | | | | | | | |  |  |  |  |  |  |
|  |  |  |  | [*KIAA1462*](javascript:EqtlsPage.methods.gotoGeneExpression('ENSG00000165757.8')) | | 1.40E-05 | | Artery - Tibial | | | | | | | | | |  |  |  |  |  |  |
|  | rs1887318 | 0.869 | No data | [*KIAA1462*](javascript:EqtlsPage.methods.gotoGeneExpression('ENSG00000165757.8')) | | 1.80E-17 | | Artery - Aorta | | | | | | | | | |  |  |  |  |  |  |
|  |  |  |  | [*KIAA1462*](javascript:EqtlsPage.methods.gotoGeneExpression('ENSG00000165757.8')) | | 8.90E-06 | | Artery - Tibial | | | | | | | | | |  |  |  |  |  |  |
|  | rs2478835 | 0.869 | 6 | [*KIAA1462*](javascript:EqtlsPage.methods.gotoGeneExpression('ENSG00000165757.8')) | | 5.20E-17 | | Artery - Aorta | | | | | | | | | |  |  |  |  |  |  |
|  |  |  |  | [*KIAA1462*](javascript:EqtlsPage.methods.gotoGeneExpression('ENSG00000165757.8')) | | 9.60E-06 | | Artery - Tibial | | | | | | | | | |  |  |  |  |  |  |
|  | **rs3739998** | **0.869** | **2b** | [***KIAA1462***](javascript:EqtlsPage.methods.gotoGeneExpression('ENSG00000165757.8')) | | **1.90E-16** | | **Artery - Aorta** | | | | | | | | | |  |  |  |  |  |  |
|  |  |  |  | [*KIAA1462*](javascript:EqtlsPage.methods.gotoGeneExpression('ENSG00000165757.8')) | | 9.20E-06 | | Artery - Tibial | | | | | | | | | |  |  |  |  |  |  |
|  | rs2487927 | 0.835 | 5 | [*KIAA1462*](javascript:EqtlsPage.methods.gotoGeneExpression('ENSG00000165757.8')) | | 4.70E-15 | | Artery - Aorta | | | | | | | | | |  |  |  |  |  |  |
|  |  |  |  | [*KIAA1462*](javascript:EqtlsPage.methods.gotoGeneExpression('ENSG00000165757.8')) | | 1.30E-05 | | Artery - Tibial | | | | | | | | | |  |  |  |  |  |  |
|  | rs2478839 | 0.835 | 5 | [*KIAA1462*](javascript:EqtlsPage.methods.gotoGeneExpression('ENSG00000165757.8')) | | 3.70E-15 | | Artery - Aorta | | | | | | | | | |  |  |  |  |  |  |
|  |  |  |  | [*KIAA1462*](javascript:EqtlsPage.methods.gotoGeneExpression('ENSG00000165757.8')) | | 1.10E-05 | | Artery - Tibial | | | | | | | | | |  |  |  |  |  |  |
|  | rs2505084 | 0.817 | 5 | [*KIAA1462*](javascript:EqtlsPage.methods.gotoGeneExpression('ENSG00000165757.8')) | | 6.10E-21 | | Artery - Aorta | | | | | | | | | |  |  |  |  |  |  |
|  |  |  |  | [*KIAA1462*](javascript:EqtlsPage.methods.gotoGeneExpression('ENSG00000165757.8')) | | 4.40E-09 | | Artery - Tibial | | | | | | | | | |  |  |  |  |  |  |
| ***PLG*/** |  |  |  |  | |  | |  | | | | | | | | | |  |  |  |  |  |  |
| **rs4252120** |  |  |  |  | |  | |  | | | | | | | | | |  |  |  |  |  |  |
|  | rs4252120 | 1 | 6 | *-* | | No tissue expression related coronary artery disease-Expresses significantly in Testis, Liver, Esophagus | | | | | | | | | | | | |  |  |  |  |  |
|  | rs4252117 | 1 | 6 | *-* | | No tissue expression related coronary artery disease-Expresses significantly in Testis, Liver, Esophagus | | | | | | | | | | | | |  |  |  |  |  |
|  | rs4252114 | 1 | No data | *-* | | No tissue expression related coronary artery disease-Expresses significantly in Testis, Liver, Esophagus | | | | | | | | | | | | |  |  |  |  |  |
|  | rs9458016 | 1 | 5 | *-* | | No tissue expression related coronary artery disease-Expresses significantly in Testis, Liver, Esophagus | | | | | | | | | | | | |  |  |  |  |  |
|  | rs1321197 | 1 | 6 | *-* | | No tissue expression related coronary artery disease-Expresses significantly in Testis, Liver, Esophagus | | | | | | | | | | | | |  |  |  |  |  |
|  | rs9456578 | 1 | 6 | *-* | | No tissue expression related coronary artery disease-Expresses significantly in Testis, Liver, Esophagus | | | | | | | | | | | | |  |  |  |  |  |
|  | rs4252125 | 1 | 3a | *-* | | No tissue expression related coronary artery disease-Expresses significantly in Testis, Liver, Esophagus | | | | | | | | | | | | |  |  |  |  |  |
|  | **rs4252126** | **1** | **1f** | *-* | | No tissue expression related coronary artery disease-Expresses significantly in Testis, Liver, Esophagus | | | | | | | | | | | | |  |  |  |  |  |
|  | rs4252130 | 1 | 6 | *-* | | No tissue expression related coronary artery disease-Expresses significantly in Testis, Liver, Esophagus | | | | | | | | | | | | |  |  |  |  |  |
|  | rs4252134 | 1 | 6 | *-* | | No tissue expression related coronary artery disease-Expresses significantly in Testis, Liver, Esophagus | | | | | | | | | | | | |  |  |  |  |  |
|  | rs9458019 | 1 | No data | *-* | | No tissue expression related coronary artery disease-Expresses significantly in Testis, Liver, Esophagus | | | | | | | | | | | | |  |  |  |  |  |
|  | **rs4252135** | **1** | **1f** | *-* | | No tissue expression related coronary artery disease-Expresses significantly in Testis, Liver, Esophagus | | | | | | | | | | | | |  |  |  |  |  |
|  | rs9458020 | 1 | 5 | *-* | | No tissue expression related coronary artery disease-Expresses significantly in Testis, Liver, Esophagus | | | | | | | | | | | | |  |  |  |  |  |
|  | rs56093624 | 1 | 4 | *-* | | No tissue expression related coronary artery disease-Expresses significantly in Testis, Liver, Esophagus | | | | | | | | | | | | |  |  |  |  |  |
|  | rs4252150 | 1 | 4 | *-* | | No tissue expression related coronary artery disease-Expresses significantly in Testis, Liver, Esophagus | | | | | | | | | | | | |  |  |  |  |  |
|  | rs4252151 | 1 | 4 | *-* | | No tissue expression related coronary artery disease-Expresses significantly in Testis, Liver, Esophagus | | | | | | | | | | | | |  |  |  |  |  |
|  | rs4252165 | 1 | 6 | *-* | | No tissue expression related coronary artery disease-Expresses significantly in Testis, Liver, Esophagus | | | | | | | | | | | | |  |  |  |  |  |
|  | rs1897108 | 0.967 | 6 | *-* | | No tissue expression related coronary artery disease-Expresses significantly in Testis, Liver, Esophagus | | | | | | | | | | | | |  |  |  |  |  |
|  | rs13231 | 0.959 | 6 | *-* | | No tissue expression related coronary artery disease-Expresses significantly in Testis, Liver, Esophagus | | | | | | | | | | | | |  |  |  |  |  |
|  | rs4252109 | 0.959 | 5 | *-* | | No tissue expression related coronary artery disease-Expresses significantly in Testis, Liver, Esophagus | | | | | | | | | | | | |  |  |  |  |  |
|  | rs9458017 | 0.959 | 6 | *-* | | No tissue expression related coronary artery disease-Expresses significantly in Testis, Liver, Esophagus | | | | | | | | | | | | |  |  |  |  |  |
|  | rs4252096 | 0.959 | No data | *No data* | |  | |  |  |  |  |  | |  |  | | | |  |  |  |  |  |
|  | rs4252093 | 0.959 | 6 | *-* | | No tissue expression related coronary artery disease-Expresses significantly in Testis, Liver, Esophagus | | | | | | | | | | | | |  |  |  |  |  |
|  | rs4252090 | 0.959 | No data | *-* | | No tissue expression related coronary artery disease-Expresses significantly in Testis, Liver, Esophagus | | | | | | | | | | | | |  |  |  |  |  |
|  | rs4252087 | 0.959 | 6 | *-* | | No tissue expression related coronary artery disease-Expresses significantly in Testis, Liver, Esophagus | | | | | | | | | | | | |  |  |  |  |  |
|  | rs1853018 | 0.959 | No data | *-* | | No tissue expression related coronary artery disease-Expresses significantly in Testis, Liver, Esophagus | | | | | | | | | | | | |  |  |  |  |  |
|  | rs1972748 | 0.959 | 6 | *No data* | |  | |  |  |  |  |  | |  |  | | | |  |  |  |  |  |
|  | rs4252072 | 0.959 | No data | *-* | | No tissue expression related coronary artery disease-Expresses significantly in Testis, Liver, Esophagus | | | | | | | | | | | | |  |  |  |  |  |
|  | rs28402939 | 0.959 | No data | *-* | | No tissue expression related coronary artery disease-Expresses significantly in Testis, Liver, Esophagus | | | | | | | | | | | | |  |  |  |  |  |
|  | rs4252086 | 0.919 | 6 | *-* | | No tissue expression related coronary artery disease-Expresses significantly in Testis, Liver, Esophagus | | | | | | | | | | | | |  |  |  |  |  |
|  | rs4252107 | 0.881 | 5 | *-* | | No tissue expression related coronary artery disease-Expresses significantly in Testis, Liver, Esophagus | | | | | | | | | | | | |  |  |  |  |  |
|  | rs4252076 | 0.881 | 6 | *-* | | No tissue expression related coronary artery disease-Expresses significantly in Testis, Liver, Esophagus | | | | | | | | | | | | |  |  |  |  |  |
|  | rs4252181 | 0.881 | 5 | *-* | | No tissue expression related coronary artery disease-Expresses significantly in Testis, Liver, Esophagus | | | | | | | | | | | | |  |  |  |  |  |
|  | rs56262039 | 0.881 | 4 | *-* | | No tissue expression related coronary artery disease-Expresses significantly in Testis, Liver, Esophagus | | | | | | | | | | | | |  |  |  |  |  |
|  | rs9456580 | 0.881 | 6 | *-* | | No tissue expression related coronary artery disease-Expresses significantly in Testis, Liver, Esophagus | | | | | | | | | | | | |  |  |  |  |  |
|  | rs4252082 | 0.879 | 6 | *-* | | No tissue expression related coronary artery disease-Expresses significantly in Testis, Liver, Esophagus | | | | | | | | | | | | |  |  |  |  |  |
|  | rs62439805 | 0.843 | 6 | *-* | | No tissue expression related coronary artery disease-Expresses significantly in Testis, Liver, Esophagus | | | | | | | | | | | | |  |  |  |  |  |
|  | rs9458022 | 0.843 | 4 | *-* | | No tissue expression related coronary artery disease-Expresses significantly in Testis, Liver, Esophagus | | | | | | | | | | | | |  |  |  |  |  |
|  | rs4252066 | 0.843 | 6 | *-* | | No tissue expression related coronary artery disease-Expresses significantly in Testis, Liver, Esophagus | | | | | | | | | | | | |  |  |  |  |  |
|  | rs9458012 | 0.839 | 6 | *-* | | No tissue expression related coronary artery disease-Expresses significantly in Testis, Liver, Esophagus | | | | | | | | | | | | |  |  |  |  |  |
|  | rs62436702 | 0.801 | 5 | *-* | | No tissue expression related coronary artery disease-Expresses significantly in Testis, Liver, Esophagus | |  |  |  |  |  |  |  |  |  |  |  |  |  |  |  |  |
| *SLC22A3/* |  |  |  |  | |  | |  | | | | | | | | |  |  |  |  |  |  |  |
| rs2048327 |  |  |  |  | |  | |  | | | | | | | | |  |  |  |  |  |  |  |
|  | rs2048327 | 1 | No data | *SLC22A3* | | 2.40E-08 | | Heart - Left Ventricle | | | | | | | | |  |  |  |  |  |  |  |
|  |  |  |  | *SLC22A3* | | 7.50E-13 | | Artery - Tibial | | | | | | | | |  |  |  |  |  |  |  |
|  | rs9355288 | 1 | 5 | *SLC22A3* | | 2.40E-08 | | Heart - Left Ventricle | | | | | | | | |  |  |  |  |  |  |  |
|  |  |  |  | *SLC22A3* | | 4.70E-13 | | Artery - Tibial | | | | | | | | |  |  |  |  |  |  |  |
|  | rs2292334 | 1 | No data | *SLC22A3* | | 3.20E-07 | | Heart - Left Ventricle | | | | | | | | |  |  |  |  |  |  |  |
|  |  |  |  | *SLC22A3* | | 2.90E-12 | | Artery - Tibial | | | | | | | | |  |  |  |  |  |  |  |
|  | rs9355288 | 0.983 | 5 | *SLC22A3* | | 2.40E-08 | | Heart - Left Ventricle | | | | | | | | |  |  |  |  |  |  |  |
|  |  |  |  | *SLC22A3* | | 4.70E-13 | | Artery - Tibial | | | | | | | | |  |  |  |  |  |  |  |
|  | rs2292334 | 0.984 | No data | *SLC22A3* | | 3.20E-07 | | Heart - Left Ventricle | | | | | | | | |  |  |  |  |  |  |  |
|  |  |  |  | *SLC22A3* | | 2.90E-12 | | Artery - Tibial | | | | | | | | |  |  |  |  |  |  |  |
|  | rs7769879 | 0.987 | No data | *SLC22A3* | | 2.40E-08 | | Heart - Left Ventricle | | | | | | | | |  |  |  |  |  |  |  |
|  |  |  |  | *SLC22A3* | | 7.50E-13 | | Artery - Tibial | | | | | | | | |  |  |  |  |  |  |  |
|  | rs3918285 | 0.969 | No data | *SLC22A3* | | 2.40E-08 | | Heart - Left Ventricle | | | | | | | | |  |  |  |  |  |  |  |
|  |  |  |  | *SLC22A3* | | 4.70E-13 | | Artery - Tibial | | | | | | | | |  |  |  |  |  |  |  |
|  | rs3918286 | 0.959 | No data | *SLC22A3* | | 2.90E-08 | | Heart - Left Ventricle | | | | | | | | |  |  |  |  |  |  |  |
|  |  |  |  | *SLC22A3* | | 7.70E-13 | | Artery - Tibial | | | | | | | | |  |  |  |  |  |  |  |
|  | rs1810126 | 0.981 | 4 | *SLC22A3* | | 2.90E-08 | | Heart - Left Ventricle | | | | | | | | |  |  |  |  |  |  |  |
|  |  |  |  | *SLC22A3* | | 7.70E-13 | | Artery - Tibial | | | | | | | | |  |  |  |  |  |  |  |
|  | rs3088442 | 0.987 | 6 | *SLC22A3* | | 2.40E-08 | | Heart - Left Ventricle | | | | | | | | |  |  |  |  |  |  |  |
|  |  |  |  | *SLC22A3* | | 4.70E-13 | | Artery - Tibial | | | | | | | | |  |  |  |  |  |  |  |
|  | rs388170 | 0.931 | 4 | *SLC22A3* | | 3.90E-09 | | Heart - Left Ventricle | | | | | | | | |  |  |  |  |  |  |  |
|  |  |  |  | *SLC22A3* | | 3.00E-12 | | Artery - Tibial | | | | | | | | |  |  |  |  |  |  |  |
|  | rs3106164 | 0.953 | 5 | *SLC22A3* | | 3.70E-09 | | Heart - Left Ventricle | | | | | | | | |  |  |  |  |  |  |  |
|  |  |  |  | *SLC22A3* | | 2.20E-12 | | Artery - Tibial | | | | | | | | |  |  |  |  |  |  |  |
|  | rs2063347 | 0.987 | No data | *SLC22A3* | | 1.20E-07 | | Heart - Left Ventricle | | | | | | | | |  |  |  |  |  |  |  |
|  |  |  |  | *SLC22A3* | | 2.10E-12 | | Artery - Tibial | | | | | | | | |  |  |  |  |  |  |  |
|  | rs9355288 | 0.871 | No data | *SLC22A3* | | 2.40E-08 | | Heart - Left Ventricle | | | | | | | | |  |  |  |  |  |  |  |
|  |  |  |  | *SLC22A3* | | 4.70E-13 | | Artery - Tibial | | | | | | | | |  |  |  |  |  |  |  |
|  | rs2292334 | 0.881 | No data | *SLC22A3* | | 3.20E-07 | | Heart - Left Ventricle | | | | | | | | |  |  |  |  |  |  |  |
|  |  |  |  | *SLC22A3* | | 2.90E-12 | | Artery - Tibial | | | | | | | | |  |  |  |  |  |  |  |
|  | rs7769879 | 0.883 | No data | *SLC22A3* | | 2.40E-08 | | Heart - Left Ventricle | | | | | | | | |  |  |  |  |  |  |  |
|  |  |  |  | *SLC22A3* | | 7.50E-13 | | Artery - Tibial | | | | | | | | |  |  |  |  |  |  |  |
|  | rs3918285 | 0.801 | No data | *SLC22A3* | | 2.40E-08 | | Heart - Left Ventricle | | | | | | | | |  |  |  |  |  |  |  |
|  |  |  |  | *SLC22A3* | | 4.70E-13 | | Artery - Tibial | | | | | | | | |  |  |  |  |  |  |  |
|  | rs3918286 | 0.819 | No data | *SLC22A3* | | 2.40E-08 | | Heart - Left Ventricle | | | | | | | | |  |  |  |  |  |  |  |
|  |  |  |  | *SLC22A3* | | 4.70E-13 | | Artery - Tibial | | | | | | | | |  |  |  |  |  |  |  |
|  | rs1810126 | 0.843 | 4 | *SLC22A3* | | 2.90E-08 | | Heart - Left Ventricle | | | | | | | | |  |  |  |  |  |  |  |
|  |  |  |  | *SLC22A3* | | 7.70E-13 | | Artery - Tibial | | | | | | | | |  |  |  |  |  |  |  |
|  | rs3088442 | 0.959 | 6 | *SLC22A3* | | 2.40E-08 | | Heart - Left Ventricle | | | | | | | | |  |  |  |  |  |  |  |
|  |  |  |  | *SLC22A3* | | 4.70E-13 | | Artery - Tibial | | | | | | | | |  |  |  |  |  |  |  |
| ***SORT1*/rs602633** |  |  |  |  | |  |  | | | | | | | | |  |  |  |  |  |  |  |  |
|  | rs602633 | 1 | 7 | *PSRC1* | | 5.9E-15 | Whole blood | | | | | | | | |  |  |  |  |  |  |  |  |
|  |  |  |  | *SYPL2* | | 0.000005 | Artery aorta | | | | | | | | |  |  |  |  |  |  |  |  |
|  |  |  |  | *PSRC1* | | 0.000017 | Heart - Left Ventricle | | | | | | | | |  |  |  |  |  |  |  |  |
|  | rs660240 | 0.94 | 4 | *PSRC1* | | 5.9E-18 | Whole blood | | | | | | | | |  |  |  |  |  |  |  |  |
|  |  |  |  | *SYPL2* | | 0.00005 | Artery aorta | | | | | | | | |  |  |  |  |  |  |  |  |
|  |  |  |  | *PSRC1* | | 0.00000009 | Heart - Left Ventricle | | | | | | | | |  |  |  |  |  |  |  |  |
|  | rs1277930 | 0.9152 | 6 | *PSRC1* | | 2.90E-15 | Whole Blood | | | | | | | | |  |  |  |  |  |  |  |  |
|  |  |  |  | *SYPL2* | | 0.000066 | Artery aorta | | | | | | | | |  |  |  |  |  |  |  |  |
|  |  |  |  | *PSRC1* | | 0.000017 | Heart - Left Ventricle | | | | | | | | |  |  |  |  |  |  |  |  |
|  | rs599839 | 0.9152 | 6 | *PSRC1* | | 2.90E-15 | Whole Blood | | | | | | | | |  |  |  |  |  |  |  |  |
|  |  |  |  | *SYPL2* | | 0.000066 | Artery aorta | | | | | | | | |  |  |  |  |  |  |  |  |
|  |  |  |  | *PSRC1* | | 0.000017 | Heart - Left Ventricle | | | | | | | | |  |  |  |  |  |  |  |  |
|  | rs4970836 | 0.9138 | 7 | *No data* | |  |  | | | | | | | | |  |  |  |  |  |  |  |  |
|  |  |  |  |  | |  |  | | | | | | | | |  |  |  |  |  |  |  |  |
|  |  |  |  |  | |  |  | | | | | | | | |  |  |  |  |  |  |  |  |
|  | rs583104 | 0.91 | 7 | *PSRC1* | | 3.10E-15 | Whole Blood | | | | | | | | |  |  |  |  |  |  |  |  |
|  |  |  |  | *SYPL2* | | 0.000041 | Artery aorta | | | | | | | | |  |  |  |  |  |  |  |  |
|  |  |  |  | *PSRC1* | | 0.000017 | Heart - Left Ventricle | | | | | | | | |  |  |  |  |  |  |  |  |
|  | **rs646776** | **0.8615** | **1f** | ***PSRC1*** | | **2.30E-18** | **Whole Blood** | | | | | | | | |  |  |  |  |  |  |  |  |
|  |  |  |  | ***SYPL2*** | | **1.00E-07** | **Heart - Left Ventricle** | | | | | | | | |  |  |  |  |  |  |  |  |
|  | **rs12740374** | **0.8615** | **2b** | ***PSRC1*** | | **4.00E-16** | **Whole Blood** | | | | | | | | |  |  |  |  |  |  |  |  |
|  |  |  |  | *SYPL2* | | 4.50E-07 | Heart - Left Ventricle | | | | | | | | |  |  |  |  |  |  |  |  |
|  | rs7528419 | 0.8615 | 3a | *PSRC1* | | 1.10E-15 | Whole Blood | | | | | | | | |  |  |  |  |  |  |  |  |
|  |  |  |  | *PSRC1* | | 4.10E-07 | Heart - Left Ventricle | | | | | | | | |  |  |  |  |  |  |  |  |
|  | **rs629301** | **0.8563** | **1f** | ***PSRC1*** | | **2.30E-18** | **Whole Blood** | | | | | | | | |  |  |  |  |  |  |  |  |
|  |  |  |  | *PSRC1* | | 1.00E-07 | Heart - Left Ventricle | | | | | | | | |  |  |  |  |  |  |  |  |
| ***UBE2Z*/rs46522** |  |  |  |  | |  |  | | | | | | | | |  |  |  |  |  |  |  |  |
|  | **rs46522** | **1** | **1f** | ***UBE2Z*** | | **1.50E-44** | **Whole Blood** | | | | | | | | |  |  |  |  |  |  |  |  |
|  |  |  |  | ***SNF8*** | | **9.40E-08** | **Artery Aorta** | | | | | | | | |  |  |  |  |  |  |  |  |
|  |  |  |  | [***SUMO2P17***](javascript:EqtlsPage.methods.gotoGeneExpression('ENSG00000248278.1')) | | **5.20E-09** | **Artery Tibial** | | | | | | | | |  |  |  |  |  |  |  |  |
|  |  |  |  | [***ATP5G1***](javascript:EqtlsPage.methods.gotoGeneExpression('ENSG00000159199.13')) | | **2.30E-15** | [**Heart - Left Ventricle**](javascript:EqtlsPage.methods.goTissuePage('Heart_Left_Ventricle')) | | | | | | | | |  |  |  |  |  |  |  |  |
|  | rs46521 | 1 | 5 | *UBE2Z* | | 1.50E-44 | Whole Blood | | | | | | | | |  |  |  |  |  |  |  |  |
|  |  |  |  | *SNF8* | | 9.40E-08 | Artery Aorta | | | | | | | | |  |  |  |  |  |  |  |  |
|  |  |  |  | [*SUMO2P17*](javascript:EqtlsPage.methods.gotoGeneExpression('ENSG00000248278.1')) | | 5.20E-09 | Artery Tibial | | | | | | | | |  |  |  |  |  |  |  |  |
|  |  |  |  | [*ATP5G1*](javascript:EqtlsPage.methods.gotoGeneExpression('ENSG00000159199.13')) | | 2.30E-15 | [Heart - Left Ventricle](javascript:EqtlsPage.methods.goTissuePage('Heart_Left_Ventricle')) | | | | | | | | |  |  |  |  |  |  |  |  |
|  | rs318093 | 1 | 6 | *UBE2Z* | | 1.50E-44 | Whole Blood | | | | | | | | |  |  |  |  |  |  |  |  |
|  |  |  |  | *SNF8* | | 9.40E-08 | Artery Aorta | | | | | | | | |  |  |  |  |  |  |  |  |
|  |  |  |  | [*SUMO2P17*](javascript:EqtlsPage.methods.gotoGeneExpression('ENSG00000248278.1')) | | 5.20E-09 | Artery Tibial | | | | | | | | |  |  |  |  |  |  |  |  |
|  |  |  |  | [*ATP5G1*](javascript:EqtlsPage.methods.gotoGeneExpression('ENSG00000159199.13')) | | 2.30E-15 | [Heart - Left Ventricle](javascript:EqtlsPage.methods.goTissuePage('Heart_Left_Ventricle')) | | | | | | | | |  |  |  |  |  |  |  |  |
|  | rs62075824 | 1 | 4 | *UBE2Z* | | 1.40E-44 | Whole Blood | | | | | | | | |  |  |  |  |  |  |  |  |
|  |  |  |  | *SNF8* | | 1.00E-08 | Artery Aorta | | | | | | | | |  |  |  |  |  |  |  |  |
|  |  |  |  | [*SUMO2P17*](javascript:EqtlsPage.methods.gotoGeneExpression('ENSG00000248278.1')) | | 7.50E-09 | Artery Tibial | | | | | | | | |  |  |  |  |  |  |  |  |
|  |  |  |  | [*ATP5G1*](javascript:EqtlsPage.methods.gotoGeneExpression('ENSG00000159199.13')) | | 2.10E-15 | [Heart - Left Ventricle](javascript:EqtlsPage.methods.goTissuePage('Heart_Left_Ventricle')) | | | | | | | | |  |  |  |  |  |  |  |  |
|  | rs318092 | 1 | 7 | *UBE2Z* | | 1.50E-44 | Whole Blood | | | | | | | | |  |  |  |  |  |  |  |  |
|  |  |  |  | *SNF8* | | 9.40E-08 | Artery Aorta | | | | | | | | |  |  |  |  |  |  |  |  |
|  |  |  |  | [*SUMO2P17*](javascript:EqtlsPage.methods.gotoGeneExpression('ENSG00000248278.1')) | | 5.20E-09 | Artery Tibial | | | | | | | | |  |  |  |  |  |  |  |  |
|  |  |  |  | [*ATP5G1*](javascript:EqtlsPage.methods.gotoGeneExpression('ENSG00000159199.13')) | | 2.30E-15 | [Heart - Left Ventricle](javascript:EqtlsPage.methods.goTissuePage('Heart_Left_Ventricle')) | | | | | | | | |  |  |  |  |  |  |  |  |
|  | rs170319 | 1 | 7 | *UBE2Z* | | 1.50E-44 | Whole Blood | | | | | | | | |  |  |  |  |  |  |  |  |
|  |  |  |  | *SNF8* | | 9.40E-08 | Artery Aorta | | | | | | | | |  |  |  |  |  |  |  |  |
|  |  |  |  | [*SUMO2P17*](javascript:EqtlsPage.methods.gotoGeneExpression('ENSG00000248278.1')) | | 5.20E-09 | Artery Tibial | | | | | | | | |  |  |  |  |  |  |  |  |
|  |  |  |  | [*ATP5G1*](javascript:EqtlsPage.methods.gotoGeneExpression('ENSG00000159199.13')) | | 2.30E-15 | [Heart - Left Ventricle](file:///E:\..\..\javascript\EqtlsPage.methods.goTissuePage('Heart_Left_Ventricle')) | | | | | | | | |  |  |  |  |  |  |  |  |
|  | **rs61576918** | **1** | **2c** | ***UBE2Z*** | | **1.50E-44** | **Whole Blood** | | | | | | | | |  |  |  |  |  |  |  |  |
|  |  |  |  | *SNF8* | | 9.40E-08 | Artery Aorta | | | | | | | | |  |  |  |  |  |  |  |  |
|  |  |  |  | [*SUMO2P17*](javascript:EqtlsPage.methods.gotoGeneExpression('ENSG00000248278.1')) | | 5.20E-09 | Artery Tibial | | | | | | | | |  |  |  |  |  |  |  |  |
|  |  |  |  | [*ATP5G1*](javascript:EqtlsPage.methods.gotoGeneExpression('ENSG00000159199.13')) | | 2.30E-15 | [Heart - Left Ventricle](file:///E:\..\..\javascript\EqtlsPage.methods.goTissuePage('Heart_Left_Ventricle')) | | | | | | | | |  |  |  |  |  |  |  |  |
|  | rs318090 | 1 | 6 | *UBE2Z* | | 1.50E-44 | Whole Blood | | | | | | | | |  |  |  |  |  |  |  |  |
|  |  |  |  | *SNF8* | | 9.40E-08 | Artery Aorta | | | | | | | | |  |  |  |  |  |  |  |  |
|  |  |  |  | [*SUMO2P17*](javascript:EqtlsPage.methods.gotoGeneExpression('ENSG00000248278.1')) | | 5.20E-09 | Artery Tibial | | | | | | | | |  |  |  |  |  |  |  |  |
|  |  |  |  | [*ATP5G1*](javascript:EqtlsPage.methods.gotoGeneExpression('ENSG00000159199.13')) | | 2.30E-15 | [Heart - Left Ventricle](file:///E:\..\..\javascript\EqtlsPage.methods.goTissuePage('Heart_Left_Ventricle')) | | | | | | | | |  |  |  |  |  |  |  |  |
|  | rs55819356 | 1 | 6 | *UBE2Z* | | 7.30E-45 | Whole Blood | | | | | | | | |  |  |  |  |  |  |  |  |
|  |  |  |  | *SNF8* | | 2.70E-07 | Artery Aorta | | | | | | | | |  |  |  |  |  |  |  |  |
|  |  |  |  | [*SUMO2P17*](javascript:EqtlsPage.methods.gotoGeneExpression('ENSG00000248278.1')) | | 9.70E-09 | Artery Tibial | | | | | | | | |  |  |  |  |  |  |  |  |
|  |  |  |  | [*ATP5G1*](javascript:EqtlsPage.methods.gotoGeneExpression('ENSG00000159199.13')) | | 5.60E-15 | [Heart - Left Ventricle](file:///E:\..\..\javascript\EqtlsPage.methods.goTissuePage('Heart_Left_Ventricle')) | | | | | | | | |  |  |  |  |  |  |  |  |
|  | rs56236620 | 1 | 7 |  | | No data |  | | | | | | | | |  |  |  |  |  |  |  |  |
|  | rs34899772 | 1 | 6 | *UBE2Z* | | 7.30E-45 | Whole Blood | | | | | | | | |  |  |  |  |  |  |  |  |
|  |  |  |  | *SNF8* | | 2.70E-07 | Artery Aorta | | | | | | | | |  |  |  |  |  |  |  |  |
|  |  |  |  | [*SUMO2P17*](javascript:EqtlsPage.methods.gotoGeneExpression('ENSG00000248278.1')) | | 9.70E-09 | Artery Tibial | | | | | | | | |  |  |  |  |  |  |  |  |
|  |  |  |  | [*ATP5G1*](javascript:EqtlsPage.methods.gotoGeneExpression('ENSG00000159199.13')) | | 5.60E-15 | [Heart - Left Ventricle](file:///E:\..\..\javascript\EqtlsPage.methods.goTissuePage('Heart_Left_Ventricle')) | | | | | | | | |  |  |  |  |  |  |  |  |
|  | rs9902131 | 1 | 7 | *UBE2Z* | | 7.30E-45 | Whole Blood | | | | | | | | |  |  |  |  |  |  |  |  |
|  |  |  |  | *SNF8* | | 2.70E-07 | Artery Aorta | | | | | | | | |  |  |  |  |  |  |  |  |
|  |  |  |  | [*SUMO2P17*](javascript:EqtlsPage.methods.gotoGeneExpression('ENSG00000248278.1')) | | 9.70E-09 | Artery Tibial | | | | | | | | |  |  |  |  |  |  |  |  |
|  |  |  |  | [*ATP5G1*](javascript:EqtlsPage.methods.gotoGeneExpression('ENSG00000159199.13')) | | 5.60E-15 | [Heart - Left Ventricle](file:///E:\..\..\javascript\EqtlsPage.methods.goTissuePage('Heart_Left_Ventricle')) | | | | | | | | |  |  |  |  |  |  |  |  |
|  | **rs3744608** | **1** | **2a** |  | | No data |  | | | | | | | | |  |  |  |  |  |  |  |  |
|  | rs4378658 | 1 | 4 | *UBE2Z* | | 1.60E-43 | Whole Blood | | | | | | | | |  |  |  |  |  |  |  |  |
|  |  |  |  | *SNF8* | | 1.20E-07 | Artery Aorta | | | | | | | | |  |  |  |  |  |  |  |  |
|  |  |  |  | [*SUMO2P17*](javascript:EqtlsPage.methods.gotoGeneExpression('ENSG00000248278.1')) | | 6.70E-09 | Artery Tibial | | | | | | | | |  |  |  |  |  |  |  |  |
|  |  |  |  | [*ATP5G1*](javascript:EqtlsPage.methods.gotoGeneExpression('ENSG00000159199.13')) | | 1.60E-15 | [Heart - Left Ventricle](file:///E:\..\..\javascript\EqtlsPage.methods.goTissuePage('Heart_Left_Ventricle')) | | | | | | | | |  |  |  |  |  |  |  |  |
|  | rs4597361 | 1 | 5 | *UBE2Z* | | 1.50E-44 | Whole Blood | | | | | | | | |  |  |  |  |  |  |  |  |
|  |  |  |  | *SNF8* | | 1.20E-07 | Artery Aorta | | | | | | | | |  |  |  |  |  |  |  |  |
|  |  |  |  | [*SUMO2P17*](javascript:EqtlsPage.methods.gotoGeneExpression('ENSG00000248278.1')) | | 6.90E-09 | Artery Tibial | | | | | | | | |  |  |  |  |  |  |  |  |
|  |  |  |  | [*ATP5G1*](javascript:EqtlsPage.methods.gotoGeneExpression('ENSG00000159199.13')) | | 3.10E-15 | [Heart - Left Ventricle](file:///E:\..\..\javascript\EqtlsPage.methods.goTissuePage('Heart_Left_Ventricle')) | | | | | | | | |  |  |  |  |  |  |  |  |
|  | rs4294857 | 1 | 5 | *UBE2Z* | | 7.30E-45 | Whole Blood | | | | | | | | |  |  |  |  |  |  |  |  |
|  |  |  |  | *SNF8* | | 2.70E-07 | Artery Aorta | | | | | | | | |  |  |  |  |  |  |  |  |
|  |  |  |  | [*SUMO2P17*](javascript:EqtlsPage.methods.gotoGeneExpression('ENSG00000248278.1')) | | 9.70E-09 | Artery Tibial | | | | | | | | |  |  |  |  |  |  |  |  |
|  |  |  |  | [*ATP5G1*](javascript:EqtlsPage.methods.gotoGeneExpression('ENSG00000159199.13')) | | 5.60E-15 | [Heart - Left Ventricle](file:///E:\..\..\javascript\EqtlsPage.methods.goTissuePage('Heart_Left_Ventricle')) | | | | | | | | |  |  |  |  |  |  |  |  |
|  | rs62075820 | 1 | 5 | *UBE2Z* | | 7.30E-45 | Whole Blood | | | | | | | | |  |  |  |  |  |  |  |  |
|  |  |  |  | *SNF8* | | 2.70E-07 | Artery Aorta | | | | | | | | |  |  |  |  |  |  |  |  |
|  |  |  |  | [*SUMO2P17*](javascript:EqtlsPage.methods.gotoGeneExpression('ENSG00000248278.1')) | | 9.70E-09 | Artery Tibial | | | | | | | | |  |  |  |  |  |  |  |  |
|  |  |  |  | [*ATP5G1*](javascript:EqtlsPage.methods.gotoGeneExpression('ENSG00000159199.13')) | | 5.60E-15 | [Heart - Left Ventricle](file:///E:\..\..\javascript\EqtlsPage.methods.goTissuePage('Heart_Left_Ventricle')) | | | | | | | | |  |  |  |  |  |  |  |  |
|  | rs12603261 | 1 | 7 | *UBE2Z* | | 1.50E-44 | Whole Blood | | | | | | | | |  |  |  |  |  |  |  |  |
|  |  |  |  | *SNF8* | | 9.40E-08 | Artery Aorta | | | | | | | | |  |  |  |  |  |  |  |  |
|  |  |  |  | [*SUMO2P17*](javascript:EqtlsPage.methods.gotoGeneExpression('ENSG00000248278.1')) | | 5.20E-09 | Artery Tibial | | | | | | | | |  |  |  |  |  |  |  |  |
|  |  |  |  | [*ATP5G1*](javascript:EqtlsPage.methods.gotoGeneExpression('ENSG00000159199.13')) | | 2.30E-15 | [Heart - Left Ventricle](file:///E:\..\..\javascript\EqtlsPage.methods.goTissuePage('Heart_Left_Ventricle')) | | | | | | | | |  |  |  |  |  |  |  |  |
|  | rs9912829 | 1 | 6 | *UBE2Z* | | 7.30E-45 | Whole Blood | | | | | | | | |  |  |  |  |  |  |  |  |
|  |  |  |  | *SNF8* | | 2.70E-07 | Artery Aorta | | | | | | | | |  |  |  |  |  |  |  |  |
|  |  |  |  | [*SUMO2P17*](javascript:EqtlsPage.methods.gotoGeneExpression('ENSG00000248278.1')) | | 9.70E-09 | Artery Tibial | | | | | | | | |  |  |  |  |  |  |  |  |
|  |  |  |  | [*ATP5G1*](javascript:EqtlsPage.methods.gotoGeneExpression('ENSG00000159199.13')) | | 5.60E-15 | [Heart - Left Ventricle](file:///E:\..\..\javascript\EqtlsPage.methods.goTissuePage('Heart_Left_Ventricle')) | | | | | | | | |  |  |  |  |  |  |  |  |
|  | rs12453374 | 1 | 5 | *UBE2Z* | | 1.50E-44 | Whole Blood | | | | | | | | |  |  |  |  |  |  |  |  |
|  |  |  |  | *SNF8* | | 9.40E-08 | Artery Aorta | | | | | | | | |  |  |  |  |  |  |  |  |
|  |  |  |  | [*SUMO2P17*](javascript:EqtlsPage.methods.gotoGeneExpression('ENSG00000248278.1')) | | 5.20E-09 | Artery Tibial | | | | | | | | |  |  |  |  |  |  |  |  |
|  |  |  |  | [*ATP5G1*](javascript:EqtlsPage.methods.gotoGeneExpression('ENSG00000159199.13')) | | 2.30E-15 | [Heart - Left Ventricle](file:///E:\..\..\javascript\EqtlsPage.methods.goTissuePage('Heart_Left_Ventricle')) | | | | | | | | |  |  |  |  |  |  |  |  |
|  | rs62075818 | 1 | 6 | *UBE2Z* | | 7.30E-45 | Whole Blood | | | | | | | | |  |  |  |  |  |  |  |  |
|  |  |  |  | *SNF8* | | 2.70E-07 | Artery Aorta | | | | | | | | |  |  |  |  |  |  |  |  |
|  |  |  |  | [*SUMO2P17*](javascript:EqtlsPage.methods.gotoGeneExpression('ENSG00000248278.1')) | | 9.70E-09 | Artery Tibial | | | | | | | | |  |  |  |  |  |  |  |  |
|  |  |  |  | [*ATP5G1*](javascript:EqtlsPage.methods.gotoGeneExpression('ENSG00000159199.13')) | | 2.30E-15 | [Heart - Left Ventricle](file:///E:\..\..\javascript\EqtlsPage.methods.goTissuePage('Heart_Left_Ventricle')) | | | | | | | | |  |  |  |  |  |  |  |  |
|  | rs12453394 | 1 | 7 | *UBE2Z* | | 1.50E-44 | Whole Blood | | | | | | | | |  |  |  |  |  |  |  |  |
|  |  |  |  | *SNF8* | | 9.40E-08 | Artery Aorta | | | | | | | | |  |  |  |  |  |  |  |  |
|  |  |  |  | [*SUMO2P17*](javascript:EqtlsPage.methods.gotoGeneExpression('ENSG00000248278.1')) | | 5.20E-09 | Artery Tibial | | | | | | | | |  |  |  |  |  |  |  |  |
|  |  |  |  | [*ATP5G1*](javascript:EqtlsPage.methods.gotoGeneExpression('ENSG00000159199.13')) | | 2.30E-15 | [Heart - Left Ventricle](file:///E:\..\..\javascript\EqtlsPage.methods.goTissuePage('Heart_Left_Ventricle')) | | | | | | | | |  |  |  |  |  |  |  |  |
|  | rs12451879 | 1 | 7 | *UBE2Z* | | 1.50E-44 | Whole Blood | | | | | | | | |  |  |  |  |  |  |  |  |
|  |  |  |  | *SNF8* | | 9.40E-08 | Artery Aorta | | | | | | | | |  |  |  |  |  |  |  |  |
|  |  |  |  | [*SUMO2P17*](javascript:EqtlsPage.methods.gotoGeneExpression('ENSG00000248278.1')) | | 5.20E-09 | Artery Tibial | | | | | | | | |  |  |  |  |  |  |  |  |
|  |  |  |  | [*ATP5G1*](javascript:EqtlsPage.methods.gotoGeneExpression('ENSG00000159199.13')) | | 5.60E-15 | [Heart - Left Ventricle](file:///E:\..\..\javascript\EqtlsPage.methods.goTissuePage('Heart_Left_Ventricle')) | | | | | | | | |  |  |  |  |  |  |  |  |
|  | rs903567 | 1 | 7 | *UBE2Z* | | 7.30E-45 | Whole Blood | | | | | | | | |  |  |  |  |  |  |  |  |
|  |  |  |  | *SNF8* | | 2.70E-07 | Artery Aorta | | | | | | | | |  |  |  |  |  |  |  |  |
|  |  |  |  | [*SUMO2P17*](javascript:EqtlsPage.methods.gotoGeneExpression('ENSG00000248278.1')) | | 9.70E-09 | Artery Tibial | | | | | | | | |  |  |  |  |  |  |  |  |
|  |  |  |  | [*ATP5G1*](javascript:EqtlsPage.methods.gotoGeneExpression('ENSG00000159199.13')) | | 2.30E-15 | [Heart - Left Ventricle](file:///E:\..\..\javascript\EqtlsPage.methods.goTissuePage('Heart_Left_Ventricle')) | | | | | | | | |  |  |  |  |  |  |  |  |
|  | rs12601072 | 1 | 7 | *UBE2Z* | | 1.50E-44 | Whole Blood | | | | | | | | |  |  |  |  |  |  |  |  |
|  |  |  |  | *SNF8* | | 9.40E-08 | Artery Aorta | | | | | | | | |  |  |  |  |  |  |  |  |
|  |  |  |  | [*SUMO2P17*](javascript:EqtlsPage.methods.gotoGeneExpression('ENSG00000248278.1')) | | 5.20E-09 | Artery Tibial | | | | | | | | |  |  |  |  |  |  |  |  |
|  |  |  |  | [*ATP5G1*](javascript:EqtlsPage.methods.gotoGeneExpression('ENSG00000159199.13')) | | 2.00E-15 | [Heart - Left Ventricle](file:///E:\..\..\javascript\EqtlsPage.methods.goTissuePage('Heart_Left_Ventricle')) | | | | | | | | |  |  |  |  |  |  |  |  |
|  | rs28409394 | 1 | 7 | *UBE2Z* | | 1.70E-43 | Whole Blood | | | | | | | | |  |  |  |  |  |  |  |  |
|  |  |  |  | *SNF8* | | 3.30E-08 | Artery Aorta | | | | | | | | |  |  |  |  |  |  |  |  |
|  |  |  |  | [*SUMO2P17*](javascript:EqtlsPage.methods.gotoGeneExpression('ENSG00000248278.1')) | | 2.60E-09 | Artery Tibial | | | | | | | | |  |  |  |  |  |  |  |  |
|  |  |  |  | [*ATP5G1*](javascript:EqtlsPage.methods.gotoGeneExpression('ENSG00000159199.13')) | | 2.30E-15 | [Heart - Left Ventricle](file:///E:\..\..\javascript\EqtlsPage.methods.goTissuePage('Heart_Left_Ventricle')) | | | | | | | | |  |  |  |  |  |  |  |  |
|  | rs58838744 | 1 | 5 | *UBE2Z* | | 1.50E-44 | Whole Blood | | | | | | | | |  |  |  |  |  |  |  |  |
|  |  |  |  | *SNF8* | | 9.40E-08 | Artery Aorta | | | | | | | | |  |  |  |  |  |  |  |  |
|  |  |  |  | [*SUMO2P17*](javascript:EqtlsPage.methods.gotoGeneExpression('ENSG00000248278.1')) | | 5.20E-09 | Artery Tibial | | | | | | | | |  |  |  |  |  |  |  |  |
|  |  |  |  | [*ATP5G1*](javascript:EqtlsPage.methods.gotoGeneExpression('ENSG00000159199.13')) | | 5.60E-15 | [Heart - Left Ventricle](file:///E:\..\..\javascript\EqtlsPage.methods.goTissuePage('Heart_Left_Ventricle')) | | | | | | | | |  |  |  |  |  |  |  |  |
|  | rs962272 | 1 | 7 | *UBE2Z* | | 7.30E-45 | Whole Blood | | | | | | | | |  |  |  |  |  |  |  |  |
|  |  |  |  | *SNF8* | | 2.70E-07 | Artery Aorta | | | | | | | | |  |  |  |  |  |  |  |  |
|  |  |  |  | [*SUMO2P17*](javascript:EqtlsPage.methods.gotoGeneExpression('ENSG00000248278.1')) | | 9.70E-09 | Artery Tibial | | | | | | | | |  |  |  |  |  |  |  |  |
|  |  |  |  | [*ATP5G1*](javascript:EqtlsPage.methods.gotoGeneExpression('ENSG00000159199.13')) | | 5.60E-15 | [Heart - Left Ventricle](file:///E:\..\..\javascript\EqtlsPage.methods.goTissuePage('Heart_Left_Ventricle')) | | | | | | | | |  |  |  |  |  |  |  |  |
|  | rs2546491 | 1 | 3a | *UBE2Z* | | 7.30E-45 | Whole Blood | | | | | | | | |  |  |  |  |  |  |  |  |
|  |  |  |  | *SNF8* | | 2.70E-07 | Artery Aorta | | | | | | | | |  |  |  |  |  |  |  |  |
|  |  |  |  | [*SUMO2P17*](javascript:EqtlsPage.methods.gotoGeneExpression('ENSG00000248278.1')) | | 6.60E-09 | Artery Tibial | | | | | | | | |  |  |  |  |  |  |  |  |
|  |  |  |  | [*ATP5G1*](javascript:EqtlsPage.methods.gotoGeneExpression('ENSG00000159199.13')) | | 2.30E-15 | [Heart - Left Ventricle](file:///E:\..\..\javascript\EqtlsPage.methods.goTissuePage('Heart_Left_Ventricle')) | | | | | | | | |  |  |  |  |  |  |  |  |
|  | rs60708039 | 1 | 7 | *UBE2Z* | | 1.50E-44 | Whole Blood | | | | | | | | |  |  |  |  |  |  |  |  |
|  |  |  |  | *SNF8* | | 9.40E-08 | Artery Aorta | | | | | | | | |  |  |  |  |  |  |  |  |
|  |  |  |  | [*SUMO2P17*](javascript:EqtlsPage.methods.gotoGeneExpression('ENSG00000248278.1')) | | 5.20E-09 | Artery Tibial | | | | | | | | |  |  |  |  |  |  |  |  |
|  |  |  |  | [*ATP5G1*](javascript:EqtlsPage.methods.gotoGeneExpression('ENSG00000159199.13')) | | 4.10E-15 | [Heart - Left Ventricle](file:///E:\..\..\javascript\EqtlsPage.methods.goTissuePage('Heart_Left_Ventricle')) | | | | | | | | |  |  |  |  |  |  |  |  |
|  | rs60540186 | 1 | 7 | *UBE2Z* | | 3.00E-44 | Whole Blood | | | | | | | | |  |  |  |  |  |  |  |  |
|  |  |  |  | *SNF8* | | 4.80E-08 | Artery Aorta | | | | | | | | |  |  |  |  |  |  |  |  |
|  |  |  |  | [*SUMO2P17*](javascript:EqtlsPage.methods.gotoGeneExpression('ENSG00000248278.1')) | | 3.30E-09 | Artery Tibial | | | | | | | | |  |  |  |  |  |  |  |  |
|  |  |  |  | [*ATP5G1*](javascript:EqtlsPage.methods.gotoGeneExpression('ENSG00000159199.13')) | | 5.60E-15 | [Heart - Left Ventricle](file:///E:\..\..\javascript\EqtlsPage.methods.goTissuePage('Heart_Left_Ventricle')) | | | | | | | | |  |  |  |  |  |  |  |  |
|  | rs2643361 | 1 | 3a | *UBE2Z* | | 7.30E-45 | Whole Blood | | | | | | | | |  |  |  |  |  |  |  |  |
|  |  |  |  | *SNF8* | | 2.70E-07 | Artery Aorta | | | | | | | | |  |  |  |  |  |  |  |  |
|  |  |  |  | [*SUMO2P17*](javascript:EqtlsPage.methods.gotoGeneExpression('ENSG00000248278.1')) | | 1.10E-08 | Artery Tibial | | | | | | | | |  |  |  |  |  |  |  |  |
|  |  |  |  | [*ATP5G1*](javascript:EqtlsPage.methods.gotoGeneExpression('ENSG00000159199.13')) | | 2.30E-15 | [Heart - Left Ventricle](file:///E:\..\..\javascript\EqtlsPage.methods.goTissuePage('Heart_Left_Ventricle')) | | | | | | | | |  |  |  |  |  |  |  |  |
|  | rs55771415 | 1 | 7 | *UBE2Z* | | 1.50E-44 | Whole Blood | | | | | | | | |  |  |  |  |  |  |  |  |
|  |  |  |  | *SNF8* | | 9.40E-08 | Artery Aorta | | | | | | | | |  |  |  |  |  |  |  |  |
|  |  |  |  | [*SUMO2P17*](javascript:EqtlsPage.methods.gotoGeneExpression('ENSG00000248278.1')) | | 5.20E-09 | Artery Tibial | | | | | | | | |  |  |  |  |  |  |  |  |
|  |  |  |  | [*ATP5G1*](javascript:EqtlsPage.methods.gotoGeneExpression('ENSG00000159199.13')) | | 2.30E-15 | [Heart - Left Ventricle](file:///E:\..\..\javascript\EqtlsPage.methods.goTissuePage('Heart_Left_Ventricle')) | | | | | | | | |  |  |  |  |  |  |  |  |
|  | rs2088139 | 1 | 7 | *UBE2Z* | | 1.50E-44 | Whole Blood | | | | | | | | |  |  |  |  |  |  |  |  |
|  |  |  |  | *SNF8* | | 9.40E-08 | Artery Aorta | | | | | | | | |  |  |  |  |  |  |  |  |
|  |  |  |  | [*SUMO2P17*](javascript:EqtlsPage.methods.gotoGeneExpression('ENSG00000248278.1')) | | 5.20E-09 | Artery Tibial | | | | | | | | |  |  |  |  |  |  |  |  |
|  |  |  |  | [*ATP5G1*](javascript:EqtlsPage.methods.gotoGeneExpression('ENSG00000159199.13')) | | 5.60E-15 | [Heart - Left Ventricle](file:///E:\..\..\javascript\EqtlsPage.methods.goTissuePage('Heart_Left_Ventricle')) | | | | | | | | |  |  |  |  |  |  |  |  |
|  | rs28694768 | 1 | 6 | *UBE2Z* | | 7.30E-45 | Whole Blood | | | | | | | | |  |  |  |  |  |  |  |  |
|  |  |  |  | *SNF8* | | 2.70E-07 | Artery Aorta | | | | | | | | |  |  |  |  |  |  |  |  |
|  |  |  |  | [*SUMO2P17*](javascript:EqtlsPage.methods.gotoGeneExpression('ENSG00000248278.1')) | | 6.60E-09 | Artery Tibial | | | | | | | | |  |  |  |  |  |  |  |  |
|  |  |  |  | [*ATP5G1*](javascript:EqtlsPage.methods.gotoGeneExpression('ENSG00000159199.13')) | | 5.60E-15 | [Heart - Left Ventricle](file:///E:\..\..\javascript\EqtlsPage.methods.goTissuePage('Heart_Left_Ventricle')) | | | | | | | | |  |  |  |  |  |  |  |  |
|  | rs28517720 | 1 | 7 | *UBE2Z* | | 7.30E-45 | Whole Blood | | | | | | | | |  |  |  |  |  |  |  |  |
|  |  |  |  | *SNF8* | | 2.70E-07 | Artery Aorta | | | | | | | | |  |  |  |  |  |  |  |  |
|  |  |  |  | [*SUMO2P17*](javascript:EqtlsPage.methods.gotoGeneExpression('ENSG00000248278.1')) | | 6.60E-09 | Artery Tibial | | | | | | | | |  |  |  |  |  |  |  |  |
|  |  |  |  | [*ATP5G1*](javascript:EqtlsPage.methods.gotoGeneExpression('ENSG00000159199.13')) | | 5.60E-15 | [Heart - Left Ventricle](file:///E:\..\..\javascript\EqtlsPage.methods.goTissuePage('Heart_Left_Ventricle')) | | | | | | | | |  |  |  |  |  |  |  |  |
|  | rs62075816 | 1 | 5 | *UBE2Z* | | 7.30E-45 | Whole Blood | | | | | | | | |  |  |  |  |  |  |  |  |
|  |  |  |  | *SNF8* | | 2.70E-07 | Artery Aorta | | | | | | | | |  |  |  |  |  |  |  |  |
|  |  |  |  | [*SUMO2P17*](javascript:EqtlsPage.methods.gotoGeneExpression('ENSG00000248278.1')) | | 6.60E-09 | Artery Tibial | | | | | | | | |  |  |  |  |  |  |  |  |
|  |  |  |  | [*ATP5G1*](javascript:EqtlsPage.methods.gotoGeneExpression('ENSG00000159199.13')) | | 1.50E-15 | [Heart - Left Ventricle](file:///E:\..\..\javascript\EqtlsPage.methods.goTissuePage('Heart_Left_Ventricle')) | | | | | | | | |  |  |  |  |  |  |  |  |
|  | rs4793991 | 1 | 6 | *UBE2Z* | | 2.30E-44 | Whole Blood | | | | | | | | |  |  |  |  |  |  |  |  |
|  |  |  |  | *SNF8* | | 5.10E-08 | Artery Aorta | | | | | | | | |  |  |  |  |  |  |  |  |
|  |  |  |  | [*SUMO2P17*](javascript:EqtlsPage.methods.gotoGeneExpression('ENSG00000248278.1')) | | 3.60E-09 | Artery Tibial | | | | | | | | |  |  |  |  |  |  |  |  |
|  |  |  |  | [*ATP5G1*](javascript:EqtlsPage.methods.gotoGeneExpression('ENSG00000159199.13')) | | 5.60E-15 | [Heart - Left Ventricle](file:///E:\..\..\javascript\EqtlsPage.methods.goTissuePage('Heart_Left_Ventricle')) | | | | | | | | |  |  |  |  |  |  |  |  |
|  | rs318096 | 1 | 4 | *UBE2Z* | | 7.30E-45 | Whole Blood | | | | | | | | |  |  |  |  |  |  |  |  |
|  |  |  |  | *SNF8* | | 2.70E-07 | Artery Aorta | | | | | | | | |  |  |  |  |  |  |  |  |
|  |  |  |  | [*SUMO2P17*](javascript:EqtlsPage.methods.gotoGeneExpression('ENSG00000248278.1')) | | 6.60E-09 | Artery Tibial | | | | | | | | |  |  |  |  |  |  |  |  |
|  |  |  |  | [*ATP5G1*](javascript:EqtlsPage.methods.gotoGeneExpression('ENSG00000159199.13')) | | 5.60E-15 | [Heart - Left Ventricle](file:///E:\..\..\javascript\EqtlsPage.methods.goTissuePage('Heart_Left_Ventricle')) | | | | | | | | |  |  |  |  |  |  |  |  |
|  | rs77877201 | 1 | 6 | *UBE2Z* | | 7.30E-45 | Whole Blood | | | | | | | | |  |  |  |  |  |  |  |  |
|  |  |  |  | *SNF8* | | 2.70E-07 | Artery Aorta | | | | | | | | |  |  |  |  |  |  |  |  |
|  |  |  |  | [*SUMO2P17*](javascript:EqtlsPage.methods.gotoGeneExpression('ENSG00000248278.1')) | | 6.60E-09 | Artery Tibial | | | | | | | | |  |  |  |  |  |  |  |  |
|  |  |  |  | [*ATP5G1*](javascript:EqtlsPage.methods.gotoGeneExpression('ENSG00000159199.13')) | | 2.30E-15 | [Heart - Left Ventricle](file:///E:\..\..\javascript\EqtlsPage.methods.goTissuePage('Heart_Left_Ventricle')) | | | | | | | | |  |  |  |  |  |  |  |  |
|  | rs62075844 | 1 | 7 | *UBE2Z* | | 2.80E-44 | Whole Blood | | | | | | | | |  |  |  |  |  |  |  |  |
|  |  |  |  | *SNF8* | | 1.20E-07 | Artery Aorta | | | | | | | | |  |  |  |  |  |  |  |  |
|  |  |  |  | [*SUMO2P17*](javascript:EqtlsPage.methods.gotoGeneExpression('ENSG00000248278.1')) | | 7.20E-09 | Artery Tibial | | | | | | | | |  |  |  |  |  |  |  |  |
|  |  |  |  | [*ATP5G1*](javascript:EqtlsPage.methods.gotoGeneExpression('ENSG00000159199.13')) | | 2.30E-15 | [Heart - Left Ventricle](file:///E:\..\..\javascript\EqtlsPage.methods.goTissuePage('Heart_Left_Ventricle')) | | | | | | | | |  |  |  |  |  |  |  |  |
|  | rs62075845 | 1 | 7 | *UBE2Z* | | 1.50E-44 | Whole Blood | | | | | | | | |  |  |  |  |  |  |  |  |
|  |  |  |  | *SNF8* | | 9.40E-08 | Artery Aorta | | | | | | | | |  |  |  |  |  |  |  |  |
|  |  |  |  | [*SUMO2P17*](javascript:EqtlsPage.methods.gotoGeneExpression('ENSG00000248278.1')) | | 5.20E-09 | Artery Tibial | | | | | | | | |  |  |  |  |  |  |  |  |
|  |  |  |  | [*ATP5G1*](javascript:EqtlsPage.methods.gotoGeneExpression('ENSG00000159199.13')) | | 5.60E-15 | [Heart - Left Ventricle](file:///E:\..\..\javascript\EqtlsPage.methods.goTissuePage('Heart_Left_Ventricle')) | | | | | | | | |  |  |  |  |  |  |  |  |
|  | rs318095 | 1 | 7 | *UBE2Z* | | 7.30E-45 | Whole Blood | | | | | | | | |  |  |  |  |  |  |  |  |
|  |  |  |  | *SNF8* | | 2.70E-07 | Artery Aorta | | | | | | | | |  |  |  |  |  |  |  |  |
|  |  |  |  | [*SUMO2P17*](javascript:EqtlsPage.methods.gotoGeneExpression('ENSG00000248278.1')) | | 6.60E-09 | Artery Tibial | | | | | | | | |  |  |  |  |  |  |  |  |
|  |  |  |  | [*ATP5G1*](javascript:EqtlsPage.methods.gotoGeneExpression('ENSG00000159199.13')) | | 2.30E-15 | [Heart - Left Ventricle](file:///E:\..\..\javascript\EqtlsPage.methods.goTissuePage('Heart_Left_Ventricle')) | | | | | | | | |  |  |  |  |  |  |  |  |
|  | rs957557 | 1 | 5 | *UBE2Z* | | 1.50E-44 | Whole Blood | | | | | | | | |  |  |  |  |  |  |  |  |
|  |  |  |  | *SNF8* | | 9.40E-08 | Artery Aorta | | | | | | | | |  |  |  |  |  |  |  |  |
|  |  |  |  | [*SUMO2P17*](javascript:EqtlsPage.methods.gotoGeneExpression('ENSG00000248278.1')) | | 5.20E-09 | Artery Tibial | | | | | | | | |  |  |  |  |  |  |  |  |
|  |  |  |  | [*ATP5G1*](javascript:EqtlsPage.methods.gotoGeneExpression('ENSG00000159199.13')) | | 5.60E-15 | [Heart - Left Ventricle](file:///E:\..\..\javascript\EqtlsPage.methods.goTissuePage('Heart_Left_Ventricle')) | | | | | | | | |  |  |  |  |  |  |  |  |
|  | rs832410 | 1 | 5 | *UBE2Z* | | 7.30E-45 | Whole Blood | | | | | | | | |  |  |  |  |  |  |  |  |
|  |  |  |  | *SNF8* | | 2.70E-07 | Artery Aorta | | | | | | | | |  |  |  |  |  |  |  |  |
|  |  |  |  | [*SUMO2P17*](javascript:EqtlsPage.methods.gotoGeneExpression('ENSG00000248278.1')) | | 6.60E-09 | Artery Tibial | | | | | | | | |  |  |  |  |  |  |  |  |
|  |  |  |  | [*ATP5G1*](javascript:EqtlsPage.methods.gotoGeneExpression('ENSG00000159199.13')) | | 5.60E-15 | [Heart - Left Ventricle](file:///E:\..\..\javascript\EqtlsPage.methods.goTissuePage('Heart_Left_Ventricle')) | | | | | | | | |  |  |  |  |  |  |  |  |
|  | rs3080082 | 1 | 4 | *UBE2Z* | | 7.30E-45 | Whole Blood | | | | | | | | |  |  |  |  |  |  |  |  |
|  |  |  |  | *SNF8* | | 2.70E-07 | Artery Aorta | | | | | | | | |  |  |  |  |  |  |  |  |
|  |  |  |  | [*SUMO2P17*](javascript:EqtlsPage.methods.gotoGeneExpression('ENSG00000248278.1')) | | 6.60E-09 | Artery Tibial | | | | | | | | |  |  |  |  |  |  |  |  |
|  |  |  |  | [*ATP5G1*](javascript:EqtlsPage.methods.gotoGeneExpression('ENSG00000159199.13')) | | 2.30E-15 | [Heart - Left Ventricle](file:///E:\..\..\javascript\EqtlsPage.methods.goTissuePage('Heart_Left_Ventricle')) | | | | | | | | |  |  |  |  |  |  |  |  |
|  | **rs15563** | **1** | **1f** | ***UBE2Z*** | | **4.30E-45** | **Whole Blood** | | | | | | | | |  |  |  |  |  |  |  |  |
|  |  |  |  | *SNF8* | | 9.40E-08 | Artery Aorta | | | | | | | | |  |  |  |  |  |  |  |  |
|  |  |  |  | [*SUMO2P17*](javascript:EqtlsPage.methods.gotoGeneExpression('ENSG00000248278.1')) | | 1.10E-08 | Artery Tibial | | | | | | | | |  |  |  |  |  |  |  |  |
|  |  |  |  | [*ATP5G1*](javascript:EqtlsPage.methods.gotoGeneExpression('ENSG00000159199.13')) | | 5.60E-15 | [Heart - Left Ventricle](file:///E:\..\..\javascript\EqtlsPage.methods.goTissuePage('Heart_Left_Ventricle')) | | | | | | | | |  |  |  |  |  |  |  |  |
|  | rs519537 | 1 | 5 | *UBE2Z* | | 7.30E-45 | Whole Blood | | | | | | | | |  |  |  |  |  |  |  |  |
|  |  |  |  | *SNF8* | | 2.70E-07 | Artery Aorta | | | | | | | | |  |  |  |  |  |  |  |  |
|  |  |  |  | [*SUMO2P17*](javascript:EqtlsPage.methods.gotoGeneExpression('ENSG00000248278.1')) | | 6.60E-09 | Artery Tibial | | | | | | | | |  |  |  |  |  |  |  |  |
|  |  |  |  | [*ATP5G1*](javascript:EqtlsPage.methods.gotoGeneExpression('ENSG00000159199.13')) | | 2.30E-15 | [Heart - Left Ventricle](file:///E:\..\..\javascript\EqtlsPage.methods.goTissuePage('Heart_Left_Ventricle')) | | | | | | | | |  |  |  |  |  |  |  |  |
|  | **rs12601672** | **1** | **2b** | ***UBE2Z*** | | **4.30E-45** | **Whole Blood** | | | | | | | | |  |  |  |  |  |  |  |  |
|  |  |  |  | *SNF8* | | 9.40E-08 | Artery Aorta | | | | | | | | |  |  |  |  |  |  |  |  |
|  |  |  |  | [*SUMO2P17*](javascript:EqtlsPage.methods.gotoGeneExpression('ENSG00000248278.1')) | | 1.10E-08 | Artery Tibial | | | | | | | | |  |  |  |  |  |  |  |  |
|  |  |  |  | [*ATP5G1*](javascript:EqtlsPage.methods.gotoGeneExpression('ENSG00000159199.13')) | | 2.30E-15 | [Heart - Left Ventricle](file:///E:\..\..\javascript\EqtlsPage.methods.goTissuePage('Heart_Left_Ventricle')) | | | | | | | | |  |  |  |  |  |  |  |  |
|  | rs17635252 | 1 | 3a | *UBE2Z* | | 4.30E-45 | Whole Blood | | | | | | | | |  |  |  |  |  |  |  |  |
|  |  |  |  | *SNF8* | | 9.40E-08 | Artery Aorta | | | | | | | | |  |  |  |  |  |  |  |  |
|  |  |  |  | [*SUMO2P17*](javascript:EqtlsPage.methods.gotoGeneExpression('ENSG00000248278.1')) | | 1.10E-08 | Artery Tibial | | | | | | | | |  |  |  |  |  |  |  |  |
|  |  |  |  | [*ATP5G1*](javascript:EqtlsPage.methods.gotoGeneExpression('ENSG00000159199.13')) | | 5.60E-15 | [Heart - Left Ventricle](file:///E:\..\..\javascript\EqtlsPage.methods.goTissuePage('Heart_Left_Ventricle')) | | | | | | | | |  |  |  |  |  |  |  |  |
|  | **rs35130875** | **1** | **2b** | ***UBE2Z*** | | **7.30E-45** | **Whole Blood** | | | | | | | | |  |  |  |  |  |  |  |  |
|  |  |  |  | *SNF8* | | 2.70E-07 | Artery Aorta | | | | | | | | |  |  |  |  |  |  |  |  |
|  |  |  |  | [*SUMO2P17*](javascript:EqtlsPage.methods.gotoGeneExpression('ENSG00000248278.1')) | | 6.60E-09 | Artery Tibial | | | | | | | | |  |  |  |  |  |  |  |  |
|  |  |  |  | [*ATP5G1*](javascript:EqtlsPage.methods.gotoGeneExpression('ENSG00000159199.13')) | | 2.30E-15 | [Heart - Left Ventricle](file:///E:\..\..\javascript\EqtlsPage.methods.goTissuePage('Heart_Left_Ventricle')) | | | | | | | | |  |  |  |  |  |  |  |  |
|  | **rs4793992** | **1** | **1f** | ***UBE2Z*** | | **4.30E-45** | **Whole Blood** | | | | | | | | |  |  |  |  |  |  |  |  |
|  |  |  |  | ***SNF8*** | | **9.40E-08** | **Artery Aorta** | | | | | | | | |  |  |  |  |  |  |  |  |
|  |  |  |  | [*SUMO2P17*](javascript:EqtlsPage.methods.gotoGeneExpression('ENSG00000248278.1')) | | 1.10E-08 | Artery Tibial | | | | | | | | |  |  |  |  |  |  |  |  |
|  |  |  |  | [*ATP5G1*](javascript:EqtlsPage.methods.gotoGeneExpression('ENSG00000159199.13')) | | 2.30E-15 | [Heart - Left Ventricle](file:///E:\..\..\javascript\EqtlsPage.methods.goTissuePage('Heart_Left_Ventricle')) | | | | | | | | |  |  |  |  |  |  |  |  |
|  | rs56046215 | 1 | 4 | *UBE2Z* | | 1.50E-44 | Whole Blood | | | | | | | | |  |  |  |  |  |  |  |  |
|  |  |  |  | *SNF8* | | 9.40E-08 | Artery Aorta | | | | | | | | |  |  |  |  |  |  |  |  |
|  |  |  |  | [*SUMO2P17*](javascript:EqtlsPage.methods.gotoGeneExpression('ENSG00000248278.1')) | | 5.20E-09 | Artery Tibial | | | | | | | | |  |  |  |  |  |  |  |  |
|  |  |  |  | [*ATP5G1*](javascript:EqtlsPage.methods.gotoGeneExpression('ENSG00000159199.13')) | | 2.30E-15 | [Heart - Left Ventricle](file:///E:\..\..\javascript\EqtlsPage.methods.goTissuePage('Heart_Left_Ventricle')) | | | | | | | | |  |  |  |  |  |  |  |  |
|  | rs4399576 | 1 | 4 | *UBE2Z* | | 1.50E-44 | Whole Blood | | | | | | | | |  |  |  |  |  |  |  |  |
|  |  |  |  | *SNF8* | | 9.40E-08 | Artery Aorta | | | | | | | | |  |  |  |  |  |  |  |  |
|  |  |  |  | [*SUMO2P17*](javascript:EqtlsPage.methods.gotoGeneExpression('ENSG00000248278.1')) | | 5.20E-09 | Artery Tibial | | | | | | | | |  |  |  |  |  |  |  |  |
|  |  |  |  | [*ATP5G1*](javascript:EqtlsPage.methods.gotoGeneExpression('ENSG00000159199.13')) | | 2.30E-15 | [Heart - Left Ventricle](file:///E:\..\..\javascript\EqtlsPage.methods.goTissuePage('Heart_Left_Ventricle')) | | | | | | | | |  |  |  |  |  |  |  |  |
|  | rs12602179 | 1 | 5 | *UBE2Z* | | 1.50E-44 | Whole Blood | | | | | | | | |  |  |  |  |  |  |  |  |
|  |  |  |  | *SNF8* | | 9.40E-08 | Artery Aorta | | | | | | | | |  |  |  |  |  |  |  |  |
|  |  |  |  | [*SUMO2P17*](javascript:EqtlsPage.methods.gotoGeneExpression('ENSG00000248278.1')) | | 5.20E-09 | Artery Tibial | | | | | | | | |  |  |  |  |  |  |  |  |
|  |  |  |  | [*ATP5G1*](javascript:EqtlsPage.methods.gotoGeneExpression('ENSG00000159199.13')) | | 2.30E-15 | [Heart - Left Ventricle](file:///E:\..\..\javascript\EqtlsPage.methods.goTissuePage('Heart_Left_Ventricle')) | | | | | | | | |  |  |  |  |  |  |  |  |
|  | rs17708633 | 1 | 5 | *UBE2Z* | | 4.30E-45 | Whole Blood | | | | | | | | |  |  |  |  |  |  |  |  |
|  |  |  |  | *SNF8* | | 9.40E-08 | Artery Aorta | | | | | | | | |  |  |  |  |  |  |  |  |
|  |  |  |  | [*SUMO2P17*](javascript:EqtlsPage.methods.gotoGeneExpression('ENSG00000248278.1')) | | 1.10E-08 | Artery Tibial | | | | | | | | |  |  |  |  |  |  |  |  |
|  |  |  |  | [*ATP5G1*](javascript:EqtlsPage.methods.gotoGeneExpression('ENSG00000159199.13')) | | 2.30E-15 | [Heart - Left Ventricle](file:///E:\..\..\javascript\EqtlsPage.methods.goTissuePage('Heart_Left_Ventricle')) | | | | | | | | |  |  |  |  |  |  |  |  |
|  | rs57901004 | 1 | 7 | *UBE2Z* | | 4.30E-45 | Whole Blood | | | | | | | | |  |  |  |  |  |  |  |  |
|  |  |  |  | *SNF8* | | 9.40E-08 | Artery Aorta | | | | | | | | |  |  |  |  |  |  |  |  |
|  |  |  |  | [*SUMO2P17*](javascript:EqtlsPage.methods.gotoGeneExpression('ENSG00000248278.1')) | | 1.10E-08 | Artery Tibial | | | | | | | | |  |  |  |  |  |  |  |  |
|  |  |  |  | [*ATP5G1*](javascript:EqtlsPage.methods.gotoGeneExpression('ENSG00000159199.13')) | | 2.30E-15 | [Heart - Left Ventricle](file:///E:\..\..\javascript\EqtlsPage.methods.goTissuePage('Heart_Left_Ventricle')) | | | | | | | | |  |  |  |  |  |  |  |  |
|  | rs3215074 | 1 | 3a | *No data* | | |  |  |  |  |  |  |  |  |  |  |  |  |  |  |  |  |  |
|  | **rs1994970** | **1** | **1f** | ***UBE2Z*** | ***4.30E-45*** | | | **Whole Blood** | | | | |  |  |  |  |  |  |  |  |  |  |  |
|  |  |  |  | ***SNF8*** | ***9.40E-08*** | | | **Artery Aorta** | | | | |  |  |  |  |  |  |  |  |  |  |  |
|  |  |  |  | [***SUMO2P17***](javascript:EqtlsPage.methods.gotoGeneExpression('ENSG00000248278.1')) | ***1.10E-08*** | | | **Artery Tibial** | | | | |  |  |  |  |  |  |  |  |  |  |  |
|  |  |  |  | [***ATP5G1***](javascript:EqtlsPage.methods.gotoGeneExpression('ENSG00000159199.13')) | ***2.30E-15*** | | | [**Heart - Left Ventricle**](file:///E:\..\..\javascript\EqtlsPage.methods.goTissuePage('Heart_Left_Ventricle')) | | | | |  |  |  |  |  |  |  |  |  |  |  |
|  | rs75245975 | 1 | 6 | *UBE2Z* | *4.30E-45* | | | Whole Blood | | | | |  |  |  |  |  |  |  |  |  |  |  |
|  |  |  |  | *SNF8* | *9.40E-08* | | | Artery Aorta | | | | |  |  |  |  |  |  |  |  |  |  |  |
|  |  |  |  | [*SUMO2P17*](javascript:EqtlsPage.methods.gotoGeneExpression('ENSG00000248278.1')) | *1.10E-08* | | | Artery Tibial | | | | |  |  |  |  |  |  |  |  |  |  |  |
|  |  |  |  | [*ATP5G1*](javascript:EqtlsPage.methods.gotoGeneExpression('ENSG00000159199.13')) | *3.00E-15* | | | [Heart - Left Ventricle](file:///E:\..\..\javascript\EqtlsPage.methods.goTissuePage('Heart_Left_Ventricle')) | | | | |  |  |  |  |  |  |  |  |  |  |  |
|  | rs9747646 | 1 | 6 | *UBE2Z* | *5.70E-45* | | | Whole Blood | | | | |  |  |  |  |  |  |  |  |  |  |  |
|  |  |  |  | *SNF8* | *2.10E-07* | | | Artery Aorta | | | | |  |  |  |  |  |  |  |  |  |  |  |
|  |  |  |  | [*SUMO2P17*](javascript:EqtlsPage.methods.gotoGeneExpression('ENSG00000248278.1')) | *1.10E-08* | | | Artery Tibial | | | | |  |  |  |  |  |  |  |  |  |  |  |
|  |  |  |  | [*ATP5G1*](javascript:EqtlsPage.methods.gotoGeneExpression('ENSG00000159199.13')) | *3.00E-15* | | | [Heart - Left Ventricle](file:///E:\..\..\javascript\EqtlsPage.methods.goTissuePage('Heart_Left_Ventricle')) | | | | |  |  |  |  |  |  |  |  |  |  |  |
|  | rs62075852 | 1 | No data | *UBE2Z* | *5.70E-45* | | | Whole Blood | | | | |  |  |  |  |  |  |  |  |  |  |  |
|  |  |  |  | *SNF8* | *2.10E-07* | | | Artery Aorta | | | | |  |  |  |  |  |  |  |  |  |  |  |
|  |  |  |  | [*SUMO2P17*](javascript:EqtlsPage.methods.gotoGeneExpression('ENSG00000248278.1')) | *1.10E-08* | | | Artery Tibial | | | | |  |  |  |  |  |  |  |  |  |  |  |
|  |  |  |  | [*ATP5G1*](javascript:EqtlsPage.methods.gotoGeneExpression('ENSG00000159199.13')) | *3.00E-15* | | | [Heart - Left Ventricle](file:///E:\..\..\javascript\EqtlsPage.methods.goTissuePage('Heart_Left_Ventricle')) | | | | |  |  |  |  |  |  |  |  |  |  |  |
|  | rs12601858 | 1 | No data | *UBE2Z* | *5.70E-45* | | | Whole Blood | | | | |  |  |  |  |  |  |  |  |  |  |  |
|  |  |  |  | *SNF8* | *2.10E-07* | | | Artery Aorta | | | | |  |  |  |  |  |  |  |  |  |  |  |
|  |  |  |  | [*SUMO2P17*](javascript:EqtlsPage.methods.gotoGeneExpression('ENSG00000248278.1')) | *1.10E-08* | | | Artery Tibial | | | | |  |  |  |  |  |  |  |  |  |  |  |
|  |  |  |  | [*ATP5G1*](javascript:EqtlsPage.methods.gotoGeneExpression('ENSG00000159199.13')) | *1.10E-14* | | | [Heart - Left Ventricle](file:///E:\..\..\javascript\EqtlsPage.methods.goTissuePage('Heart_Left_Ventricle')) | | | | |  |  |  |  |  |  |  |  |  |  |  |
|  | rs55724082 | 1 | No data | *UBE2Z* | *1.50E-44* | | | Whole Blood | | | | |  |  |  |  |  |  |  |  |  |  |  |
|  |  |  |  | *SNF8* | *3.00E-07* | | | Artery Aorta | | | | |  |  |  |  |  |  |  |  |  |  |  |
|  |  |  |  | [*SUMO2P17*](javascript:EqtlsPage.methods.gotoGeneExpression('ENSG00000248278.1')) | *1.20E-08* | | | Artery Tibial | | | | |  |  |  |  |  |  |  |  |  |  |  |
|  |  |  |  | [*ATP5G1*](javascript:EqtlsPage.methods.gotoGeneExpression('ENSG00000159199.13')) | *6.30E-15* | | | [Heart - Left Ventricle](file:///E:\..\..\javascript\EqtlsPage.methods.goTissuePage('Heart_Left_Ventricle')) | | | | |  |  |  |  |  |  |  |  |  |  |  |
|  | rs8182364 | 1 | 3a | *UBE2Z* | *6.60E-44* | | | Whole Blood | | | | |  |  |  |  |  |  |  |  |  |  |  |
|  |  |  |  | *SNF8* | *2.10E-07* | | | Artery Aorta | | | | |  |  |  |  |  |  |  |  |  |  |  |
|  |  |  |  | [*SUMO2P17*](javascript:EqtlsPage.methods.gotoGeneExpression('ENSG00000248278.1')) | *1.10E-08* | | | Artery Tibial | | | | |  |  |  |  |  |  |  |  |  |  |  |
|  |  |  |  | [*ATP5G1*](javascript:EqtlsPage.methods.gotoGeneExpression('ENSG00000159199.13')) | *6.3e-15* | | | [Heart - Left Ventricle](file:///E:\..\..\javascript\EqtlsPage.methods.goTissuePage('Heart_Left_Ventricle')) | | | | |  |  |  |  |  |  |  |  |  |  |  |
|  |  |  |  |  | *No Data* | | |  | | | | |  |  |  |  |  |  |  |  |  |  |  |
|  | rs56080003 | 1 | 6 |  |  | | |  | | | | |  |  |  |  |  |  |  |  |  |  |  |
|  |  |  |  |  |  | | |  | | | | |  |  |  |  |  |  |  |  |  |  |  |
|  |  |  |  |  |  | | |  | | | | |  |  |  |  |  |  |  |  |  |  |  |
|  |  |  |  | [*ATP5G1*](javascript:EqtlsPage.methods.gotoGeneExpression('ENSG00000159199.13')) | *3.00E-15* | | | [Heart - Left Ventricle](file:///E:\..\..\javascript\EqtlsPage.methods.goTissuePage('Heart_Left_Ventricle')) | | | | |  |  |  |  |  |  |  |  |  |  |  |
|  | rs4793995 | 1 | 5 | *UBE2Z* | *1.40E-44* | | | Whole Blood | | | | |  |  |  |  |  |  |  |  |  |  |  |
|  |  |  |  | *SNF8* | *2.10E-07* | | | Artery Aorta | | | | |  |  |  |  |  |  |  |  |  |  |  |
|  |  |  |  | [*SUMO2P17*](javascript:EqtlsPage.methods.gotoGeneExpression('ENSG00000248278.1')) | *1.30E-08* | | | Artery Tibial | | | | |  |  |  |  |  |  |  |  |  |  |  |
|  |  |  |  | [*ATP5G1*](javascript:EqtlsPage.methods.gotoGeneExpression('ENSG00000159199.13')) | *3.00E-15* | | | [Heart - Left Ventricle](file:///E:\..\..\javascript\EqtlsPage.methods.goTissuePage('Heart_Left_Ventricle')) | | | | |  |  |  |  |  |  |  |  |  |  |  |
|  | rs4793996 | 1 | No data | *UBE2Z* | *1.40E-44* | | | Whole Blood | | | | |  |  |  |  |  |  |  |  |  |  |  |
|  |  |  |  | *SNF8* | *2.10E-07* | | | Artery Aorta | | | | |  |  |  |  |  |  |  |  |  |  |  |
|  |  |  |  | [*SUMO2P17*](javascript:EqtlsPage.methods.gotoGeneExpression('ENSG00000248278.1')) | *1.30E-08* | | | Artery Tibial | | | | |  |  |  |  |  |  |  |  |  |  |  |
|  |  |  |  | [*ATP5G1*](javascript:EqtlsPage.methods.gotoGeneExpression('ENSG00000159199.13')) | *3.80E-15* | | | [Heart - Left Ventricle](file:///E:\..\..\javascript\EqtlsPage.methods.goTissuePage('Heart_Left_Ventricle')) | | | | |  |  |  |  |  |  |  |  |  |  |  |
|  | rs4793997 | 1 | No data | *UBE2Z* | *3.70E-44* | | | Whole Blood | | | | |  |  |  |  |  |  |  |  |  |  |  |
|  |  |  |  | *SNF8* | *2.10E-07* | | | Artery Aorta | | | | |  |  |  |  |  |  |  |  |  |  |  |
|  |  |  |  | [*SUMO2P17*](javascript:EqtlsPage.methods.gotoGeneExpression('ENSG00000248278.1')) | *1.20E-08* | | | Artery Tibial | | | | |  |  |  |  |  |  |  |  |  |  |  |
|  |  |  |  | [*ATP5G1*](javascript:EqtlsPage.methods.gotoGeneExpression('ENSG00000159199.13')) | *3.00E-15* | | | [Heart - Left Ventricle](file:///E:\..\..\javascript\EqtlsPage.methods.goTissuePage('Heart_Left_Ventricle')) | | | | |  |  |  |  |  |  |  |  |  |  |  |
|  | rs4793998 | 1 | 5 | *UBE2Z* | *1.40E-44* | | | Whole Blood | | | | |  |  |  |  |  |  |  |  |  |  |  |
|  |  |  |  | *SNF8* | *2.10E-07* | | | Artery Aorta | | | | |  |  |  |  |  |  |  |  |  |  |  |
|  |  |  |  | [*SUMO2P17*](javascript:EqtlsPage.methods.gotoGeneExpression('ENSG00000248278.1')) | *1.30E-08* | | | Artery Tibial | | | | |  |  |  |  |  |  |  |  |  |  |  |
|  |  |  |  | [*ATP5G1*](javascript:EqtlsPage.methods.gotoGeneExpression('ENSG00000159199.13')) | *2.40E-15* | | | [Heart - Left Ventricle](file:///E:\..\..\javascript\EqtlsPage.methods.goTissuePage('Heart_Left_Ventricle')) | | | | |  |  |  |  |  |  |  |  |  |  |  |
|  | rs12603612 | 1 | 4 | *UBE2Z* | *2.30E-44* | | | Whole Blood | | | | |  |  |  |  |  |  |  |  |  |  |  |
|  |  |  |  | *SNF8* | *2.10E-07* | | | Artery Aorta | | | | |  |  |  |  |  |  |  |  |  |  |  |
|  |  |  |  | [*SUMO2P17*](javascript:EqtlsPage.methods.gotoGeneExpression('ENSG00000248278.1')) | *1.30E-08* | | | Artery Tibial | | | | |  |  |  |  |  |  |  |  |  |  |  |
|  |  |  |  | [*ATP5G1*](javascript:EqtlsPage.methods.gotoGeneExpression('ENSG00000159199.13')) | *2.40E-15* | | | [Heart - Left Ventricle](file:///E:\..\..\javascript\EqtlsPage.methods.goTissuePage('Heart_Left_Ventricle')) | | | | |  |  |  |  |  |  |  |  |  |  |  |
|  | rs2411375 | 1 | 4 | *UBE2Z* | *2.30E-44* | | | Whole Blood | | | | |  |  |  |  |  |  |  |  |  |  |  |
|  |  |  |  | *SNF8* | *2.10E-07* | | | Artery Aorta | | | | |  |  |  |  |  |  |  |  |  |  |  |
|  |  |  |  | [*SUMO2P17*](javascript:EqtlsPage.methods.gotoGeneExpression('ENSG00000248278.1')) | *1.30E-08* | | | Artery Tibial | | | | |  |  |  |  |  |  |  |  |  |  |  |
|  |  |  |  | [*ATP5G1*](javascript:EqtlsPage.methods.gotoGeneExpression('ENSG00000159199.13')) | *2.40E-15* | | | [Heart - Left Ventricle](file:///E:\..\..\javascript\EqtlsPage.methods.goTissuePage('Heart_Left_Ventricle')) | | | | |  |  |  |  |  |  |  |  |  |  |  |
|  | rs2270574 | 1 | 4 | *UBE2Z* | *2.30E-44* | | | Whole Blood | | | | |  |  |  |  |  |  |  |  |  |  |  |
|  |  |  |  | *SNF8* | *2.10E-07* | | | Artery Aorta | | | | |  |  |  |  |  |  |  |  |  |  |  |
|  |  |  |  | [*SUMO2P17*](javascript:EqtlsPage.methods.gotoGeneExpression('ENSG00000248278.1')) | *1.30E-08* | | | Artery Tibial | | | | |  |  |  |  |  |  |  |  |  |  |  |
|  |  |  |  | [*ATP5G1*](javascript:EqtlsPage.methods.gotoGeneExpression('ENSG00000159199.13')) | *2.40E-15* | | | [Heart - Left Ventricle](file:///E:\..\..\javascript\EqtlsPage.methods.goTissuePage('Heart_Left_Ventricle')) | | | | |  |  |  |  |  |  |  |  |  |  |  |
|  | rs4794000 | 1 | 5 | *UBE2Z* | *2.30E-44* | | | Whole Blood | | | | |  |  |  |  |  |  |  |  |  |  |  |
|  |  |  |  | *SNF8* | *2.10E-07* | | | Artery Aorta | | | | |  |  |  |  |  |  |  |  |  |  |  |
|  |  |  |  | [*SUMO2P17*](javascript:EqtlsPage.methods.gotoGeneExpression('ENSG00000248278.1')) | *1.30E-08* | | | Artery Tibial | | | | |  |  |  |  |  |  |  |  |  |  |  |
|  |  |  |  | [*ATP5G1*](javascript:EqtlsPage.methods.gotoGeneExpression('ENSG00000159199.13')) | *2.40E-15* | | | [Heart - Left Ventricle](file:///E:\..\..\javascript\EqtlsPage.methods.goTissuePage('Heart_Left_Ventricle')) | | | | |  |  |  |  |  |  |  |  |  |  |  |
|  | rs2898705 | 1 | No data | *UBE2Z* | *2.30E-44* | | | Whole Blood | | | | |  |  |  |  |  |  |  |  |  |  |  |
|  |  |  |  | *SNF8* | *2.10E-07* | | | Artery Aorta | | | | |  |  |  |  |  |  |  |  |  |  |  |
|  |  |  |  | [*SUMO2P17*](javascript:EqtlsPage.methods.gotoGeneExpression('ENSG00000248278.1')) | *1.30E-08* | | | Artery Tibial | | | | |  |  |  |  |  |  |  |  |  |  |  |
|  |  |  |  | [*ATP5G1*](javascript:EqtlsPage.methods.gotoGeneExpression('ENSG00000159199.13')) | *1.50E-15* | | | [Heart - Left Ventricle](file:///E:\..\..\javascript\EqtlsPage.methods.goTissuePage('Heart_Left_Ventricle')) | | | | |  |  |  |  |  |  |  |  |  |  |  |
|  | rs2411374 | 1 | No data | *UBE2Z* | *2.10E-44* | | | Whole Blood | | | | |  |  |  |  |  |  |  |  |  |  |  |
|  |  |  |  | *SNF8* | *1.50E-07* | | | Artery Aorta | | | | |  |  |  |  |  |  |  |  |  |  |  |
|  |  |  |  | [*SUMO2P17*](javascript:EqtlsPage.methods.gotoGeneExpression('ENSG00000248278.1')) | *3.50E-08* | | | Artery Tibial | | | | |  |  |  |  |  |  |  |  |  |  |  |
|  |  |  |  | [*ATP5G1*](javascript:EqtlsPage.methods.gotoGeneExpression('ENSG00000159199.13')) | *8.80E-15* | | | [Heart - Left Ventricle](file:///E:\..\..\javascript\EqtlsPage.methods.goTissuePage('Heart_Left_Ventricle')) | | | | |  |  |  |  |  |  |  |  |  |  |  |
|  | rs62075850 | 0.996 | No data | *UBE2Z* | *3.40E-45* | | | Whole Blood | | | | |  |  |  |  |  |  |  |  |  |  |  |
|  |  |  |  | *SNF8* | *1.00E-07* | | | Artery Aorta | | | | |  |  |  |  |  |  |  |  |  |  |  |
|  |  |  |  | [*SUMO2P17*](javascript:EqtlsPage.methods.gotoGeneExpression('ENSG00000248278.1')) | *7.40E-09* | | | Artery Tibial | | | | |  |  |  |  |  |  |  |  |  |  |  |
|  | rs148914713 | 0.996 | No data | *No data* | | | |  | | | | |  |  |  |  |  |  |  |  |  |  |  |
|  | rs62075838 | 0.996 | 6 | *No data* | | | |  | | | | |  |  |  |  |  |  |  |  |  |  |  |
|  | rs62075839 | 0.996 | 6 | *No data* | | | |  | | | | |  |  |  |  |  |  |  |  |  |  |  |
|  | rs8070060 | 0.996 | 7 | [*ATP5G1*](javascript:EqtlsPage.methods.gotoGeneExpression('ENSG00000159199.13')) | *2.00E-15* | | | [Heart - Left Ventricle](file:///E:\..\..\javascript\EqtlsPage.methods.goTissuePage('Heart_Left_Ventricle')) | | | | |  |  |  |  |  |  |  |  |  |  |  |
|  |  |  |  | *UBE2Z* | *5.70E-43* | | | Whole Blood | | | | |  |  |  |  |  |  |  |  |  |  |  |
|  |  |  |  | *SNF8* | *3.30E-08* | | | Artery Aorta | | | | |  |  |  |  |  |  |  |  |  |  |  |
|  |  |  |  | [*SUMO2P17*](javascript:EqtlsPage.methods.gotoGeneExpression('ENSG00000248278.1')) | *2.30E-09* | | | Artery Tibial | | | | |  |  |  |  |  |  |  |  |  |  |  |
|  | rs62075846 | 0.996 | 7 | [*ATP5G1*](javascript:EqtlsPage.methods.gotoGeneExpression('ENSG00000159199.13')) | *2.30E-15* | | | [Heart - Left Ventricle](file:///E:\..\..\javascript\EqtlsPage.methods.goTissuePage('Heart_Left_Ventricle')) | | | | |  |  |  |  |  |  |  |  |  |  |  |
|  |  |  |  | *UBE2Z* | *1.50E-44* | | | Whole Blood | | | | |  |  |  |  |  |  |  |  |  |  |  |
|  |  |  |  | *SNF8* | *9.40E-08* | | | Artery Aorta | | | | |  |  |  |  |  |  |  |  |  |  |  |
|  |  |  |  | [*SUMO2P17*](javascript:EqtlsPage.methods.gotoGeneExpression('ENSG00000248278.1')) | *5.20E-09* | | | Artery Tibial | | | | |  |  |  |  |  |  |  |  |  |  |  |
|  | rs1057897 | 0.996 | 4 | [*ATP5G1*](javascript:EqtlsPage.methods.gotoGeneExpression('ENSG00000159199.13')) | *4.90E-15* | | | [Heart - Left Ventricle](file:///E:\..\..\javascript\EqtlsPage.methods.goTissuePage('Heart_Left_Ventricle')) | | | | |  |  |  |  |  |  |  |  |  |  |  |
|  |  |  |  | *UBE2Z* | *1.10E-43* | | | Whole Blood | | | | |  |  |  |  |  |  |  |  |  |  |  |
|  |  |  |  | *SNF8* | *2.50E-07* | | | Artery Aorta | | | | |  |  |  |  |  |  |  |  |  |  |  |
|  |  |  |  | [*SUMO2P17*](javascript:EqtlsPage.methods.gotoGeneExpression('ENSG00000248278.1')) | *2.00E-08* | | | Artery Tibial | | | | |  |  |  |  |  |  |  |  |  |  |  |
|  | rs72523750 | 0.996 | No data | [*ATP5G1*](javascript:EqtlsPage.methods.gotoGeneExpression('ENSG00000159199.13')) | *2.30E-15* | | | [Heart - Left Ventricle](file:///E:\..\..\javascript\EqtlsPage.methods.goTissuePage('Heart_Left_Ventricle')) | | | | |  |  |  |  |  |  |  |  |  |  |  |
|  |  |  |  | *UBE2Z* | *1.50E-44* | | | Whole Blood | | | | |  |  |  |  |  |  |  |  |  |  |  |
|  |  |  |  | *SNF8* | *9.40E-08* | | | Artery Aorta | | | | |  |  |  |  |  |  |  |  |  |  |  |
|  |  |  |  | [*SUMO2P17*](javascript:EqtlsPage.methods.gotoGeneExpression('ENSG00000248278.1')) | *5.20E-09* | | | Artery Tibial | | | | |  |  |  |  |  |  |  |  |  |  |  |
|  | rs4255820 | 0.992 | 6 | [*ATP5G1*](javascript:EqtlsPage.methods.gotoGeneExpression('ENSG00000159199.13')) | *2.30E-15* | | | [Heart - Left Ventricle](file:///E:\..\..\javascript\EqtlsPage.methods.goTissuePage('Heart_Left_Ventricle')) | | | | |  |  |  |  |  |  |  |  |  |  |  |
|  |  |  |  | *UBE2Z* | *1.70E-44* | | | Whole Blood | | | | |  |  |  |  |  |  |  |  |  |  |  |
|  |  |  |  | *SNF8* | *9.40E-08* | | | Artery Aorta | | | | |  |  |  |  |  |  |  |  |  |  |  |
|  |  |  |  | [*SUMO2P17*](javascript:EqtlsPage.methods.gotoGeneExpression('ENSG00000248278.1')) | *5.50E-09* | | | Artery Tibial | | | | |  |  |  |  |  |  |  |  |  |  |  |
|  | rs145067756 | 0.9801 | 5 |  | *No Data* | | |  | | | | |  |  |  |  |  |  |  |  |  |  |  |
|  |  |  |  |  |  | | |  | | | | |  |  |  |  |  |  |  |  |  |  |  |
|  |  |  |  |  |  | | |  | | | | |  |  |  |  |  |  |  |  |  |  |  |
|  |  |  |  |  |  | | |  | | | | |  |  |  |  |  |  |  |  |  |  |  |
|  | rs9904645 | 0.98 | 4 | [*ATP5G1*](javascript:EqtlsPage.methods.gotoGeneExpression('ENSG00000159199.13')) | *1.90E-14* | | | [Heart - Left Ventricle](file:///E:\..\..\javascript\EqtlsPage.methods.goTissuePage('Heart_Left_Ventricle')) | | | | |  |  |  |  |  |  |  |  |  |  |  |
|  |  |  |  | *UBE2Z* | *2.60E-44* | | | Whole Blood | | | | |  |  |  |  |  |  |  |  |  |  |  |
|  |  |  |  | *SNF8* | *1.40E-06* | | | Artery Aorta | | | | |  |  |  |  |  |  |  |  |  |  |  |
|  |  |  |  | [*SUMO2P17*](javascript:EqtlsPage.methods.gotoGeneExpression('ENSG00000248278.1')) | *1.40E-08* | | | Artery Tibial | | | | |  |  |  |  |  |  |  |  |  |  |  |
|  | rs524808 | 0.98 | 5 | [*ATP5G1*](javascript:EqtlsPage.methods.gotoGeneExpression('ENSG00000159199.13')) | *1.90E-14* | | | [Heart - Left Ventricle](file:///E:\..\..\javascript\EqtlsPage.methods.goTissuePage('Heart_Left_Ventricle')) | | | | |  |  |  |  |  |  |  |  |  |  |  |
|  |  |  |  | *UBE2Z* | *2.60E-44* | | | Whole Blood | | | | |  |  |  |  |  |  |  |  |  |  |  |
|  |  |  |  | *SNF8* | *1.40E-06* | | | Artery Aorta | | | | |  |  |  |  |  |  |  |  |  |  |  |
|  |  |  |  | [*SUMO2P17*](javascript:EqtlsPage.methods.gotoGeneExpression('ENSG00000248278.1')) | *1.40E-08* | | | Artery Tibial | | | | |  |  |  |  |  |  |  |  |  |  |  |
|  | rs1008834 | 0.9761 | 6 | [*ATP5G1*](javascript:EqtlsPage.methods.gotoGeneExpression('ENSG00000159199.13')) | *1.90E-14* | | | [Heart - Left Ventricle](file:///E:\..\..\javascript\EqtlsPage.methods.goTissuePage('Heart_Left_Ventricle')) | | | | |  |  |  |  |  |  |  |  |  |  |  |
|  |  |  |  | *UBE2Z* | *2.60E-44* | | | Whole Blood | | | | |  |  |  |  |  |  |  |  |  |  |  |
|  |  |  |  | *SNF8* | *1.40E-06* | | | Artery Aorta | | | | |  |  |  |  |  |  |  |  |  |  |  |
|  |  |  |  | [*SUMO2P17*](javascript:EqtlsPage.methods.gotoGeneExpression('ENSG00000248278.1')) | *1.40E-08* | | | Artery Tibial | | | | |  |  |  |  |  |  |  |  |  |  |  |
|  | rs1124829 | 0.9761 | 6 |  | *No Data* | | |  | | | | |  |  |  |  |  |  |  |  |  |  |  |
|  |  |  |  |  |  | | |  | | | | |  |  |  |  |  |  |  |  |  |  |  |
|  |  |  |  |  |  | | |  | | | | |  |  |  |  |  |  |  |  |  |  |  |
|  |  |  |  |  |  | | |  | | | | |  |  |  |  |  |  |  |  |  |  |  |
|  | rs1985785 | 0.9761 | 6 | [*ATP5G1*](javascript:EqtlsPage.methods.gotoGeneExpression('ENSG00000159199.13')) | *1.30E-14* | | | [Heart - Left Ventricle](file:///E:\..\..\javascript\EqtlsPage.methods.goTissuePage('Heart_Left_Ventricle')) | | | | |  |  |  |  |  |  |  |  |  |  |  |
|  |  |  |  | *UBE2Z* | *4.90E-45* | | | Whole Blood | | | | |  |  |  |  |  |  |  |  |  |  |  |
|  |  |  |  | *SNF8* | *1.40E-06* | | | Artery Aorta | | | | |  |  |  |  |  |  |  |  |  |  |  |
|  |  |  |  | [*SUMO2P17*](javascript:EqtlsPage.methods.gotoGeneExpression('ENSG00000248278.1')) | *8.50E-09* | | | Artery Tibial | | | | |  |  |  |  |  |  |  |  |  |  |  |
|  | rs602051 | 0.9761 | 6 | [*ATP5G1*](javascript:EqtlsPage.methods.gotoGeneExpression('ENSG00000159199.13')) | *1.90E-14* | | | [Heart - Left Ventricle](file:///E:\..\..\javascript\EqtlsPage.methods.goTissuePage('Heart_Left_Ventricle')) | | | | |  |  |  |  |  |  |  |  |  |  |  |
|  |  |  |  | *UBE2Z* | *2.90E-44* | | | Whole Blood | | | | |  |  |  |  |  |  |  |  |  |  |  |
|  |  |  |  | *SNF8* | *9.90E-07* | | | Artery Aorta | | | | |  |  |  |  |  |  |  |  |  |  |  |
|  |  |  |  | [*SUMO2P17*](javascript:EqtlsPage.methods.gotoGeneExpression('ENSG00000248278.1')) | *1.90E-08* | | | Artery Tibial | | | | |  |  |  |  |  |  |  |  |  |  |  |
|  | rs1057902 | 0.9722 | 3a | [*ATP5G1*](javascript:EqtlsPage.methods.gotoGeneExpression('ENSG00000159199.13')) | *1.20E-13* | | | [Heart - Left Ventricle](file:///E:\..\..\javascript\EqtlsPage.methods.goTissuePage('Heart_Left_Ventricle')) | | | | |  |  |  |  |  |  |  |  |  |  |  |
|  |  |  |  | *UBE2Z* | *8.40E-41* | | | Whole Blood | | | | |  |  |  |  |  |  |  |  |  |  |  |
|  |  |  |  | *SNF8* | *8.00E-07* | | | Artery Aorta | | | | |  |  |  |  |  |  |  |  |  |  |  |
|  |  |  |  | [*SUMO2P17*](javascript:EqtlsPage.methods.gotoGeneExpression('ENSG00000248278.1')) | *2.20E-08* | | | Artery Tibial | | | | |  |  |  |  |  |  |  |  |  |  |  |
|  | rs151215418 | 0.9722 | 6 | *No data* |  | | |  | | | | |  |  |  |  |  |  |  |  |  |  |  |
|  | rs79049364 | 0.9722 | 6 | *No data* |  | | |  | | | | |  |  |  |  |  |  |  |  |  |  |  |
|  | rs80032154 | 0.9722 | 6 | *No data* |  | | |  | | | | |  |  |  |  |  |  |  |  |  |  |  |
|  | rs58591767 | 0.9683 | 7 | [*ATP5G1*](javascript:EqtlsPage.methods.gotoGeneExpression('ENSG00000159199.13')) | *2.70E-14* | | | [Heart - Left Ventricle](file:///E:\..\..\javascript\EqtlsPage.methods.goTissuePage('Heart_Left_Ventricle')) | | | | |  |  |  |  |  |  |  |  |  |  |  |
|  |  |  |  | *UBE2Z* | *3.10E-43* | | | Whole Blood | | | | |  |  |  |  |  |  |  |  |  |  |  |
|  |  |  |  | *SNF8* | *2.20E-07* | | | Artery Aorta | | | | |  |  |  |  |  |  |  |  |  |  |  |
|  |  |  |  | [*SUMO2P17*](javascript:EqtlsPage.methods.gotoGeneExpression('ENSG00000248278.1')) | *6.10E-09* | | | Artery Tibial | | | | |  |  |  |  |  |  |  |  |  |  |  |
|  | rs59270107 | 0.9683 | 7 | [*ATP5G1*](javascript:EqtlsPage.methods.gotoGeneExpression('ENSG00000159199.13')) | *2.70E-14* | | | [Heart - Left Ventricle](file:///E:\..\..\javascript\EqtlsPage.methods.goTissuePage('Heart_Left_Ventricle')) | | | | |  |  |  |  |  |  |  |  |  |  |  |
|  |  |  |  | *UBE2Z* | *3.10E-43* | | | Whole Blood | | | | |  |  |  |  |  |  |  |  |  |  |  |
|  |  |  |  | *SNF8* | *2.20E-07* | | | Artery Aorta | | | | |  |  |  |  |  |  |  |  |  |  |  |
|  |  |  |  | [*SUMO2P17*](javascript:EqtlsPage.methods.gotoGeneExpression('ENSG00000248278.1')) | *6.10E-09* | | | Artery Tibial | | | | |  |  |  |  |  |  |  |  |  |  |  |
|  | rs62078369 | 0.9683 | 7 | [*ATP5G1*](javascript:EqtlsPage.methods.gotoGeneExpression('ENSG00000159199.13')) | *2.70E-14* | | | [Heart - Left Ventricle](file:///E:\..\..\javascript\EqtlsPage.methods.goTissuePage('Heart_Left_Ventricle')) | | | | |  |  |  |  |  |  |  |  |  |  |  |
|  |  |  |  | *UBE2Z* | *3.10E-43* | | | Whole Blood | | | | |  |  |  |  |  |  |  |  |  |  |  |
|  |  |  |  | *SNF8* | *2.20E-07* | | | Artery Aorta | | | | |  |  |  |  |  |  |  |  |  |  |  |
|  |  |  |  | [*SUMO2P17*](javascript:EqtlsPage.methods.gotoGeneExpression('ENSG00000248278.1')) | *6.10E-09* | | | Artery Tibial | | | | |  |  |  |  |  |  |  |  |  |  |  |
|  | rs11079844 | 0.9683 | 1f | [*ATP5G1*](javascript:EqtlsPage.methods.gotoGeneExpression('ENSG00000159199.13')) | *2.90E-14* | | | [Heart - Left Ventricle](file:///E:\..\..\javascript\EqtlsPage.methods.goTissuePage('Heart_Left_Ventricle')) | | | | |  |  |  |  |  |  |  |  |  |  |  |
|  |  |  |  | *UBE2Z* | *3.10E-43* | | | Whole Blood | | | | |  |  |  |  |  |  |  |  |  |  |  |
|  |  |  |  | *SNF8* | *2.20E-07* | | | Artery Aorta | | | | |  |  |  |  |  |  |  |  |  |  |  |
|  |  |  |  | [*SUMO2P17*](javascript:EqtlsPage.methods.gotoGeneExpression('ENSG00000248278.1')) | *5.90E-09* | | | Artery Tibial | | | | |  |  |  |  |  |  |  |  |  |  |  |
|  | rs62078372 | 0.9683 | 5 | [*ATP5G1*](javascript:EqtlsPage.methods.gotoGeneExpression('ENSG00000159199.13')) | *2.90E-14* | | | [Heart - Left Ventricle](file:///E:\..\..\javascript\EqtlsPage.methods.goTissuePage('Heart_Left_Ventricle')) | | | | |  |  |  |  |  |  |  |  |  |  |  |
|  |  |  |  | *UBE2Z* | *3.10E-43* | | | Whole Blood | | | | |  |  |  |  |  |  |  |  |  |  |  |
|  |  |  |  | *SNF8* | *2.20E-07* | | | Artery Aorta | | | | |  |  |  |  |  |  |  |  |  |  |  |
|  |  |  |  | [*SUMO2P17*](javascript:EqtlsPage.methods.gotoGeneExpression('ENSG00000248278.1')) | *5.90E-09* | | | Artery Tibial | | | | |  |  |  |  |  |  |  |  |  |  |  |
|  | rs12602746 | 0.9683 | 4 | [*ATP5G1*](javascript:EqtlsPage.methods.gotoGeneExpression('ENSG00000159199.13')) | *2.90E-14* | | | [Heart - Left Ventricle](file:///E:\..\..\javascript\EqtlsPage.methods.goTissuePage('Heart_Left_Ventricle')) | | | | |  |  |  |  |  |  |  |  |  |  |  |
|  |  |  |  | *UBE2Z* | *3.10E-43* | | | Whole Blood | | | | |  |  |  |  |  |  |  |  |  |  |  |
|  |  |  |  | *SNF8* | *2.20E-07* | | | Artery Aorta | | | | |  |  |  |  |  |  |  |  |  |  |  |
|  |  |  |  | [*SUMO2P17*](javascript:EqtlsPage.methods.gotoGeneExpression('ENSG00000248278.1')) | *5.90E-09* | | | Artery Tibial | | | | |  |  |  |  |  |  |  |  |  |  |  |
|  | rs60350258 | 0.9683 | 7 | [*ATP5G1*](javascript:EqtlsPage.methods.gotoGeneExpression('ENSG00000159199.13')) | *2.90E-14* | | | [Heart - Left Ventricle](file:///E:\..\..\javascript\EqtlsPage.methods.goTissuePage('Heart_Left_Ventricle')) | | | | |  |  |  |  |  |  |  |  |  |  |  |
|  |  |  |  | *UBE2Z* | *5.60E-43* | | | Whole Blood | | | | |  |  |  |  |  |  |  |  |  |  |  |
|  |  |  |  | *SNF8* | *2.20E-07* | | | Artery Aorta | | | | |  |  |  |  |  |  |  |  |  |  |  |
|  |  |  |  | [*SUMO2P17*](javascript:EqtlsPage.methods.gotoGeneExpression('ENSG00000248278.1')) | *1.00E-08* | | | Artery Tibial | | | | |  |  |  |  |  |  |  |  |  |  |  |
|  | rs62078374 | 0.9683 | 7 | [*ATP5G1*](javascript:EqtlsPage.methods.gotoGeneExpression('ENSG00000159199.13')) | *2.90E-14* | | | [Heart - Left Ventricle](file:///E:\..\..\javascript\EqtlsPage.methods.goTissuePage('Heart_Left_Ventricle')) | | | | |  |  |  |  |  |  |  |  |  |  |  |
|  |  |  |  | *UBE2Z* | *3.10E-43* | | | Whole Blood | | | | |  |  |  |  |  |  |  |  |  |  |  |
|  |  |  |  | *SNF8* | *2.20E-07* | | | Artery Aorta | | | | |  |  |  |  |  |  |  |  |  |  |  |
|  |  |  |  | [*SUMO2P17*](javascript:EqtlsPage.methods.gotoGeneExpression('ENSG00000248278.1')) | *5.90E-09* | | | Artery Tibial | | | | |  |  |  |  |  |  |  |  |  |  |  |
|  | rs12603969 | 0.9683 | 7 | [*ATP5G1*](javascript:EqtlsPage.methods.gotoGeneExpression('ENSG00000159199.13')) | *3.70E-14* | | | [Heart - Left Ventricle](file:///E:\..\..\javascript\EqtlsPage.methods.goTissuePage('Heart_Left_Ventricle')) | | | | |  |  |  |  |  |  |  |  |  |  |  |
|  |  |  |  | *UBE2Z* | *3.00E-42* | | | Whole Blood | | | | |  |  |  |  |  |  |  |  |  |  |  |
|  |  |  |  | *SNF8* | *2.20E-07* | | | Artery Aorta | | | | |  |  |  |  |  |  |  |  |  |  |  |
|  |  |  |  | [*SUMO2P17*](javascript:EqtlsPage.methods.gotoGeneExpression('ENSG00000248278.1')) | *5.90E-09* | | | Artery Tibial | | | | |  |  |  |  |  |  |  |  |  |  |  |
|  | rs12601955 | 0.9683 | 7 | [*ATP5G1*](javascript:EqtlsPage.methods.gotoGeneExpression('ENSG00000159199.13')) | *3.70E-14* | | | [Heart - Left Ventricle](file:///E:\..\..\javascript\EqtlsPage.methods.goTissuePage('Heart_Left_Ventricle')) | | | | |  |  |  |  |  |  |  |  |  |  |  |
|  |  |  |  | *UBE2Z* | *3.00E-42* | | | Whole Blood | | | | |  |  |  |  |  |  |  |  |  |  |  |
|  |  |  |  | *SNF8* | *2.20E-07* | | | Artery Aorta | | | | |  |  |  |  |  |  |  |  |  |  |  |
|  |  |  |  | [*SUMO2P17*](javascript:EqtlsPage.methods.gotoGeneExpression('ENSG00000248278.1')) | *5.90E-09* | | | Artery Tibial | | | | |  |  |  |  |  |  |  |  |  |  |  |
|  | rs62078375 | 0.9683 | 5 | [*ATP5G1*](javascript:EqtlsPage.methods.gotoGeneExpression('ENSG00000159199.13')) | *3.70E-14* | | | [Heart - Left Ventricle](file:///E:\..\..\javascript\EqtlsPage.methods.goTissuePage('Heart_Left_Ventricle')) | | | | |  |  |  |  |  |  |  |  |  |  |  |
|  |  |  |  | *UBE2Z* | *5.40E-42* | | | Whole Blood | | | | |  |  |  |  |  |  |  |  |  |  |  |
|  |  |  |  | *SNF8* | *2.20E-07* | | | Artery Aorta | | | | |  |  |  |  |  |  |  |  |  |  |  |
|  |  |  |  | [*SUMO2P17*](javascript:EqtlsPage.methods.gotoGeneExpression('ENSG00000248278.1')) | *5.90E-09* | | | Artery Tibial | | | | |  |  |  |  |  |  |  |  |  |  |  |
|  | rs4793605 | 0.9683 | 5 | [*ATP5G1*](javascript:EqtlsPage.methods.gotoGeneExpression('ENSG00000159199.13')) | *2.90E-14* | | | [Heart - Left Ventricle](file:///E:\..\..\javascript\EqtlsPage.methods.goTissuePage('Heart_Left_Ventricle')) | | | | |  |  |  |  |  |  |  |  |  |  |  |
|  |  |  |  | *UBE2Z* | *3.10E-43* | | | Whole Blood | | | | |  |  |  |  |  |  |  |  |  |  |  |
|  |  |  |  | *SNF8* | *2.20E-07* | | | Artery Aorta | | | | |  |  |  |  |  |  |  |  |  |  |  |
|  |  |  |  | [*SUMO2P17*](javascript:EqtlsPage.methods.gotoGeneExpression('ENSG00000248278.1')) | *5.90E-09* | | | Artery Tibial | | | | |  |  |  |  |  |  |  |  |  |  |  |
|  | rs12948439 | 0.9681 | 7 | [*ATP5G1*](javascript:EqtlsPage.methods.gotoGeneExpression('ENSG00000159199.13')) | *1.90E-14* | | | [Heart - Left Ventricle](file:///E:\..\..\javascript\EqtlsPage.methods.goTissuePage('Heart_Left_Ventricle')) | | | | |  |  |  |  |  |  |  |  |  |  |  |
|  |  |  |  | *UBE2Z* | *2.60E-44* | | | Whole Blood | | | | |  |  |  |  |  |  |  |  |  |  |  |
|  |  |  |  | *SNF8* | *1.40E-06* | | | Artery Aorta | | | | |  |  |  |  |  |  |  |  |  |  |  |
|  |  |  |  | [*SUMO2P17*](javascript:EqtlsPage.methods.gotoGeneExpression('ENSG00000248278.1')) | *1.40E-08* | | | Artery Tibial | | | | |  |  |  |  |  |  |  |  |  |  |  |
|  | rs12950328 | 0.9681 | 7 | [*ATP5G1*](javascript:EqtlsPage.methods.gotoGeneExpression('ENSG00000159199.13')) | *1.90E-14* | | | [Heart - Left Ventricle](file:///E:\..\..\javascript\EqtlsPage.methods.goTissuePage('Heart_Left_Ventricle')) | | | | |  |  |  |  |  |  |  |  |  |  |  |
|  |  |  |  | *UBE2Z* | *2.60E-44* | | | Whole Blood | | | | |  |  |  |  |  |  |  |  |  |  |  |
|  |  |  |  | *SNF8* | *1.40E-06* | | | Artery Aorta | | | | |  |  |  |  |  |  |  |  |  |  |  |
|  |  |  |  | [*SUMO2P17*](javascript:EqtlsPage.methods.gotoGeneExpression('ENSG00000248278.1')) | *1.40E-08* | | | Artery Tibial | | | | |  |  |  |  |  |  |  |  |  |  |  |
|  | rs12941263 | 0.9681 | 6 | [*ATP5G1*](javascript:EqtlsPage.methods.gotoGeneExpression('ENSG00000159199.13')) | *1.10E-13* | | | [Heart - Left Ventricle](file:///E:\..\..\javascript\EqtlsPage.methods.goTissuePage('Heart_Left_Ventricle')) | | | | |  |  |  |  |  |  |  |  |  |  |  |
|  |  |  |  | *UBE2Z* | *6.40E-39* | | | Whole Blood | | | | |  |  |  |  |  |  |  |  |  |  |  |
|  |  |  |  | *SNF8* | *9.00E-05* | | | Artery Aorta | | | | |  |  |  |  |  |  |  |  |  |  |  |
|  |  |  |  | [*SUMO2P17*](javascript:EqtlsPage.methods.gotoGeneExpression('ENSG00000248278.1')) | *1.30E-08* | | | Artery Tibial | | | | |  |  |  |  |  |  |  |  |  |  |  |
|  | rs62078370 | 0.9644 | 4 | [*ATP5G1*](javascript:EqtlsPage.methods.gotoGeneExpression('ENSG00000159199.13')) | *2.70E-14* | | | [Heart - Left Ventricle](file:///E:\..\..\javascript\EqtlsPage.methods.goTissuePage('Heart_Left_Ventricle')) | | | | |  |  |  |  |  |  |  |  |  |  |  |
|  |  |  |  | *UBE2Z* | *3.10E-43* | | | Whole Blood | | | | |  |  |  |  |  |  |  |  |  |  |  |
|  |  |  |  | *SNF8* | *2.20E-07* | | | Artery Aorta | | | | |  |  |  |  |  |  |  |  |  |  |  |
|  |  |  |  | [*SUMO2P17*](javascript:EqtlsPage.methods.gotoGeneExpression('ENSG00000248278.1')) | *6.10E-09* | | | Artery Tibial | | | | |  |  |  |  |  |  |  |  |  |  |  |
|  | rs12602933 | 0.9644 | 3a | [*ATP5G1*](javascript:EqtlsPage.methods.gotoGeneExpression('ENSG00000159199.13')) | *2.40E-14* | | | [Heart - Left Ventricle](file:///E:\..\..\javascript\EqtlsPage.methods.goTissuePage('Heart_Left_Ventricle')) | | | | |  |  |  |  |  |  |  |  |  |  |  |
|  |  |  |  | *UBE2Z* | *5.30E-43* | | | Whole Blood | | | | |  |  |  |  |  |  |  |  |  |  |  |
|  |  |  |  | *SNF8* | *2.20E-07* | | | Artery Aorta | | | | |  |  |  |  |  |  |  |  |  |  |  |
|  |  |  |  | [*SUMO2P17*](javascript:EqtlsPage.methods.gotoGeneExpression('ENSG00000248278.1')) | *5.90E-09* | | | Artery Tibial | | | | |  |  |  |  |  |  |  |  |  |  |  |
|  | rs4794003 | 0.9644 | 5 | [*ATP5G1*](javascript:EqtlsPage.methods.gotoGeneExpression('ENSG00000159199.13')) | *2.90E-14* | | | [Heart - Left Ventricle](file:///E:\..\..\javascript\EqtlsPage.methods.goTissuePage('Heart_Left_Ventricle')) | | | | |  |  |  |  |  |  |  |  |  |  |  |
|  |  |  |  | *UBE2Z* | *3.10E-43* | | | Whole Blood | | | | |  |  |  |  |  |  |  |  |  |  |  |
|  |  |  |  | *SNF8* | *2.20E-07* | | | Artery Aorta | | | | |  |  |  |  |  |  |  |  |  |  |  |
|  |  |  |  | [*SUMO2P17*](javascript:EqtlsPage.methods.gotoGeneExpression('ENSG00000248278.1')) | *5.90E-09* | | | Artery Tibial | | | | |  |  |  |  |  |  |  |  |  |  |  |
|  | **rs2291725** | **0.9644** | **1f** | [***ATP5G1***](javascript:EqtlsPage.methods.gotoGeneExpression('ENSG00000159199.13')) | ***2.40E-14*** | | | [**Heart - Left Ventricle**](file:///E:\..\..\javascript\EqtlsPage.methods.goTissuePage('Heart_Left_Ventricle')) | | | | |  |  |  |  |  |  |  |  |  |  |  |
|  |  |  |  | ***UBE2Z*** | ***5.30E-43*** | | | **Whole Blood** | | | | |  |  |  |  |  |  |  |  |  |  |  |
|  |  |  |  | ***SNF8*** | ***2.20E-07*** | | | **Artery Aorta** | | | | |  |  |  |  |  |  |  |  |  |  |  |
|  |  |  |  | [***SUMO2P17***](javascript:EqtlsPage.methods.gotoGeneExpression('ENSG00000248278.1')) | ***5.90E-09*** | | | **Artery Tibial** | | | | |  |  |  |  |  |  |  |  |  |  |  |
|  | rs12941262 | 0.9641 | 6 | [*ATP5G1*](javascript:EqtlsPage.methods.gotoGeneExpression('ENSG00000159199.13')) | *2.90E-14* | | | [Heart - Left Ventricle](file:///E:\..\..\javascript\EqtlsPage.methods.goTissuePage('Heart_Left_Ventricle')) | | | | |  |  |  |  |  |  |  |  |  |  |  |
|  |  |  |  | *UBE2Z* | *1.40E-42* | | | Whole Blood | | | | |  |  |  |  |  |  |  |  |  |  |  |
|  |  |  |  | *SNF8* | *1.70E-06* | | | Artery Aorta | | | | |  |  |  |  |  |  |  |  |  |  |  |
|  |  |  |  | [*SUMO2P17*](javascript:EqtlsPage.methods.gotoGeneExpression('ENSG00000248278.1')) | *1.80E-08* | | | Artery Tibial | | | | |  |  |  |  |  |  |  |  |  |  |  |
|  | rs61018047 | 0.9606 | 7 | [*ATP5G1*](javascript:EqtlsPage.methods.gotoGeneExpression('ENSG00000159199.13')) | *2.90E-14* | | | [Heart - Left Ventricle](file:///E:\..\..\javascript\EqtlsPage.methods.goTissuePage('Heart_Left_Ventricle')) | | | | |  |  |  |  |  |  |  |  |  |  |  |
|  |  |  |  | *UBE2Z* | *3.10E-43* | | | Whole Blood | | | | |  |  |  |  |  |  |  |  |  |  |  |
|  |  |  |  | *SNF8* | *2.20E-07* | | | Artery Aorta | | | | |  |  |  |  |  |  |  |  |  |  |  |
|  |  |  |  | [*SUMO2P17*](javascript:EqtlsPage.methods.gotoGeneExpression('ENSG00000248278.1')) | *5.90E-09* | | | Artery Tibial | | | | |  |  |  |  |  |  |  |  |  |  |  |
|  | rs55901257 | 0.9604 | No data |  |  | | | No data | | | | |  |  |  |  |  |  |  |  |  |  |  |
|  | rs3080083 | 0.9561 | 6 |  |  | | | No data | | | | |  |  |  |  |  |  |  |  |  |  |  |
|  | rs2112617 | 0.9529 | 5 | [*ATP5G1*](javascript:EqtlsPage.methods.gotoGeneExpression('ENSG00000159199.13')) | *5.60E-15* | | | [Heart - Left Ventricle](file:///E:\..\..\javascript\EqtlsPage.methods.goTissuePage('Heart_Left_Ventricle')) | | | | |  |  |  |  |  |  |  |  |  |  |  |
|  |  |  |  | *UBE2Z* | *7.30E-45* | | | Whole Blood | | | | |  |  |  |  |  |  |  |  |  |  |  |
|  |  |  |  | *SNF8* | *2.70E-07* | | | Artery Aorta | | | | |  |  |  |  |  |  |  |  |  |  |  |
|  |  |  |  | [*SUMO2P17*](javascript:EqtlsPage.methods.gotoGeneExpression('ENSG00000248278.1')) | *6.60E-09* | | | Artery Tibial | | | | |  |  |  |  |  |  |  |  |  |  |  |
|  | rs62078384 | 0.9225 | 5 | [*ATP5G1*](javascript:EqtlsPage.methods.gotoGeneExpression('ENSG00000159199.13')) | *1.00E-13* | | | [Heart - Left Ventricle](file:///E:\..\..\javascript\EqtlsPage.methods.goTissuePage('Heart_Left_Ventricle')) | | | | |  |  |  |  |  |  |  |  |  |  |  |
|  |  |  |  | *UBE2Z* | *5.20E-40* | | | Whole Blood | | | | |  |  |  |  |  |  |  |  |  |  |  |
|  |  |  |  | *SNF8* | *7.00E-08* | | | Artery Aorta | | | | |  |  |  |  |  |  |  |  |  |  |  |
|  |  |  |  | [*SUMO2P17*](javascript:EqtlsPage.methods.gotoGeneExpression('ENSG00000248278.1')) | *7.90E-08* | | | Artery Tibial | | | | |  |  |  |  |  |  |  |  |  |  |  |
|  | rs62078385 | 0.9225 | 5 | [*ATP5G1*](javascript:EqtlsPage.methods.gotoGeneExpression('ENSG00000159199.13')) | *1.00E-13* | | | [Heart - Left Ventricle](file:///E:\..\..\javascript\EqtlsPage.methods.goTissuePage('Heart_Left_Ventricle')) | | | | |  |  |  |  |  |  |  |  |  |  |  |
|  |  |  |  | *UBE2Z* | *5.20E-40* | | | Whole Blood | | | | |  |  |  |  |  |  |  |  |  |  |  |
|  |  |  |  | *SNF8* | *7.00E-08* | | | Artery Aorta | | | | |  |  |  |  |  |  |  |  |  |  |  |
|  |  |  |  | [*SUMO2P17*](javascript:EqtlsPage.methods.gotoGeneExpression('ENSG00000248278.1')) | *7.90E-08* | | | Artery Tibial | | | | |  |  |  |  |  |  |  |  |  |  |  |
|  | rs12325727 | 0.9133 | 7 | [*ATP5G1*](javascript:EqtlsPage.methods.gotoGeneExpression('ENSG00000159199.13')) | *1.10E-11* | | | [Heart - Left Ventricle](file:///E:\..\..\javascript\EqtlsPage.methods.goTissuePage('Heart_Left_Ventricle')) | | | | |  |  |  |  |  |  |  |  |  |  |  |
|  |  |  |  | *UBE2Z* | *6.60E-38* | | | Whole Blood | | | | |  |  |  |  |  |  |  |  |  |  |  |
|  |  |  |  | *SNF8* | *4.40E-08* | | | Artery Aorta | | | | |  |  |  |  |  |  |  |  |  |  |  |
|  |  |  |  | [*SUMO2P17*](javascript:EqtlsPage.methods.gotoGeneExpression('ENSG00000248278.1')) | *1.30E-07* | | | Artery Tibial | | | | |  |  |  |  |  |  |  |  |  |  |  |
|  | rs58548888 | 0.8938 | No data |  | *No Data* | | |  | | | | |  |  |  |  |  |  |  |  |  |  |  |
|  | rs2546494 | 0.8843 | 7 | [*ATP5G1*](javascript:EqtlsPage.methods.gotoGeneExpression('ENSG00000159199.13')) | *5.60E-12* | | | [Heart - Left Ventricle](file:///E:\..\..\javascript\EqtlsPage.methods.goTissuePage('Heart_Left_Ventricle')) | | | | |  |  |  |  |  |  |  |  |  |  |  |
|  |  |  |  | *UBE2Z* | *7.40E-41* | | | Whole Blood | | | | |  |  |  |  |  |  |  |  |  |  |  |
|  |  |  |  | *SNF8* | *1.40E-06* | | | Artery Aorta | | | | |  |  |  |  |  |  |  |  |  |  |  |
|  |  |  |  | [*SUMO2P17*](javascript:EqtlsPage.methods.gotoGeneExpression('ENSG00000248278.1')) | *3.60E-09* | | | Artery Tibial | | | | |  |  |  |  |  |  |  |  |  |  |  |
|  | rs595767 | 0.8843 | 7 | [*ATP5G1*](javascript:EqtlsPage.methods.gotoGeneExpression('ENSG00000159199.13')) | *7.80E-12* | | | [Heart - Left Ventricle](file:///E:\..\..\javascript\EqtlsPage.methods.goTissuePage('Heart_Left_Ventricle')) | | | | |  |  |  |  |  |  |  |  |  |  |  |
|  |  |  |  | *UBE2Z* | *1.10E-39* | | | Whole Blood | | | | |  |  |  |  |  |  |  |  |  |  |  |
|  |  |  |  | *SNF8* | *4.30E-07* | | | Artery Aorta | | | | |  |  |  |  |  |  |  |  |  |  |  |
|  |  |  |  | [*SUMO2P17*](javascript:EqtlsPage.methods.gotoGeneExpression('ENSG00000248278.1')) | *7.70E-10* | | | Artery Tibial | | | | |  |  |  |  |  |  |  |  |  |  |  |
|  | rs594398 | 0.8843 | 7 | [*ATP5G1*](javascript:EqtlsPage.methods.gotoGeneExpression('ENSG00000159199.13')) | *5.60E-12* | | | [Heart - Left Ventricle](file:///E:\..\..\javascript\EqtlsPage.methods.goTissuePage('Heart_Left_Ventricle')) | | | | |  |  |  |  |  |  |  |  |  |  |  |
|  |  |  |  | *UBE2Z* | *6.90E-41* | | | Whole Blood | | | | |  |  |  |  |  |  |  |  |  |  |  |
|  |  |  |  | *SNF8* | *1.40E-06* | | | Artery Aorta | | | | |  |  |  |  |  |  |  |  |  |  |  |
|  |  |  |  | [*SUMO2P17*](javascript:EqtlsPage.methods.gotoGeneExpression('ENSG00000248278.1')) | *3.60E-09* | | | Artery Tibial | | | | |  |  |  |  |  |  |  |  |  |  |  |
|  | **rs4794004** | **0.8792** | **1d** | [***ATP5G1***](javascript:EqtlsPage.methods.gotoGeneExpression('ENSG00000159199.13')) | ***1.60E-10*** | | | [**Heart - Left Ventricle**](file:///E:\..\..\javascript\EqtlsPage.methods.goTissuePage('Heart_Left_Ventricle')) | | | | |  |  |  |  |  |  |  |  |  |  |  |
|  |  |  |  | ***UBE2Z*** | ***4.10E-34*** | | | **Whole Blood** | | | | |  |  |  |  |  |  |  |  |  |  |  |
|  |  |  |  | ***SNF8*** | ***8.90E-07*** | | | **Artery Aorta** | | | | |  |  |  |  |  |  |  |  |  |  |  |
|  |  |  |  | [*SUMO2P17*](javascript:EqtlsPage.methods.gotoGeneExpression('ENSG00000248278.1')) | *2.00E-07* | | | Artery Tibial | | | | |  |  |  |  |  |  |  |  |  |  |  |
|  | rs2291726 | 0.8792 | 4 | [*ATP5G1*](javascript:EqtlsPage.methods.gotoGeneExpression('ENSG00000159199.13')) | *7.00E-11* | | | [Heart - Left Ventricle](file:///E:\..\..\javascript\EqtlsPage.methods.goTissuePage('Heart_Left_Ventricle')) | | | | |  |  |  |  |  |  |  |  |  |  |  |
|  |  |  |  | *UBE2Z* | *4.60E-34* | | | Whole Blood | | | | |  |  |  |  |  |  |  |  |  |  |  |
|  |  |  |  | *SNF8* | *7.70E-07* | | | Artery Aorta | | | | |  |  |  |  |  |  |  |  |  |  |  |
|  |  |  |  | [*SUMO2P17*](javascript:EqtlsPage.methods.gotoGeneExpression('ENSG00000248278.1')) | *1.30E-07* | | | Artery Tibial | | | | |  |  |  |  |  |  |  |  |  |  |  |
|  | rs1965983 | 0.8792 | 7 | [*ATP5G1*](javascript:EqtlsPage.methods.gotoGeneExpression('ENSG00000159199.13')) | *1.10E-10* | | | [Heart - Left Ventricle](file:///E:\..\..\javascript\EqtlsPage.methods.goTissuePage('Heart_Left_Ventricle')) | | | | |  |  |  |  |  |  |  |  |  |  |  |
|  |  |  |  | *UBE2Z* | *6.10E-33* | | | Whole Blood | | | | |  |  |  |  |  |  |  |  |  |  |  |
|  |  |  |  | *SNF8* | *9.50E-07* | | | Artery Aorta | | | | |  |  |  |  |  |  |  |  |  |  |  |
|  |  |  |  | [*SUMO2P17*](javascript:EqtlsPage.methods.gotoGeneExpression('ENSG00000248278.1')) | *9.80E-08* | | | Artery Tibial | | | | |  |  |  |  |  |  |  |  |  |  |  |
|  | rs4416061 | 0.8792 | 7 | [*ATP5G1*](javascript:EqtlsPage.methods.gotoGeneExpression('ENSG00000159199.13')) | *1.10E-10* | | | [Heart - Left Ventricle](file:///E:\..\..\javascript\EqtlsPage.methods.goTissuePage('Heart_Left_Ventricle')) | | | | |  |  |  |  |  |  |  |  |  |  |  |
|  |  |  |  | *UBE2Z* | *4.00E-33* | | | Whole Blood | | | | |  |  |  |  |  |  |  |  |  |  |  |
|  |  |  |  | *SNF8* | *9.50E-07* | | | Artery Aorta | | | | |  |  |  |  |  |  |  |  |  |  |  |
|  |  |  |  | [*SUMO2P17*](javascript:EqtlsPage.methods.gotoGeneExpression('ENSG00000248278.1')) | *1.40E-07* | | | Artery Tibial | | | | |  |  |  |  |  |  |  |  |  |  |  |
|  | rs1973408 | 0.8792 | 6 | [*ATP5G1*](javascript:EqtlsPage.methods.gotoGeneExpression('ENSG00000159199.13')) | *1.10E-10* | | | [Heart - Left Ventricle](file:///E:\..\..\javascript\EqtlsPage.methods.goTissuePage('Heart_Left_Ventricle')) | | | | |  |  |  |  |  |  |  |  |  |  |  |
|  |  |  |  | *UBE2Z* | *4.00E-33* | | | Whole Blood | | | | |  |  |  |  |  |  |  |  |  |  |  |
|  |  |  |  | *SNF8* | *9.50E-07* | | | Artery Aorta | | | | |  |  |  |  |  |  |  |  |  |  |  |
|  |  |  |  | [*SUMO2P17*](javascript:EqtlsPage.methods.gotoGeneExpression('ENSG00000248278.1')) | *1.40E-07* | | | Artery Tibial | | | | |  |  |  |  |  |  |  |  |  |  |  |
|  | rs6504588 | 0.8792 | 7 | [*ATP5G1*](javascript:EqtlsPage.methods.gotoGeneExpression('ENSG00000159199.13')) | *1.10E-10* | | | [Heart - Left Ventricle](file:///E:\..\..\javascript\EqtlsPage.methods.goTissuePage('Heart_Left_Ventricle')) | | | | |  |  |  |  |  |  |  |  |  |  |  |
|  |  |  |  | *UBE2Z* | *4.00E-33* | | | Whole Blood | | | | |  |  |  |  |  |  |  |  |  |  |  |
|  |  |  |  | *SNF8* | *9.50E-07* | | | Artery Aorta | | | | |  |  |  |  |  |  |  |  |  |  |  |
|  |  |  |  | [*SUMO2P17*](javascript:EqtlsPage.methods.gotoGeneExpression('ENSG00000248278.1')) | *1.40E-07* | | | Artery Tibial | | | | |  |  |  |  |  |  |  |  |  |  |  |
|  | rs4794005 | 0.8753 | 7 | [*ATP5G1*](javascript:EqtlsPage.methods.gotoGeneExpression('ENSG00000159199.13')) | *No data* | | | [Heart - Left Ventricle](file:///E:\..\..\javascript\EqtlsPage.methods.goTissuePage('Heart_Left_Ventricle')) | | | | |  |  |  |  |  |  |  |  |  |  |  |
|  |  |  |  | *UBE2Z* |  | | | Whole Blood | | | | |  |  |  |  |  |  |  |  |  |  |  |
|  |  |  |  | *SNF8* |  | | | Artery Aorta | | | | |  |  |  |  |  |  |  |  |  |  |  |
|  |  |  |  | [*SUMO2P17*](javascript:EqtlsPage.methods.gotoGeneExpression('ENSG00000248278.1')) |  | | | Artery Tibial | | | | |  |  |  |  |  |  |  |  |  |  |  |
|  | rs4794006 | 0.8678 | 6 | [*ATP5G1*](javascript:EqtlsPage.methods.gotoGeneExpression('ENSG00000159199.13')) | *3.10E-10* | | | [Heart - Left Ventricle](file:///E:\..\..\javascript\EqtlsPage.methods.goTissuePage('Heart_Left_Ventricle')) | | | | |  |  |  |  |  |  |  |  |  |  |  |
|  |  |  |  | *UBE2Z* | *8.70E-35* | | | Whole Blood | | | | |  |  |  |  |  |  |  |  |  |  |  |
|  |  |  |  | *SNF8* | *2.30E-06* | | | Artery Aorta | | | | |  |  |  |  |  |  |  |  |  |  |  |
|  |  |  |  | [*SUMO2P17*](javascript:EqtlsPage.methods.gotoGeneExpression('ENSG00000248278.1')) | *9.90E-08* | | | Artery Tibial | | | | |  |  |  |  |  |  |  |  |  |  |  |
|  | rs9747749 | 0.8678 | 6 | [*ATP5G1*](javascript:EqtlsPage.methods.gotoGeneExpression('ENSG00000159199.13')) | *No data* | | | [Heart - Left Ventricle](file:///E:\..\..\javascript\EqtlsPage.methods.goTissuePage('Heart_Left_Ventricle')) | | | | |  |  |  |  |  |  |  |  |  |  |  |
|  |  |  |  | *UBE2Z* |  | | | Whole Blood | | | | |  |  |  |  |  |  |  |  |  |  |  |
|  |  |  |  | *SNF8* |  | | | Artery Aorta | | | | |  |  |  |  |  |  |  |  |  |  |  |
|  |  |  |  | [*SUMO2P17*](javascript:EqtlsPage.methods.gotoGeneExpression('ENSG00000248278.1')) |  | | | Artery Tibial | | | | |  |  |  |  |  |  |  |  |  |  |  |
|  | rs11305666 | 0.8617 | No data | [*ATP5G1*](javascript:EqtlsPage.methods.gotoGeneExpression('ENSG00000159199.13')) | *No data* | | | [Heart - Left Ventricle](file:///E:\..\..\javascript\EqtlsPage.methods.goTissuePage('Heart_Left_Ventricle')) | | | | |  |  |  |  |  |  |  |  |  |  |  |
|  |  |  |  | *UBE2Z* |  | | | Whole Blood | | | | |  |  |  |  |  |  |  |  |  |  |  |
|  |  |  |  | *SNF8* |  | | | Artery Aorta | | | | |  |  |  |  |  |  |  |  |  |  |  |
|  |  |  |  | [*SUMO2P17*](javascript:EqtlsPage.methods.gotoGeneExpression('ENSG00000248278.1')) |  | | | Artery Tibial | | | | |  |  |  |  |  |  |  |  |  |  |  |
|  | rs8078994 | 0.8602 | 7 | [*ATP5G1*](javascript:EqtlsPage.methods.gotoGeneExpression('ENSG00000159199.13')) | *8.30E-10* | | | [Heart - Left Ventricle](file:///E:\..\..\javascript\EqtlsPage.methods.goTissuePage('Heart_Left_Ventricle')) | | | | |  |  |  |  |  |  |  |  |  |  |  |
|  |  |  |  | *UBE2Z* | *3.80E-32* | | | Whole Blood | | | | |  |  |  |  |  |  |  |  |  |  |  |
|  |  |  |  | *SNF8* | *1.90E-06* | | | Artery Aorta | | | | |  |  |  |  |  |  |  |  |  |  |  |
|  |  |  |  | [*SUMO2P17*](javascript:EqtlsPage.methods.gotoGeneExpression('ENSG00000248278.1')) | *3.10E-07* | | | Artery Tibial | | | | |  |  |  |  |  |  |  |  |  |  |  |
|  | rs8079149 | 0.8602 | 7 | [*ATP5G1*](javascript:EqtlsPage.methods.gotoGeneExpression('ENSG00000159199.13')) | *8.30E-10* | | | [Heart - Left Ventricle](file:///E:\..\..\javascript\EqtlsPage.methods.goTissuePage('Heart_Left_Ventricle')) | | | | |  |  |  |  |  |  |  |  |  |  |  |
|  |  |  |  | *UBE2Z* | *3.50E-32* | | | Whole Blood | | | | |  |  |  |  |  |  |  |  |  |  |  |
|  |  |  |  | *SNF8* | *9.60E-07* | | | Artery Aorta | | | | |  |  |  |  |  |  |  |  |  |  |  |
|  |  |  |  | [*SUMO2P17*](javascript:EqtlsPage.methods.gotoGeneExpression('ENSG00000248278.1')) | *3.10E-07* | | | Artery Tibial | | | | |  |  |  |  |  |  |  |  |  |  |  |
|  | rs11079848 | 0.8564 | 6 | [*ATP5G1*](javascript:EqtlsPage.methods.gotoGeneExpression('ENSG00000159199.13')) | *4.90E-10* | | | [Heart - Left Ventricle](file:///E:\..\..\javascript\EqtlsPage.methods.goTissuePage('Heart_Left_Ventricle')) | | | | |  |  |  |  |  |  |  |  |  |  |  |
|  |  |  |  | *UBE2Z* | *2.80E-32* | | | Whole Blood | | | | |  |  |  |  |  |  |  |  |  |  |  |
|  |  |  |  | *SNF8* | *1.90E-06* | | | Artery Aorta | | | | |  |  |  |  |  |  |  |  |  |  |  |
|  |  |  |  | [*SUMO2P17*](javascript:EqtlsPage.methods.gotoGeneExpression('ENSG00000248278.1')) | *2.40E-07* | | | Artery Tibial | | | | |  |  |  |  |  |  |  |  |  |  |  |
|  | rs9903470 | 0.8564 | 7 | [*ATP5G1*](javascript:EqtlsPage.methods.gotoGeneExpression('ENSG00000159199.13')) | *2.60E-10* | | | [Heart - Left Ventricle](file:///E:\..\..\javascript\EqtlsPage.methods.goTissuePage('Heart_Left_Ventricle')) | | | | |  |  |  |  |  |  |  |  |  |  |  |
|  |  |  |  | *UBE2Z* | *3.50E-32* | | | Whole Blood | | | | |  |  |  |  |  |  |  |  |  |  |  |
|  |  |  |  | *SNF8* | *1.10E-06* | | | Artery Aorta | | | | |  |  |  |  |  |  |  |  |  |  |  |
|  |  |  |  | [*SUMO2P17*](javascript:EqtlsPage.methods.gotoGeneExpression('ENSG00000248278.1')) | *1.20E-07* | | | Artery Tibial | | | | |  |  |  |  |  |  |  |  |  |  |  |
|  | rs937301 | 0.8564 | 5 | [*ATP5G1*](javascript:EqtlsPage.methods.gotoGeneExpression('ENSG00000159199.13')) | *4.50E-10* | | | [Heart - Left Ventricle](file:///E:\..\..\javascript\EqtlsPage.methods.goTissuePage('Heart_Left_Ventricle')) | | | | |  |  |  |  |  |  |  |  |  |  |  |
|  |  |  |  | *UBE2Z* | *2.80E-32* | | | Whole Blood | | | | |  |  |  |  |  |  |  |  |  |  |  |
|  |  |  |  | *SNF8* | *1.90E-06* | | | Artery Aorta | | | | |  |  |  |  |  |  |  |  |  |  |  |
|  |  |  |  | [*SUMO2P17*](javascript:EqtlsPage.methods.gotoGeneExpression('ENSG00000248278.1')) | *2.40E-07* | | | Artery Tibial | | | | |  |  |  |  |  |  |  |  |  |  |  |
|  | **rs3848460** | **0.8564** | **1f** | [***ATP5G1***](javascript:EqtlsPage.methods.gotoGeneExpression('ENSG00000159199.13')) | ***4.50E-10*** | | | [**Heart - Left Ventricle**](file:///E:\..\..\javascript\EqtlsPage.methods.goTissuePage('Heart_Left_Ventricle')) | | | | |  |  |  |  |  |  |  |  |  |  |  |
|  |  |  |  | ***UBE2Z*** | ***2.80E-32*** | | | **Whole Blood** | | | | |  |  |  |  |  |  |  |  |  |  |  |
|  |  |  |  | ***SNF8*** | ***1.90E-06*** | | | **Artery Aorta** | | | | |  |  |  |  |  |  |  |  |  |  |  |
|  |  |  |  | [***SUMO2P17***](javascript:EqtlsPage.methods.gotoGeneExpression('ENSG00000248278.1')) | ***2.40E-07*** | | | **Artery Tibial** | | | | |  |  |  |  |  |  |  |  |  |  |  |
|  | rs9910001 | 0.8526 | 6 | [*ATP5G1*](javascript:EqtlsPage.methods.gotoGeneExpression('ENSG00000159199.13')) | *4.90E-10* | | | [Heart - Left Ventricle](file:///E:\..\..\javascript\EqtlsPage.methods.goTissuePage('Heart_Left_Ventricle')) | | | | |  |  |  |  |  |  |  |  |  |  |  |
|  |  |  |  | *UBE2Z* | *2.00E-32* | | | Whole Blood | | | | |  |  |  |  |  |  |  |  |  |  |  |
|  |  |  |  | *SNF8* | *1.90E-06* | | | Artery Aorta | | | | |  |  |  |  |  |  |  |  |  |  |  |
|  |  |  |  | [*SUMO2P17*](javascript:EqtlsPage.methods.gotoGeneExpression('ENSG00000248278.1')) | *2.40E-07* | | | Artery Tibial | | | | |  |  |  |  |  |  |  |  |  |  |  |
|  | rs9901810 | 0.8526 | 6 | [*ATP5G1*](javascript:EqtlsPage.methods.gotoGeneExpression('ENSG00000159199.13')) | *4.90E-10* | | | [Heart - Left Ventricle](file:///E:\..\..\javascript\EqtlsPage.methods.goTissuePage('Heart_Left_Ventricle')) | | | | |  |  |  |  |  |  |  |  |  |  |  |
|  |  |  |  | *UBE2Z* | *2.00E-32* | | | Whole Blood | | | | |  |  |  |  |  |  |  |  |  |  |  |
|  |  |  |  | *SNF8* | *1.90E-06* | | | Artery Aorta | | | | |  |  |  |  |  |  |  |  |  |  |  |
|  |  |  |  | [*SUMO2P17*](javascript:EqtlsPage.methods.gotoGeneExpression('ENSG00000248278.1')) | *2.40E-07* | | | Artery Tibial | | | | |  |  |  |  |  |  |  |  |  |  |  |
|  | rs3895874 | 0.849 | 4 | [*ATP5G1*](javascript:EqtlsPage.methods.gotoGeneExpression('ENSG00000159199.13')) | *9.70E-08* | | | [Heart - Left Ventricle](file:///E:\..\..\javascript\EqtlsPage.methods.goTissuePage('Heart_Left_Ventricle')) | | | | |  |  |  |  |  |  |  |  |  |  |  |
|  |  |  |  | *UBE2Z* | *3.60E-30* | | | Whole Blood | | | | |  |  |  |  |  |  |  |  |  |  |  |
|  |  |  |  | *SNF8* | *5.70E-06* | | | Artery Aorta | | | | |  |  |  |  |  |  |  |  |  |  |  |
|  |  |  |  | [*SUMO2P17*](javascript:EqtlsPage.methods.gotoGeneExpression('ENSG00000248278.1')) | *5.00E-07* | | | Artery Tibial | | | | |  |  |  |  |  |  |  |  |  |  |  |
|  | rs12232531 | 0.8346 | 6 | [*ATP5G1*](javascript:EqtlsPage.methods.gotoGeneExpression('ENSG00000159199.13')) | *No data* | | | [Heart - Left Ventricle](file:///E:\..\..\javascript\EqtlsPage.methods.goTissuePage('Heart_Left_Ventricle')) | | | | |  |  |  |  |  |  |  |  |  |  |  |
|  |  |  |  | *UBE2Z* |  | | | Whole Blood | | | | |  |  |  |  |  |  |  |  |  |  |  |
|  |  |  |  | *SNF8* |  | | | Artery Aorta | | | | |  |  |  |  |  |  |  |  |  |  |  |
|  |  |  |  | [*SUMO2P17*](javascript:EqtlsPage.methods.gotoGeneExpression('ENSG00000248278.1')) |  | | | Artery Tibial | | | | |  |  |  |  |  |  |  |  |  |  |  |
|  | rs146301099 | 0.8306 | 5 | [*ATP5G1*](javascript:EqtlsPage.methods.gotoGeneExpression('ENSG00000159199.13')) | *No data* | | | [Heart - Left Ventricle](file:///E:\..\..\javascript\EqtlsPage.methods.goTissuePage('Heart_Left_Ventricle')) | | | | |  |  |  |  |  |  |  |  |  |  |  |
|  |  |  |  | *UBE2Z* |  | | | Whole Blood | | | | |  |  |  |  |  |  |  |  |  |  |  |
|  |  |  |  | *SNF8* |  | | | Artery Aorta | | | | |  |  |  |  |  |  |  |  |  |  |  |
|  |  |  |  | [*SUMO2P17*](javascript:EqtlsPage.methods.gotoGeneExpression('ENSG00000248278.1')) |  | | | Artery Tibial | | | | |  |  |  |  |  |  |  |  |  |  |  |
|  | **rs9894220** | **0.8149** | **1f** | [***ATP5G1***](javascript:EqtlsPage.methods.gotoGeneExpression('ENSG00000159199.13')) | ***1.40E-21*** | | | [**Heart - Left Ventricle**](file:///E:\..\..\javascript\EqtlsPage.methods.goTissuePage('Heart_Left_Ventricle')) | | | | |  |  |  |  |  |  |  |  |  |  |  |
|  |  |  |  | ***UBE2Z*** | ***3.00E-37*** | | | **Whole Blood** | | | | |  |  |  |  |  |  |  |  |  |  |  |
|  |  |  |  | ***SNF8*** | ***5.20E-08*** | | | **Artery Aorta** | | | | |  |  |  |  |  |  |  |  |  |  |  |
|  |  |  |  | [***SUMO2P17***](javascript:EqtlsPage.methods.gotoGeneExpression('ENSG00000248278.1')) | ***2.10E-07*** | | | **Artery Tibial** | | | | |  |  |  |  |  |  |  |  |  |  |  |
|  | **rs999474** | **0.8149** | **1f** | [***ATP5G1***](javascript:EqtlsPage.methods.gotoGeneExpression('ENSG00000159199.13')) | ***1.40E-21*** | | | [**Heart - Left Ventricle**](file:///E:\..\..\javascript\EqtlsPage.methods.goTissuePage('Heart_Left_Ventricle')) | | | | |  |  |  |  |  |  |  |  |  |  |  |
|  |  |  |  | ***UBE2Z*** | ***3.00E-37*** | | | **Whole Blood** | | | | |  |  |  |  |  |  |  |  |  |  |  |
|  |  |  |  | ***SNF8*** | ***5.20E-08*** | | | **Artery Aorta** | | | | |  |  |  |  |  |  |  |  |  |  |  |
|  |  |  |  | [***SUMO2P17***](javascript:EqtlsPage.methods.gotoGeneExpression('ENSG00000248278.1')) | ***2.10E-07*** | | | **Artery Tibial** | | | | |  |  |  |  |  |  |  |  |  |  |  |
|  | rs28528789 | 0.8149 | 4 | [*ATP5G1*](javascript:EqtlsPage.methods.gotoGeneExpression('ENSG00000159199.13')) | *1.10E-21* | | | [Heart - Left Ventricle](file:///E:\..\..\javascript\EqtlsPage.methods.goTissuePage('Heart_Left_Ventricle')) | | | | |  |  |  |  |  |  |  |  |  |  |  |
|  |  |  |  | *UBE2Z* | *3.00E-37* | | | Whole Blood | | | | |  |  |  |  |  |  |  |  |  |  |  |
|  |  |  |  | *SNF8* | *6.10E-08* | | | Artery Aorta | | | | |  |  |  |  |  |  |  |  |  |  |  |
|  |  |  |  | [*SUMO2P17*](javascript:EqtlsPage.methods.gotoGeneExpression('ENSG00000248278.1')) | *3.00E-07* | | | Artery Tibial | | | | |  |  |  |  |  |  |  |  |  |  |  |
|  | rs11657238 | 0.8149 | 4 | [*ATP5G1*](javascript:EqtlsPage.methods.gotoGeneExpression('ENSG00000159199.13')) | *7.80E-22* | | | [Heart - Left Ventricle](file:///E:\..\..\javascript\EqtlsPage.methods.goTissuePage('Heart_Left_Ventricle')) | | | | |  |  |  |  |  |  |  |  |  |  |  |
|  |  |  |  | *UBE2Z* | *5.90E-38* | | | Whole Blood | | | | |  |  |  |  |  |  |  |  |  |  |  |
|  |  |  |  | *SNF8* | *1.10E-07* | | | Artery Aorta | | | | |  |  |  |  |  |  |  |  |  |  |  |
|  |  |  |  | [*SUMO2P17*](javascript:EqtlsPage.methods.gotoGeneExpression('ENSG00000248278.1')) | *3.80E-07* | | | Artery Tibial | | | | |  |  |  |  |  |  |  |  |  |  |  |
|  | rs12940898 | 0.8149 | 7 | [*ATP5G1*](javascript:EqtlsPage.methods.gotoGeneExpression('ENSG00000159199.13')) | *1.70E-21* | | | [Heart - Left Ventricle](file:///E:\..\..\javascript\EqtlsPage.methods.goTissuePage('Heart_Left_Ventricle')) | | | | |  |  |  |  |  |  |  |  |  |  |  |
|  |  |  |  | *UBE2Z* | *1.30E-37* | | | Whole Blood | | | | |  |  |  |  |  |  |  |  |  |  |  |
|  |  |  |  | *SNF8* | *1.50E-07* | | | Artery Aorta | | | | |  |  |  |  |  |  |  |  |  |  |  |
|  |  |  |  | [*SUMO2P17*](javascript:EqtlsPage.methods.gotoGeneExpression('ENSG00000248278.1')) | *3.60E-07* | | | Artery Tibial | | | | |  |  |  |  |  |  |  |  |  |  |  |
|  | rs9894239 | 0.8149 | 7 | [*ATP5G1*](javascript:EqtlsPage.methods.gotoGeneExpression('ENSG00000159199.13')) | *1.40E-21* | | | [Heart - Left Ventricle](file:///E:\..\..\javascript\EqtlsPage.methods.goTissuePage('Heart_Left_Ventricle')) | | | | |  | | |  | | | |  |  |  |  |
|  |  |  |  | [*UBE2Z*](javascript:EqtlsPage.methods.gotoGeneExpression('ENSG00000159202.17')) | *3.00E-37* | | | Whole Blood | | | | |  | | |  | | | |  |  |  |  |
|  |  |  |  | [*SNF8*](javascript:EqtlsPage.methods.gotoGeneExpression('ENSG00000159210.9')) | *1.50E-06* | | | Artery Aorta | | | | |  | | |  | | | |  |  |  |  |
|  |  |  |  | *SUMO2P17* | *2.10E-07* | | | Artery Tibial | | | | |  | | |  | | | |  |  |  |  |
|  | rs201105813 | 0.8047 | No data | *No data* |  | | |  | | | | |  | | |  | | | |  |  |  |  |
